# YATSIDroid: an android malware detection framework based on artificial immune system

**DOI:** 10.1038/s41598-026-54281-x

**Published:** 2026-06-06

**Authors:** Arvind Mahindru, Himani Arora, Leila Jamel, Abhinav Kumar, Shikha Marwaha, Demmelash Mollalign Moges, Fahima Hajjej

**Affiliations:** 1 Department of Computer Science and Applications, D.A.V. University, Sarmastpur, 144012 Jalandhar, India; 2 Department of Mathematics, Guru Nanak Dev University, Amritsar, 143005 Punjab India; 3 Department of Information Systems, College of Computer and Information Sciences, Princess Nourah bint Abdulrahman University, P.O. Box 84428, 11671 Riyadh, Saudi Arabia; 4 Department of Nuclear and Renewable Energy, Ural Federal University , Yekaterinburg, 620002 Russia; 5 Department of Technical Sciences, Western Caspian University, Baku, Azerbaijan; 6 Department of Computer Science and Engineering, Shiv Nadar University, Chennai, India; 7 Department of Mathematics, Hawassa University, P.O. Box 05, Hawassa, Ethiopia; 8 Centre for Research Impact & Outcome, Chitkara University Institute of Engineering and Technology, Chitkara University, Rajpura, 140401, Punjab, India

**Keywords:** API calls, Reliable Semi-supervised classification, Machine learning, Feature ranking methods, Android apps, YATSI, Permissions, Computational biology and bioinformatics, Engineering, Mathematics and computing

## Abstract

Malware detection has become a challenging issue in the field of software engineering. There is an exponential growth of smartphones and apps in the market. Android has gained popularity due to the number of free apps available in its official market. The functionality of apps relies on the permission model. Cybercriminals exploit app permissions to create and distribute malware-infected apps, which they publish in deceptive repositories for smartphone users. This study proposes a malware detection model that works on the principle of feature ranking approaches and a two-stage semi-supervised machine learning classifier *YATSI* (Yet Another Two-Stage Idea). YATSI is a meta-algorithm that permits distinct machine learning algorithms to apply in the first stage. The experimental result reveals that our proposed algorithm, i.e., YATSIDroid (Artificial Immune System’s Paradigm with YATSI), improves the performance in identifying the malware-infected apps by using the limited labeled dataset as an input. The empirical result shows that YATSIDroid can detect 98.5% of malware-infected apps with 60% of the labeled dataset.

## Introduction

Smartphone usage is increasing at an exponential rate these days (https://www.statista.com/statistics/330695/number-of-smartphone-users-worldwide/). A variety of operating systems are available on the market to operate smartphones. In addition to being open-source and having numerous apps, in its Official store (https://play.google.com/store), Android has captured the market share of 85% (https://hostingtribunal.com/blog/operating-systems-market-share/#gref). According to the survey (https://appinventiv.com/blog/google-play-store-statistics/), 3.3 million Android apps are still in the official Google market. The permission-model idea governs how Android apps work (https://developer.android.com/training/permissions/usage-notes). During the installation and execution phase, Android apps need certain permissions or a set of permissions to work effectively. Android permissions are divided into two groups, i.e., dangerous permissions or normal permissions (List of normal and dangerous permissions are available at https://developer.android.com/guide/topics/permissions/overview). An Android itself grants normal permissions. It does not ask the user to grant permission. Dangerous permissions, on the other hand, require users to deny or approve the permission requested by an app. In early Android operating system versions, the ability to grant or revoke permissions was not present. This facility is available in Android 6.0 (API 23) and higher versions of Android released in the market so far.

By using the privilege of Android permissions, Cyber-crooks develop malware-infected apps and upload them on promised repositories. Smartphone users download the apps from these repositories and install them. Cybercriminals exploit malware-infected apps to access users’ private information for their gain. Google introduced Google Bouncer (https://mashable.com/2012/02/02/google-bouncer-for-android/) and Google play (https://en.wikipedia.org/wiki/Google_Play) to determine the malware-infected apps. But they failed to identify malware-infected apps^1,2^. According to a published report (https://www.businesswire.com/news/home/20190225005402/en/McAfee-Mobile-Threat-Report-Unveils-550-Increase), one malware-infected app is uploaded to any repository every 10 s. The challenge facing researchers and academics now is to identify malware-infected apps and mitigate their impact. A traditional signature-based detection technique, similar to those used on desktop computers, can only identify known malware for which definitions are already available. The signature-based detection approach struggles to identify unknown malware because its database contains a limited number of signatures. In the previous studies^2,3^, researchers suggested several approaches or frameworks to detect malicious apps from the real world with the help of a labeled dataset. This research paper presents an approach grounded in the principles of semi-supervised machine learning.

To develop a framework, the supervised machine learning approach employs a labeled dataset, including class labels and features. However, collecting labels from real-world apps is a very time-consuming process. The best way to address this problem is to create semi-supervised machine learning algorithms that utilize both labeled and unlabeled data in the training process. In machine learning algorithms, the classifiers that help to build the model by using labeled and unlabeled data are called semi-supervised classifiers. Developing an accurate semi-supervised machine learning algorithm for any problem domain is quite challenging. In the literature^4,5^, several researchers and academicians implement semi-supervised algorithms for malware detection from desktop computers, and in smartphones too^6^. However, they just used a small set of data in their research. To overcome this limitation, in this research paper, a semi-supervised machine learning algorithm is implemented on half a million of Android apps.

In this research paper, YATSI (Yet Another Two-Stage Idea) is implemented with several high-performance machine learning algorithms. YATSI was proposed by Driessens et al.,^7^ in the year 2006 for evaluating the performance of distinct datasets by using accuracy as a parameter. In their research paper, they illustrated that YATSI improves the performance of various machine learning algorithms with unlabeled datasets. This research advances the state-of-the-art technology as described in the literature because it not only concentrates on malware detection from Android devices with a small number of labeled datasets but also helps in determining the ideal number of labeled datasets required to create an effective Android malware detection framework.

**Fig. 1:**
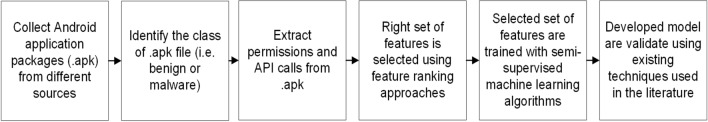
Diagrammatically representation of proposed work.

Figure 1 illustrates the six phases utilized in this research paper to develop an efficient malware detection system. In the initial phase, Android application packages (.apk files) are collected from multiple repositories. In the second phase, features like API calls and permissions are extracted from the collected .apk files. Relevant features are then selected using six different feature selection methods. Based on the selected features, various malware detection models are created using five different supervised and semi-supervised machine learning techniques. Furthermore, the effectiveness of our models is assessed using two performance metrics: F-measure and Accuracy. Finally, the performance of our proposed malware detection framework is compared against current methodologies proposed in the literature.

The novel and unique contribution of this study are as follows:

- In this article, a machine learning algorithm is proposed that worked on the principle of an Artificial immune system (AIS), which is a rising biologically inspired computing paradigm. The proposed algorithm has the property of learning the behavior from its immediate neighbor. By using this property, the proposed algorithm can easily learn the dynamic behavior of known malware families, which further helps in detecting unknown malware families.
- The proposed approach is capable of identifying 98.5% malware-infected apps with 60% of the labeled dataset, as compared to the approaches proposed in the literature. That were trained with 100% of the labeled dataset.
- The proposed malware detection method can identify malware-infected apps in less time compared to various antivirus scanners currently available on the market.
- Several Android malware detection frameworks have been presented in the literature. However, the proposed algorithm in this work is capable of determining the optimal number of labeled datasets needed to develop an efficient malware detection model.
- This is the first study to utilize half a million Android apps to develop an effective malware detection model.
- In this research paper, an Android malware detection framework is developed using relevant features selected through feature ranking approaches, and based on a semi-supervised machine learning algorithm. Our empirical results demonstrate that the proposed framework can detect 98.5% of unknown malware families.
- To determine features selected by feature selection techniques have relevant difference or not, *t*-test analysis is applied in this study.

The rest of this work is summarized as follows. Section “Related work” describes the related work done so far in the field of Android malware detection. Further, based on the gaps, research questions are developed to answer in this article. The formulation of the dataset is discussed in Section “Experimental dataset”. Distinct feature selection approaches that were implemented in this article are described in section “Implemented feature selection approaches”. Section “Machine learning techniques” introduces the proposed immune-based machine learning technique. Section “Evaluation methods for the proposed framework” reviews existing malware detection techniques from the literature that are used to evaluate our approach. In section “Performance parameters considered in this research paper”, various performance parameters are discussed. Section “Experimental setup” details the experimental setup for developing an Android malware detection framework. The experimental results are presented in sections “Experimental results”, and “Conclusion” concludes with a discussion of our findings and suggestions for future work.

## Related work

This section discusses previously developed frameworks and approaches for Android malware detection. It also highlights the gaps in the existing literature and outlines the research questions that will be addressed in this study.

Burguera et al.^8^ suggested “Crowdroid” a machine learning-based framework that used system calls as features and identify Trojan malware from a smartphone-based on Android as an operating system. Shabtai et al.^9^ proposed “Andromaly” which monitors the behavior of both the smartphone user and smartphone by selecting several parameters from CPU usage to sensors activities. Features are selected by using distinct feature selection algorithms to train distinct machine learning classifiers. The authors were having a limited dataset of malicious Android apps for evaluating the performance of their proposed framework. Aafer et al.^10^ proposed “DroidAPIMiner” which is based on the principle of k-nearest neighbor (KNN) classifier and achieved an accuracy of 99% on a limited dataset. They consider API as features and train models with the refinement of the features which helped to detect the class of an Android app. Zarni Aung and Win Zaw^11^ suggested an approach that work on machine learning techniques. They used app permissions as features that assist them to analyze the app relate to benign or malware class. Yuan et al.^12^ proposed “Droid-Sec” a machine learning model which uses more than 200 plus features for developing malware detection model. The proposed model obtained an detection rate of 96% with a very limited dataset. Yerima et al.^13^ suggested a novel framework for mitigating the effect of Android malware. They implemented a Bayesian classification for detecting unknown malware from Android-based devices. Liang et al.^14^ proposed a permission combination based method for detecting malware from Android devices. They implemented rule sets selection approach on extracted permission and applied K-map for identifying malware from Android devices. They used limited dataset for evaluating their proposed model.

Talha et al.^15^ proposed a permission-based Android malware system i.e., APK Auditor to identify app belong to malware or benign category. It worked on the principle of static analysis. Their implemented approach work on three components first is a signature database which helps to store extracted data about the Android app and helps in analyzing the results. Second, the component is an Android client that helps Android end-user to grant a request to an app and the third component is a central server that communicates between the signature database and end-user that help to manage the whole process. The experiment was performed on 8762 Android apps and achieved an accuracy of 88%. Canfora et al.^16^ proposed an Android malware detection approach, which worked on sequences of system calls. They build the model where prior knowledge of malware or benign apps not required, it automatically learns the association. The experiment was conducted on 20,000 Android apps and able to detect 97% malware-infected apps. Yerima et al.^17^ proposed an approach that used ensemble learning for malware detection. They build a model on the basis of static analysis and used a large repository of malware samples from a leading antivirus vendor. Their results revealed that they gained a detection accuracy varying from 97.3% to 99%.

SensDroid^18^, a framework that detects Android malware by analyzing intents and permissions to identify privacy risks and malicious behavior. It extracts features through reverse engineering, applies rule-based scoring, and classifies apps into high, medium, or low risk using sensitivity analysis. Experimental results on real datasets show high detection accuracy ( 98.65%), demonstrating that combining intents with permissions improves malware detection effectiveness. The paper^19^ introduces RNPDroid , a permission-based risk mitigation approach for Android malware detection using reverse engineering to analyze 165 permissions from 400 apps. Statistical analysis using ANOVA shows significant differences among risk factors, supported by a high F-value (517.3> 2.61). Additionally, a *t*-test confirms a significant distinction between medium and low risk levels, validating the effectiveness of the proposed method. PET-Droid^20^ is an unsupervised malware detection model that uses static analysis of features like permissions, intents, texts, and methods to distinguish malware from goodware. It leverages clustering (GMM) with feature selection and dimensionality reduction to detect even unknown malware without relying on labeled data. Experimental results show high effectiveness, achieving up to  96.84% accuracy, though it is sensitive to code obfuscation.

PRAZDroid^21^ is a permission-based Android risk assessment model that analyzes app permissions using rule mining and pattern analysis to classify apps into five risk levels. It extracts permissions from APK files, applies algorithms like JRip and PrefixSpan, and builds rules to predict whether an app is benign or malicious. The model achieves high performance ( 98.07% accuracy) and helps users understand security risks before installing apps. In prior work^22^, Android applications are described as comprising core components such as activities, services, broadcast receivers, and content providers, while highlighting the rapid growth of Android malware and the limitations of traditional antivirus-based defenses in handling its scale and diversity. With the emergence of the Web of Things (WoT), Android has become a key enabling platform, further intensifying the need for advanced malware analysis techniques. To address this, the study emphasizes behavioral analysis of applications and proposes an automated feature extraction tool that generates structured datasets (e.g., CSV format) to facilitate efficient malware detection and analysis.

Sun et al.^23^ proposed “SIGPID”, which analyzes 135 Android permissions. They further applied a 3-level pruning process, including permission ranking based on negative rate, support-based permission ranking, and permission mining using association rules, to identify the most relevant permissions for developing the model. By selecting 22 permissions they achieved an accuracy of 90% by implementing an SVM classifier. Chen et al.^24^ proposed “StormDroid”, a malware detection technique based on machine learning algorithms. It supports a large-scale malware detection process, providing a scalable and efficient method that dynamically and statically observes Android app behavior. They performed experiments on 8,000 Android apps, and it improves the accuracy by 10% related to state-of-art antivirus systems. “HinDroid” proposed by Hou et al.^25^, which work on the principle of combination of API calls with their relationship between them. They build structured heterogeneous networks (HIN) which help to prevent user’s privacy. McLaughlin et al.^26^ proposed a technique for malware detection which use Deep Convolutional Neural Network (CNN) as a machine learning algorithm. They proposed an approach that automatically learned from the network of the raw opcode sequence. Their accuracy depends upon the size of the dataset; with small dataset achieved higher accuracy and vice-versa. Milosevic et al.^27^ presented two machine learning approaches for malware detection. One approach is based on permission and the other is based on source code analysis. Their proposed approaches achieved the F-score of 95.1% and 89%. The authors^28^ introduced “MAPAS”, an Android malware detection system that utilizes deep learning (CNN) and API call graph analysis to efficiently identify malware while reducing computational resource demands. Unlike traditional deep learning-based approaches that require significant computing power, MAPAS employs a lightweight classifier, making it more practical for real-world deployment.

Mahindru and Singh^3^ evaluated various machine learning algorithms using a dataset of over 11,000 Android apps. They identified 172 unique permissions from these apps to distinguish between malware and benign applications. Zhu et al.^29^ introduced a highly efficient malware detection method that leverages features such as permissions, monitored system events, sensitive APIs, and permission rates. By applying an ensemble Random Forest algorithm to a dataset of 2,130 Android apps, they achieved an detection rate of 88.26%. Shang et al.^30^ introduced an enhanced version of the Naïve Bayes classification algorithm tailored for detecting Android malware. Their improvements to the algorithm resulted in an accuracy of 97.59%. Wang et al.^31^ developed a serial Convolutional Neural Network (CNN-S) to improve the accuracy of malware detection models. Table 1, represents the approaches, machine learning algorithms, and features used by various authors in the past.

The authors^32^ introduce a novel approach called “IntDroid”, which integrates graph-based analysis with social-network-based analysis to enhance both accuracy and scalability in Android malware detection. While traditional graph-based methods offer high accuracy but struggle with scalability, social-network-based approaches handle large-scale detection efficiently but risk misclassifying benign apps that exhibit malware-like behavior. IntDroid overcomes these limitations, achieving an F-measure of 97.1% and a true-positive rate (TPR) of 99.1% on a dataset comprising 3,988 benign apps and 4,265 malware samples. Gao et al.^33^ introduced GDroid, an approach for Android malware detection and familial classification based on a Graph Convolutional Network (GCN). The core innovation lies in modeling Android apps and APIs as a heterogeneous graph, converting malware classification into a node classification task. GDroid achieves a 98.99% malware detection rate with a false positive rate below 1% and demonstrates 97% accuracy in malware familial classification, outperforming existing baseline models. The authors^34^ introduce BMD (Bi-level Malware Detection), a novel approach to Android malware detection that formulates the problem as a Bi-Level Optimization Problem (BLOP). Unlike conventional methods, BMD leverages evolutionary algorithms to generate both detection rules and artificial malware patterns, enhancing its ability to identify new and unseen malware variants. Evaluated on a dataset of 3,000 Android apps (2,000 malware, 1,000 benign), BMD achieved 98.06% precision, 98.34% recall, and 98.18% accuracy, demonstrating its effectiveness in malware detection.

Authors proposed SETTI (A Self-supervised AdvErsarial Malware DeTection ArchiTecture in an IoT Environment)^35^, an adversarial self-supervised architecture that can learn from unlabeled IoT network traffic. The framework introduces three adversarial attack techniques–Self-MDS, GSelf-MDS, and ASelf-MDS–to simulate realistic attack scenarios and evaluate model robustness. Additionally, an adversarial self-supervised training mechanism is employed as a defense strategy to mitigate these attacks. Experimental results on IoT23 and NBIoT datasets demonstrate that adversarial attacks can significantly degrade detection accuracy, highlighting the importance of robust, self-supervised defense mechanisms in IoT malware detection systems.

Authors^36^ proposed two novel attack strategies: SLF (Stealthiness-based Label Flipping), which subtly alters labels to avoid detection, and WALF (Weight-based Adaptive Label Flipping), which targets samples based on model confidence. To defend against these threats, two density-based mechanisms–LOF and DBSCAN–are introduced to detect anomalous patterns. Experimental results on benchmark datasets (Drebin, Contagio, Genome) demonstrate that the proposed defenses significantly improve robustness, achieving up to 98.11% accuracy, outperforming existing methods like FedDefender.

DroidRL^37^ is a reinforcement learning-based feature selection framework designed for Android malware detection. Traditional feature selection methods often overlook feature relevance or incur high computational costs. DroidRL addresses these challenges by leveraging Deep Q-Networks (DQN) to efficiently identify an optimal feature subset, enhancing classification performance while significantly reducing computational overhead. Evaluated using the AndroZoo and Drebin datasets, DroidRL achieves a 97.78% reduction in computational overhead while maintaining high detection accuracy. Mahindru et al.^2^ introduce “PermDroid”, a machine learning-based Android malware detection framework that leverages an advanced feature selection approach to enhance both detection accuracy and efficiency. The framework emphasizes Android permissions analysis and integrates ensemble learning and neural network models to improve malware identification.

Previous research on Android malware detection faces several limitations, including high computational costs, reliance on limited datasets, and difficulty in detecting sophisticated malware. To overcome dataset constraints, this study collects over 500,000 Android apps, categorizing them into thirty distinct groups as outlined in Section 3. To mitigate computational overhead, six feature selection techniques are applied to extract key attributes such as API calls, app permissions, app ratings, and user download counts. These selected features serve as inputs for model training using semi-supervised machine learning algorithms.

Additionally, prior work^7^ does not specify the optimal number of labeled samples required for an effective malware detection model. This study fills this gap by training various machine learning algorithms with labeled datasets ranging from 5% to an optimal amount, assessing their performance using F-measure and accuracy as evaluation metrics.

### Research questions

To bridge these gaps, this paper investigates the following research questions:

**RQ1.** Which machine learning algorithm offers the highest effectiveness in detecting malware-infected apps in real-world environments?

The primary motivation for including this question in our study is to identify the most effective model for Android malware detection. In this research, 60 distinct models is developed by applying six different feature ranking techniques and ten machine learning algorithms–comprising five supervised and five semi-supervised approaches. The performance of these models is then evaluated and compared using F-measure and Accuracy as key metrics.

**RQ2.** Is the proposed malware detection framework efficient in identifying malware?

To answer this question, the performance of our proposed framework is compared with existing frameworks from the literature. Additionally, we evaluated our framework against various commercial antivirus scanners available in the market.

**RQ3.** Do the implemented feature ranking approaches enhance the ability to identify malicious apps compared to using all extracted features?

To answer this question, we compare models developed using features selected by various feature ranking approaches with models using the full set of extracted features. The performance of these models is evaluated using two metrics: F-measure and Accuracy.

**RQ4.** To assess the effectiveness of feature ranking approaches in detecting malware-infected apps in real-world scenarios.

The efficiency of machine learning models in malware detection depends on the features used for their development. This question seeks to compare models built using different feature ranking approaches, evaluating their effectiveness based on Accuracy and F-measure.

**RQ5.** Do feature ranking techniques influence the outcomes of semi-supervised machine learning approaches?

To answer this question, we trained various semi-supervised machine learning algorithms using features selected through different feature ranking approaches. The performance of the resulting models was then evaluated and compared using F-measure and Accuracy as key metrics.

**Table 1:** Android malware detection as discussed in the literature.

Author	Feature used	Machine learning algorithm used	Observations	Accuracy
Almin and Chatterjee^38^	Permissions	K-mean and NB	Unknown malware families are not identified	—
Sheen et al.^39^	Permissions and API call	J48, NB and SVM	Having high computation burden	97%
Andriatsimandefitra and Tong^40^	Information-flow	N/A	Dataset is too small	100%
Caviglione et al.^41^	Convert Channel	NN and DT	Unable to detect unknown malware families	75.7% with RBD Neural 92.2% with CBD neural network and 79.2% with RBD decision tree and 88.3% with RBD decision tree
Holland et al.^42^	API and Permissions	Pattern Matching	Dataset used for testing is small and outcome is not so good	85.7%
Shen et al.^43^	Topology	VF2 Algorithm Graphs	Detection rate is low	Averaged 89%
Rahman^44^	API	Markov Logic Network	Low detection rate	Averaged 84%
Martinelli et al.^45^	System Calls	SVM	High detection but consume many local resources	99.7%
Ng et al.^46^	System Calls	Dendritic cell Algorithm	Three different classifiers are used	94.1% and 98.4% for the NI and WI dataset (belong to system call)
Alam and Vuong^47^	Battery Permissions and CPU	Random Forest	Dataset is not collected on smartphone	99%
Amos et al.^48^	Battery Permissions and CPU	Random Forest, J48 and Logistic	Detection rate is not so good	Detection rates from 68.75% to 81.25% when applied on real-world data
Wei et al.^49^	Network Behavior	Naïve Bayes and Logistic	Better Performance	100%
Tong and Yan^50^	System call	Pattern matching	High Power consumption and Not Real	90%
Yang et al.^51^	Permissions	Pattern matching	Consumption of memory is very high and not able to protect privacy	—

**RQ6.** How many labeled instances are needed to achieve acceptable accuracy?

This question helps in identifying the optimal number of labeled dataset that is required to develop an effective malware detection model. For this, the model developed using 100% labeled dataset is compared with an optimal number of labeled dataset that is suggested by our proposed machine learning algorithm.

## Experimental dataset

### Collection of Android app package files (.apk)

To address the limitations of the dataset, this study includes over half a million Android application package files (.apk)(both benign and malware-infected used in this study), representing thirty different categories of Android apps. For this, 5,19,436 .apk files is collected from official Google play-store (https://play.google.com/store?hl=en), appchina (http://www.appchina.com/), hiapk (http://www.hiapk.com/), Android (http://andrdoid.d.cn/), mumayi (http://www.mumayi.com/), gfan (http://www.gfan.com/), pandaapp (http://android.pandaapp.com/) and slideme (http://slideme.org/). After performing data cleaning steps–including deduplication, removal of corrupted files, and filtering of incomplete metadata–the final dataset used for experimentation was reduced to a 4,75,000 unique apps. In this study, a three-step verification process is adopted. First, only applications from verified developers with no history of malicious behavior were considered. Second, all collected benign samples were scanned using multiple antivirus engines i.e., Microsoft Windows Defender (https://www.microsoft.com/en-in/windows/comprehensive-security) and VirusTotal (https://www.virustotal.com/), only those apps that were consistently flagged as benign (i.e., zero or negligible detections across engines) were retained. Third, we excluded newly uploaded or low-reputation applications to minimize the risk of including potentially undiscovered malware. To enhance the effectiveness and efficiency of the Android malware detection framework, this study also incorporates paid apps into its analysis.

**Table 2:** Classification of .apk files consider in this study.

ID	Category	Normal	Trojan	Backdoor	Worms	Botnet	Spyware
DS1	Arcade and Action (AA)	13291	1240	200	104	330	500
DS2	Books and Reference (BR)	14235	1000	1250	46	180	130
DS3	Brain and Puzzle (BP)	12928	1200	14	18	0	0
DS4	Business (BU)	18308	1420	25	10	22	52
DS5	Cards and Casino (CC)	10886	300	115	51	24	200
DS6	Casual (CA)	13010	110	19	36	10	14
DS7	Comics (CO)	14667	62	15	30	3	5
DS8	Communication (COM)	15414	200	50	30	23	3
DS9	Education (ED)	18744	0	68	0	0	8
DS10	Entertainment (EN)	11222	50	500	40	20	42
DS11	Finance (FI)	12999	40	30	89	75	92
DS12	Health and Fitness (HF)	16551	1900	65	65	120	148
DS13	Libraries and Demo (LD)	15655	60	10	90	16	500
DS14	Lifestyle (LS)	17650	135	220	103	192	190
DS15	Media and Video (MV)	18019	9	13	152	41	70
DS16	Medical (ME)	15000	10	1500	10	26	25
DS17	Music and Audio (MA)	28057	55	15	60	10	15
DS18	News and Magazines (NM)	19164	200	500	500	20	32
DS19	Personalization (PE)	15334	1400	12	40	30	22
DS20	Photography (PH)	19033	120	1000	780	206	400
DS21	Productivity (PR)	19950	20	46	10	30	62
DS22	Racing (RA)	17766	10	0	310	0	180
DS23	Shopping (SH)	13673	20	0	22	5	5
DS24	Social (SO)	27159	15	0	31	50	15
DS25	Sports (SP)	23669	20	40	20	35	112
DS26	Sports Games (SG)	12889	200	45	245	550	198
DS27	Tools (TO)	14346	220	400	450	575	563
DS28	Transportation (TR)	12300	20	30	20	0	20
DS29	Travel and Local (TL)	22180	40	32	10	48	150
DS30	Weather (WR)	10841	12	12	60	0	75

Malware families are identified by anti-virus scanners.

A total of 47,099 malware samples were collected from three distinct repositories: the Botnet dataset^52^, the Malgenome project^53^, and the AndroMalShare dataset (http://sanddroid.xjtu.edu.cn:8080/). Kadir et al.^52^ introduced a dataset containing 1,929 Android botnet samples, which represent 14 different botnet families. The Android Malware Genome project^53^ offers a collection of 1,200 malware samples, encompassing a broad range of existing Android malware families. Additionally, approximately 17,871 samples from AndroMalShare were collected, including their package names. After removing duplicate packages, our final dataset consists of 25,000 unique malware samples. Both malware and benign apps were gathered from these sources up to January 2024. To make the model more effective and efficient in this study, malware-infected apps collected from different repositories belongs to 141 malware families mentioned in Appendix B. The considered malware families refer to fine-grained groupings of malware samples based on shared code structure, origin, and behavioral characteristics (e.g., specific families such as DroidKungFu, FakeInstaller, etc.). On the other hand, the labels presented in the dataset Table 2–such as Trojan, worm, botnet, adware, and spyware–represent coarse-grained malware types, which categorize malware based on their primary functionality or attack behavior. The relationship between these two is hierarchical: each malware family is mapped to a corresponding high-level category based on its dominant behavior. For instance, multiple families exhibiting credential theft or disguised malicious functionality are grouped under the “Trojan” category, while others demonstrating self-propagation behavior are classified as “worm”, and those involved in coordinated network control are labeled as “botnet”. Table 2 details the categories of the Android apps and the number of samples collected for this study. The datasets from these repositories are publicly available for academic and research purposes (Mahindru, Arvind (2025), “Android Benign and Malware Dataset2”, Mendeley Data, V2, doi: 10.17632/rvjptkrc34.2).

### Extraction and formulation of features dataset

To extract features, a series of distinct steps were followed as outlined in the research paper^2,22^. By repeating the same process, features were extracted from over half a million distinct Android apps. The extracted features were saved in a .csv file, which is publicly available for academicians and researchers (http://dx.doi.org/10.17632/b4mxg7ydb7.3).

In previous studies^18,21,38,42,48,50^, researchers have typically used limited feature sets to develop malware detection models. To address this gap, this study incorporates a comprehensive set of features, including 310 API calls, 1,532 permissions, app ratings, and the number of app downloads, to enhance the effectiveness of malware detection.

The main reason for considering app ratings, and the number of app downloads is that API calls and permissions capture the intrinsic behavioral characteristics of applications, metadata features like ratings and download counts provide complementary contextual and distributional signals from real-world app ecosystems. Moreover, benign apps tend to have higher download counts, more stable usage patterns, and relatively consistent user ratings, whereas malicious apps often exhibit lower download volumes, short lifespans, or irregular rating patterns. These differences can serve as indirect yet informative indicators for distinguishing benign from malicious apps.

In addition to that, these features are not used in isolation but are integrated with core security features to enhance the model’s discriminative capability. Our empirical analysis also shows that incorporating such metadata improves overall detection performance, particularly in borderline cases where behavioral features alone may be insufficient.

**Table 3:** Formulation of feature dataset.

Set Number	Description	Set number	Description
FS1	Info. associate with HARDWARE_CONTROLS	FS2	Info. associate with API calls
FS3	Info. associate with PHONE_CALLS	FS4	Info. associate with SYSTEM_SETTINGS
FS5	Info. associate with ACCOUNT_SETTINGS	FS6	Info. associate with BROWSER_INFORMATION
FS7	Info. associate with PHONE_STATE and PHONE_CONNECTION	FS8	Info. associate with LOG_FILE
FS9	Info. associate with AUDIO and VIDEO	FS10	Info. associate with WIDGET
FS11	Info. associate with BUNDLE	FS12	Info. associate with SYNCHRONIZATION_DATA
FS13	Info. associate with LOCATION_INFORMATION	FS14	Info. associate with CONTACT_INFORMATION
FS15	Info. associate with CALENDAR_INFORMATION	FS16	Info. associate with FILE_INFORMATION
FS17	Info. associate with IMAGE	FS18	Info. associate with READ
FS19	Info. associate with SMS_MMS	FS20	Info. associate with READ_AND_WRITE
FS21	Info. associate with DATABASE_INFORMATION	FS22	Info. associate with ACCESS_ACTION
FS23	Info. associate with UNIQUE_IDENTIFIER	FS24	Info. associate with STORAGE_FILE
FS25	Info. associate with YOUR_ACCOUNTS	FS26	Info. associate with NETWORK_INFORMATION
FS27	Info. associate with SERVICES_THAT_COST_YOU_MONEY and BLUETOOTH_INFORMATION	FS28	Info. associate with SYSTEM_TOOLS
FS29	Info. associate with Default group	FS30	Contain info. associate with rating and downloads

The formulation of the feature dataset is based on behavioral characteristics outlined in the literature^2^.

To normalize the data, the Min-Max normalization method^2^ was applied. Each collected app is represented as a 1,842-dimensional Boolean vector, where “1” indicates the presence of a permission, and “0” indicates its absence. Different Android apps often request similar permissions for installation and execution, as many core functionalities rely on common permission sets. Permissions are categorized by Google (https://developer.android.com/guide/topics/permissions/overview) into “dangerous” or “normal” classes. Several frameworks have been proposed for malware detection on smartphones^3,10,54^. In this study, the collected features is divided into thirty distinct datasets, each used as input for developing malware detection models. Table 3 provides basic descriptions of the feature sets considered in this study.

## Implemented feature selection approaches

In previous studies^9,13,55–58^, researchers have implemented various feature ranking approaches to improve malware detection accuracy. In this paper, six different feature ranking techniques is employed to identify the most effective features for constructing a malware detection model. Each approach ranks features based on specific criteria, and the most relevant features are selected for model development to enhance detection performance.

Feature ranking approaches operate independently, without relying on any training algorithm. The ranking is determined based on feature scores, assessing their relevance for malware detection. As highlighted in previous studies^9,13,55–58^, various feature ranking methods have been employed to prioritize features for model development.

The main reason for selecting six feature ranking methods is to provide a diverse representation of filter-based and statistical approaches, ensuring low computational complexity and scalability for high-dimensional feature spaces such as ours (1,842 features). These methods are widely adopted in malware detection literature and allow efficient ranking without requiring repeated model training, which is particularly important given the size of our dataset. The other commonly used methods, such as LASSO, mutual information, and embedded techniques, are also effective for feature selection. But, these approaches typically involve higher computational overhead or require iterative model training, which can be less efficient when applied to large-scale datasets and other limitations too^59–63^.

The primary focus of this study is to evaluate lightweight and generalizable ranking strategies that can be easily integrated into different classifiers without tight coupling to a specific model.

In this research, six distinct feature ranking approaches are implemented to systematically rank features based on their importance. Table 4 provides a detailed overview of the feature ranking approaches used in this study.

**Table 4:** Implemented feature ranking approaches.

Name of the approach	Description
Gain-ratio feature selection^64^	It operates based on the gain-ratio principle, which measures the relevance of each feature concerning the class to which a particular app belongs (malware or benign)
	Gain-ratio “Z” of feature is measured as: $\text{ Gain-Ratio }=\frac{Gain(Z)}{SplitInfo_{Z(A)}},$ in this Gain *(Z)=I(A)-E(Z)* here, *X* represents the total number of instances with *n* different classes, while *A* denotes the set of extracted features
Chi-Square feature selection approach^65^	This feature selection approach is used to evaluate the independence between two cases. In our research, feature ranking is determined based on the statistical significance of each feature, which depends on its relevance to the class (malware & or benign)
	A higher measured value indicates the reduction of outliers, suggesting that the selected features have greater relevance in identifying malware-infected apps more effectively
Information-gain feature selection approach	In Info-gain, features are picked on its relation with respect to the class to which it belongs^64^
OneR feature selection approach^64^	To rank specific features, this approach employs a classification mechanism
	Valuable features are identified as consistent ones, and the set of values is divided into distinct intervals using a straightforward partitioning method. In this study, we select only features that demonstrate higher classification rates for improved malware detection accuracy
Principal Component Analysis (PCA)^66^	Principal Component Analysis (PCA) is applied to reduce the dimensionality of our collected dataset PCA transforms high-dimensional feature space into a lower-dimensional representation while preserving essential patterns. The reduced feature set retains significant information, enhancing the ability to detect malware-infected apps efficiently
Logistic regression analysis ^67^	In this technique, Univariate Logistic Regression (ULR) analysis is used to determine the significance level of each feature set, helping to identify the most relevant features for malware detection

## Machine learning techniques

This paper presents a machine learning algorithm for Android malware detection, inspired by biological immune systems and built on the YATSI framework, specifically designed to function with a limited labeled dataset. The Artificial Immune System (AIS) is an emerging computing paradigm modeled after biological immune responses. Its growing popularity has led to the development of new classification and clustering algorithms, such as Artificial Immune Recognition System (AIRS). The AIRS algorithm, introduced by Watkins^68^, has been successfully applied to various classification problems.

The proposed algorithm in this study incorporates the following key features:

- Stability of parameter: No need to optimize the algorithm’s parameters extensively,
- Generalization: It is not necessary to use all data points for effective generalization,
- Performance: Exhibits high performance across various datasets,
- Self-regulatory: Does not require pre-determined topology before training.

**Fig. 2:**
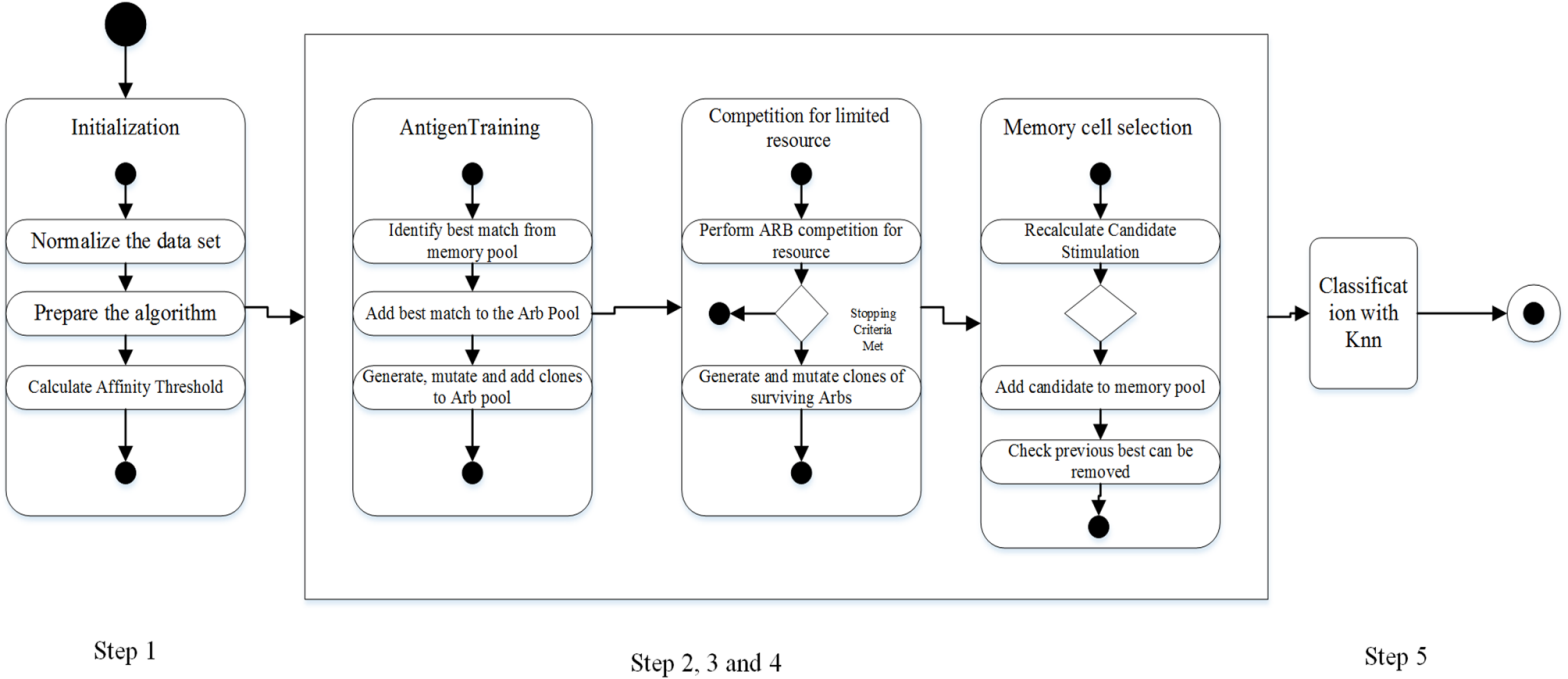
Activity diagram of AIRS algorithm.

The novelty of the proposed algorithm is described as the original YATSI algorithm is a semi-supervised learning method that relies on label propagation over a similarity graph constructed from labeled and unlabeled instances. In contrast, YATSIDroid introduces several important modifications tailored specifically for Android malware detection. First, instead of directly applying YATSI on raw feature space, integration of AIRS (Artificial Immune Recognition System) is considered as a preprocessing and prototype selection mechanism. AIRS is used to generate a reduced and more representative set of memory cells, which helps in handling high-dimensional and noisy Android feature data (e.g., permissions and API calls). This significantly improves scalability and reduces computational complexity compared to standard YATSI.

Second, the similarity graph used in YATSIDroid is constructed over these AIRS-generated memory cells rather than the entire dataset, which enhances the quality of neighborhood relationships and reduces redundancy. Third, we adapt the weighting and neighborhood selection strategy to better capture the characteristics of malware data, where class imbalance and feature sparsity are common challenges.

Additionally, YATSIDroid incorporates domain-specific feature engineering and normalization steps that are not part of the original YATSI framework, enabling more effective learning in the Android malware context. These modifications collectively improve classification performance, robustness to noise, and generalization to unseen malware samples.

Therefore, the novelty of YATSIDroid lies not merely in combining AIRS and YATSI, but in redesigning the learning pipeline to leverage immune-inspired prototype selection with graph-based semi-supervised learning, specifically optimized for Android malware detection.

In addition to that, in the proposed AIRS-based framework, several mechanisms are incorporated to prevent the propagation of errors from incorrectly pseudo-labeled samples:

1. **Confidence-based pseudo-labeling:** Unlabeled samples are assigned pseudo-labels only when the model prediction exceeds a predefined confidence threshold. Samples with low confidence are excluded from the training process, thereby reducing the risk of introducing noisy labels.
2. **Affinity-based filtering (AIRS principle):** The AIRS model evaluates each sample using an affinity measure. Only those samples that exhibit strong similarity with existing memory cells are retained. Ambiguous or low-affinity samples are discarded, ensuring that unreliable instances do not influence the learning process.
3. **Memory cell competition and pruning:** AIRS employs a clonal selection and resource competition mechanism, where only the most representative and high-quality memory cells survive. This inherently suppresses the impact of incorrectly labeled samples over time.
4. ** Iterative refinement strategy:** The model incorporates pseudo-labeled samples gradually across iterations, allowing the system to refine its predictions. This controlled inclusion minimizes the accumulation of labeling errors and enhances robustness.
5. **Noise-tolerant design:** Due to its immune-inspired nature, AIRS is inherently robust to noise, as it reinforces consistent patterns while suppressing outliers and uncertain samples.

### Immune principle employed in our proposed Algorithm

In the literature^69^, the author conducted an experiment using AIRS with YATSI fixed in the first stage for software fault prediction, achieving notable results. In this study, we also employed the Immune-based YATSI, a specialized version of the YATSI algorithm, for Android malware detection, focusing on two classes: benign and malware. The AIRS algorithm comprises five steps: (a) Initialization, (b) Antigen Training, (c) Competition for Limited Resources, (d) Memory Cell Selection, and (e) Classification^70^. Steps 1 and 5 are performed once, while steps 2, 3, and 4 are repeated for each sample in the dataset, which we refer to as an antigen in this research. Figure 2 illustrates the activity diagram of the AIRS algorithm, and the steps followed are detailed below:

1. *Initialization:*In the first step, the dataset is prepared for training. The affinity Threshold variable (the average affinity value among all the antigens in the training set) is constructed.
2. *Antigen Training:* Antigens are submitted to the memory cells one by one. Cells are recognized in the memory pool and also stimulated. A cell also got a stimulation value in the memory pool. The cell which is having a maximum stimulation value is selected as the best match memory cell and used for the affinity maturation process. Further, the selected cell is cloned and mutated. Selected clones are included in the Artificial Recognition Ball (ARB) pool. The number of clones is calculated using the equation defined below: numClones = stim*clonalRate*hypermutationRate where *stim = 1 - affinity* and *hyperMutationRate*and *ClonalRate* are user-defined parameters.
3. *Competition for limited resource:* In the third step of the AIRS algorithm, competition starts when mutated clones are introduced into the ARB pool. The antigen stimulates the ARB pool, and resources are distributed according to the stimulation values. ARBs that fail to acquire enough resources are eliminated. If the stopping criteria are met, the process terminates. Otherwise, new mutated clones of the remaining ARBs are produced, and the cycle repeats until the stopping criteria are fulfilled.
4. *Memory cell selection:* Once the stopping criteria from the previous step are satisfied, the ARB with the highest stimulation score is chosen as the candidate memory cell. If its stimulation value exceeds that of the original best-matching pool, it is added to the memory pool. The learning process then concludes before advancing to the next step.
5. *Classification:* In the final step, the memory cell pool is used for cross-validation or testing new data. In our study, we employed the K-nearest neighbor (KNN) approach as the classification algorithm.

Additionally, Table 5 presents the mapping between AIRS and the immune system. A more comprehensive analysis of the immune system and its connection to computer science and engineering can be found in^71,72^.

**Table 5:** Correlation Between the Immune System and AIRS.

Immune system	AIRS
Antigens	Training data. This is the same in representation as an antibody
Affinity	A metric for assessing similarity between two antigens. In our study, this
	value ranges from 0 to 1 (inclusive) and is determined
	using the Euclidean distance between the two objects
B Cell	In this study, B cell is referred to as Artificial Recognition Ball (ARB).
Antibody	A feature vector paired with its corresponding output or class.
Immune memory	Memory set of mutated ARBs. It is also known as B-cell. ARB consists
	of an antibody, number of the resources held by the cell
	and the current stimulation value of the cell
Recognition ball	It is a combination of vector class (i.e., benign or malware) and feature vector.
Metadynamics	Ongoing elimination and generation of memory cells and ARBs
Affinity maturation	Removal of the least stimulated ARBs
Shape-space	Possible and type value of the data vector
Clonal expansion	Replication of ARBs that closely match antigens

Our proposed immune-based YATSI algorithm with AIRS has the following characteristics i.e., precision (means steps are precisely stated), uniqueness (means results of each step are uniquely defined and only depend on the input and result of the proceeding steps), finiteness, and generality. Our proposed immune-based YATSI algorithm is divided into two stages.

- In the first stage, the AIRS algorithm is used to label the unlabeled data after constructing the model with the initial labeled data. Once labeling is complete, weights are assigned to both the newly labeled and existing labeled data. Higher weights are given to the existing labeled data to reflect its greater confidence level.
- At the second stage, weights of neighbor are computed by using *k*-nearest neighbors (KNN) of each data point and assigned to the class to which it belongs. To identify the class to which the unlabeled data point belongs, it depends upon the higher value of the weight assigned to the data point (In our study, we consider the value of adjustment factor of weight to be 1 (i.e., the value of *K*=1).).

***Algorithm***      ***Proposed immune-based YATSI algorithm with AIRS for Android Malware Detection (YATSIDroid)***

   Input:      Labeled data $D_{labeled}$, unlabeled data $D_{unlabeled}$, nearest neighbour number *k*, $N = |D_{labeled}|$,

              $M = |D_{unlabeled}|,$ unlabeled data point $d_{unlabeled}$

  Step 1: a Implement AIRS machine learning algorithm to developed the model $M_{1abeled}$ using $D_{1abeled}$

          b:    Implement $M_{1abeled}$ to pre-label data points of $D_{unlabeled}$

          c:    Allocate 1.0 weight to data points of $D_{1abeled}$ and *N=M* weight to points of $D_{unlabeled}$

          d:    Integrate $D_{1abeled}$ and $D_{unlabeled}$ to produce *D*

  Step 2:    For each data point $d_{unlabeled}$ inside $D_{unlabeled}$

          a:    Examine for the *k* -nearest neighbours(KNN) to the $d_{unlabeled}$

          b:    Sum the weights of the fault-prone *k* -nearest neighbours $(W_{fp})$

          c:    Sum the weights of the not fault-prone *k* nearest neighbours $(W_{nfp})$

          d:    Detect the label of the $d_{unlabeled}$ with the largest weight $(W_{fp} \text{ or } W_{nfp})$

  Step 3:    Repeat the step 1 and 2 till the prediction of the labeled data $D_{labeled}$ is not fixed (In our study we fixed two parameters for checking the performance of developed model i.e., accuracy and F-measure and implement it with for loop to verify if the detection rate of developed model is greater than 90% then only labeled dataset is acceptable).

Moreover, pseudo-labeling is strictly regulated through confidence-based filtering, where only high-confidence predictions are considered for inclusion in the training set, while uncertain samples are discarded. Additionally, the AIRS-based component introduces affinity-driven validation and memory selection, ensuring that only samples consistent with learned patterns are retained. The model further adopts an iterative refinement strategy, where pseudo-labeled data are incorporated gradually, reducing the risk of accumulating errors over time.

These mechanisms collectively help limit the influence of noisy labels and prevent bias amplification, thereby maintaining the robustness and generalization capability of the proposed model.

## Evaluation methods for the proposed framework

To assess the effectiveness of our proposed framework in detecting malware from real-world applications, we evaluate it using the following three approaches:

a. **Comparison with Existing Frameworks and Approaches:** In this study, we further validate our proposed framework by comparing it with existing frameworks and approaches from the literature, emphasizing two key performance metrics: Accuracy and F-measure.

b. **Comparison of the Proposed Framework with Various Commercial Antivirus Scanners:** In this research paper, our proposed framework is evaluated by comparing it with various commercial antivirus scanners available in the market for Android malware detection.

c. **Detection of known and unknown malware families:** In this study, the ability of our proposed framework is evaluated to detect both known and unknown malware families effectively.

## Performance parameters considered in this research paper

In this section of the paper, the key performance parameters used to evaluate our proposed malware detection model is defined. These parameters are calculated using the confusion matrix, which provides information on detected versus actual classifications from the detection models. Table 6 presents the confusion matrix for the malware detection model. In this study, two performance parameters–F-Measure^3^ and Accuracy^2^ are used to evaluate the performance of malware detection approaches. These parameters are defined as follows:

**Table 6:** Confusion matrix for classifying an Android app as either benign or malware. (*.apk*)

	Benign	Malware
Benign	Benign->Benign (TP)	Benign->Malware (FP)
Malware	Malware->Benign (FN)	Malware->Malware (TN)

**Fig. 3:**
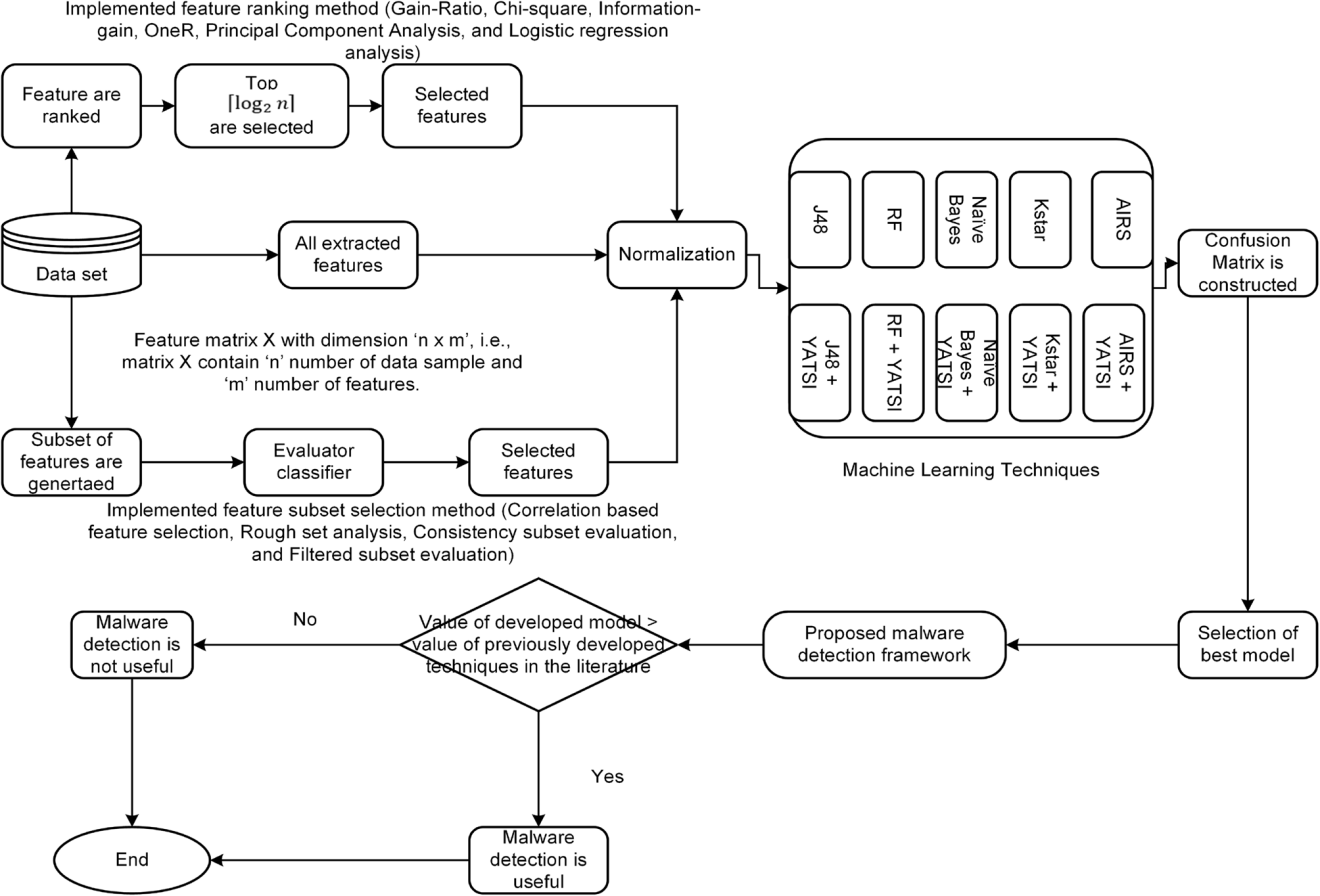
Framework of YATSIDroid.

- True Negative (TN) : A true negative occurs when the model accurately identifies an instance of the negative class.
- True Positives (TP): A true positive occurs when the model accurately identifies an instance of the positive class.
- False Negative (FN): A false negative occurs when the model incorrectly identifies a positive instance as negative.
- False Positive (FP): A false positive occurs when the model incorrectly classifies a negative instance as belonging to the positive class.
- Recall: Recall measures the proportion of actual positive instances that are correctly predicted by the model out of all the positive instances in the dataset.
  1 $$\begin{aligned} Recall =\frac{a}{a + c},\end{aligned}$$
  where $$a= N_{Malware\rightarrow Malware},$$ $$b= N_{Benign\rightarrow Malware},$$ $$c= N_{Malware\rightarrow Benign}$$
- Precision: Precision measures the count of positive class predictions that actually be a part of the positive class.
  2 $$\begin{aligned} Precision =\frac{a}{a + b}.\end{aligned}$$

**Accuracy:** Accuracy is defined as:

3 $$\begin{aligned} Accuracy=\frac{a+d}{N_{classes}},\end{aligned}$$

where $${N_{classes} = a+b+c+d}$$, $$d= N_{Benign\rightarrow Benign}$$

**F-measure:** F-measure is defined as:

4 $$\begin{aligned} F-measure=&\frac{2*Recall*Precision}{Recall+Precision}\nonumber \\&=\frac{2*a}{2*a+b+c} \end{aligned}$$

## Experimental setup

In this section of the paper, we outline the experimental setup used to develop and validate Android malware detection models. We apply ten different machine learning algorithms to 30 distinct categories of Android apps, as detailed in Table 2. The dataset includes both benign and malicious apps, with sufficient representation for our analysis. Figure 3 presents the framework of our proposed malware detection system, referred to as “YATSIDroid”.

Initially, feature ranking methods are applied to the collected feature dataset. Next, Min-max normalization is used to standardize the data. Following this, the most significant features–selected through various ranking methods is trained by using different machine learning techniques. In the fourth step, a confusion matrix is constructed and evaluate the performance of our models using two key metrics: F-measure and Accuracy. The performance of the developed malware detection models are compared to determine the most effective one. Finally, we assess the performance of our proposed malware detection model against existing techniques in the literature and various antivirus scanners. If our model outperforms the existing techniques, it is considered effective; otherwise, if it does not show improved efficiency, it is deemed less useful.

The selection process for identifying a subset of features to develop an Android malware detection model includes the following steps:

- Feature Selection Approaches: Various feature selection methods are implemented on features extracted from thirty distinct Android apps.
- Feature Subsets: This resulted in a total of 2,100 distinct detection models. This number is derived from the combination of 6 feature ranking approaches plus 1 approach that uses all extracted features, applied to 30 different datasets (each representing a subset of features after applying the feature selection methods), and tested with 10 different machine learning techniques.

Step by step detail of our proposed framework is mentioned below:

1. In this research paper, significant features are collected by applying six different feature ranking approaches to a dataset consisting of thirty distinct sets of collected features.
2. In this study, features identified through feature ranking techniques are utilized to train various machine learning classifiers. We employ a 20-fold cross-validation method to evaluate these models. Cross-validation operates on the principle of splitting the data into two distinct subsets^73^: one for training the model and the other for validating its performance^73^. Specifically, the data is partitioned into *K*-equally sized segments. During each iteration, K-1 segments are used for training, while the remaining segment is reserved for testing. This process ensures that every part of the dataset is used for both training and testing. In this paper, 20-fold cross-validation is applied, dividing the dataset into 20 segments. Further, the performance of the developed Android malware detection models is compared by using two metrics: F-measure and Accuracy.
3. The proposed framework, YATSIDroid, which is built using the aforementioned steps, is evaluated against existing frameworks and methods found in the literature. This comparison aims to assess the effectiveness and usefulness of the developed malware detection model.

## Experimental results

In this work, the relationship between malware detection and different feature sets at the level of class is submitted. Two distinct performance parameters i.e., Accuracy and F-measure that are used to compare the performance of Android malware detection models that are developed by using 10 different machine learning algorithms. To represent the experimental outcomes we used the abbreviations as given in Table 7 corresponding to their actual names.

**Table 7:** Name convention used in this research paper.

Abbreviation	Corresponding name
DS	Dataset
Y-J48	YATSI + J48
Y-RF	YATSI + RF
Y-NB	YATSI + Naïve Bayes
Y-Kstar	YATSI + Kstar
Y-AIRS	YATSI + AIRS
NB	Naïve Bayes
RF	Random Forest
AIRS	Artificial Immune Recognition
	System
AF	All Extracted features
DS	Dataset
FR1	Chi Square feature selection
	approach
FR2	Gain Ratio feature selection
	approach
FR3	OneR feature selection
	approach
FR4	Information Gain feature
	selection approach
FR5	Logistic regression analysis
	(ULR) feature selection approach
FR6	Principal Component Analysis
	(PCA) feature selection approach

###  Results on the basis of implemented feature ranking approaches

**Fig. 4:**
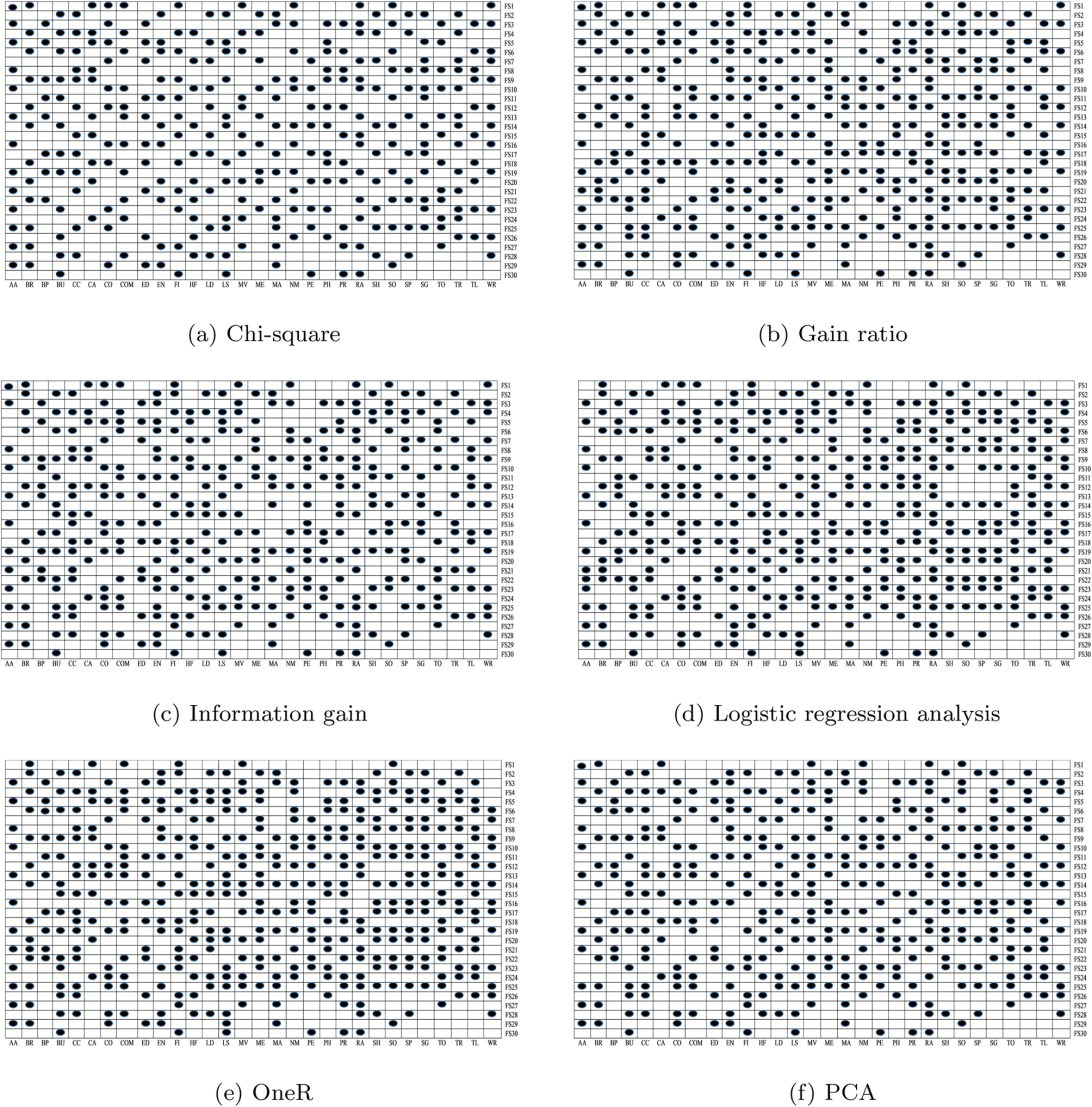
Feature ranking approaches.

Six different feature ranking approaches: Principal Component Analysis (PCA), Chi-Square feature selection approach, OneR feature selection approach, Gain Ratio feature selection approach, Logistic regression analysis, and Information Gain feature selection approach are implemented on different feature dataset. Each approach utilized different performance parameters to rank the features. In addition, the top $\lceil \log _2 a \rceil$ feature sets from “*a*” number of features are being evaluated to develop a model for detecting malware. For the first four feature ranking approaches (Gain Ratio feature selection approach, Chi-Square feature selection approach, OneR feature selection approach, and Information Gain feature selection approach), top $\lceil \log _2 a \rceil$ are chosen as a subset of features, where *a* is the number of features in the original dataset (for this work *a*=20). Nevertheless, in the case of ULR, features with a positive regression coefficient and a p-value below 0.05 are selected. For PCA, only features with an eigenvalue greater than 1 are chosen. The selected features using the implemented feature ranking approaches are presented in Fig. 4.

###  Implemented machine leaning algorithms

Seven different feature subsets are used as input to develop a malware detection model–six derived from feature selection approaches and one consisting of the complete set of extracted features. The experiment was conducted on a system with a Core i9 processor, 16 GB RAM, and a 1 TB hard disk, using the MATLAB environment. The effectiveness of each detection model is evaluated based on two performance metrics: Accuracy and F-measure.

**Fig. 5:**
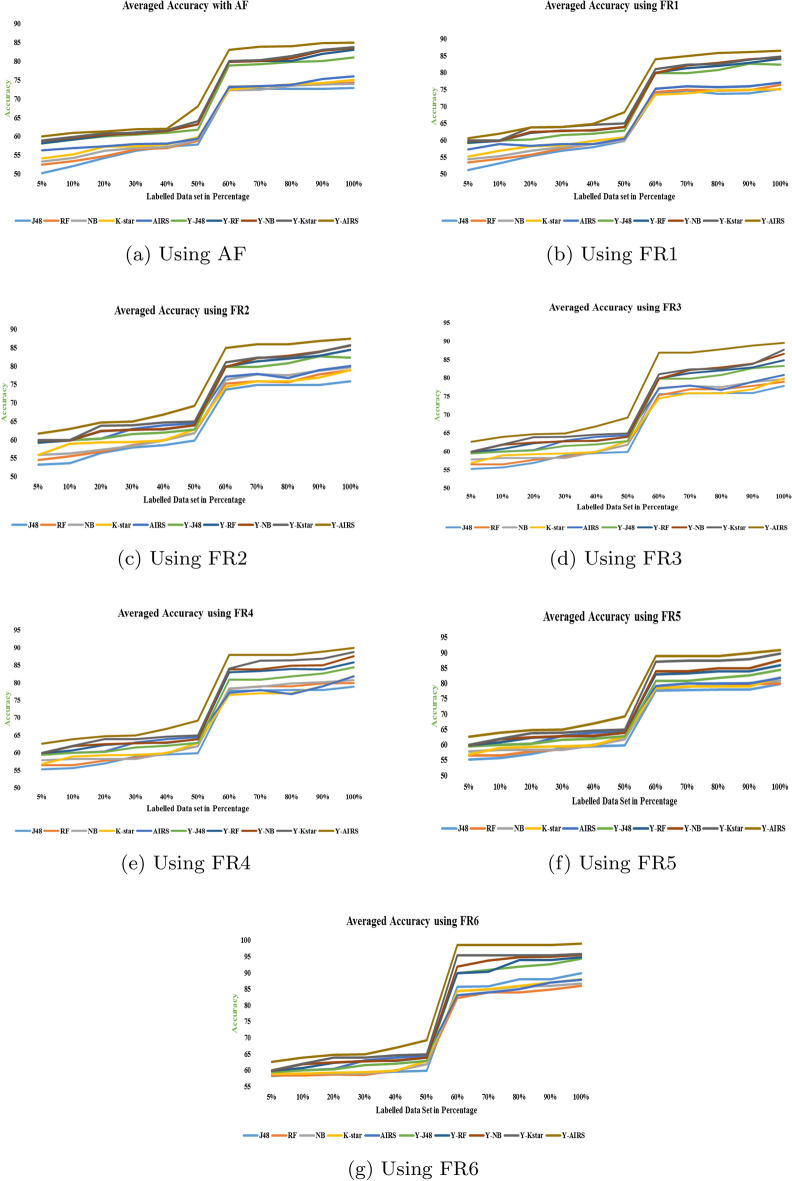
Averaged measured accuracy using 5%, 10%, ... 100% labeled dataset.

**Fig. 6:**
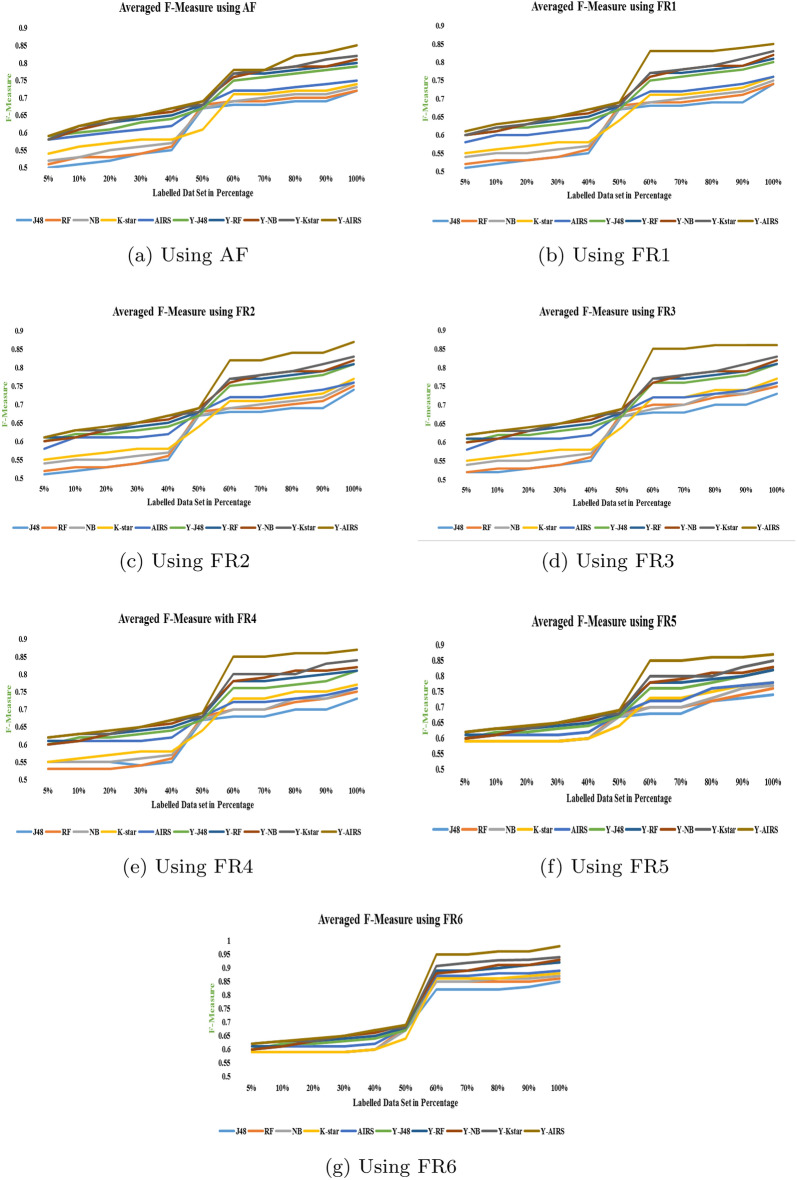
Averaged measured F-measure using 5%, 10%, ... 100% labeled dataset.

Figures 5 and 6 (F-Measure and Accuracy using 5%, 10%, ... 100% are presented in the appendix from Tables 12, 13, 14, 15, 16, 17, 18, 19, 20, 21, 22, 23, 24, 25, 26, 27, 28, 29, 30, 31, 32, 33, 34, 35, 36, 37 and 38. Additionally, we calculate F-measure and accuracy with an optimal number of labeled data points.) illustrate the performance values obtained for different datasets using J48, RF, NB, K-Star, AIRS, Y-J48, Y-RF, Y-NB, Y-K-Star, and Y-AIRS.

To determine the optimal number of labeled data points required to build the model, we trained our machine learning algorithms with increments of 5%, 10%, 20%, up to the optimal labeled dataset size (In this study, we also cross-verified the detection rate of our proposed algorithm using the optimal labeled dataset against a fully labeled (100%) dataset.).

Based on Figs. 5 and 6, the following conclusions can be drawn:

- Models built using selected features obtained through feature selection approaches as input can identify malware-infected apps more effectively than models developed using all extracted features from Android apps.
- The model constructed using features selected by PCA (i.e., FR6) as input achieved a higher detection rate compared to other malware detection models built using different feature selection approaches.
- The model built using YATSI + AIRS along with features selected by FR6 as input achieved a higher detection rate compared to other malware detection models developed using different feature ranking approaches.
- There is a drastic change in accuracy and F-measure, when we developed the model with 60% labeled dataset by considering feature ranking approaches and all extracted features as an input.
- The selection of the 60% labeled data ratio was empirically determined through a series of controlled experiments. Specifically, the model performance is evaluated across multiple labeled-to-unlabeled data splits (e.g., 20%, 40%, 60%, 80% labeled data) while keeping other parameters constant. The results indicated that performance improved significantly up to 60% labeled data, after which the marginal gains became negligible or saturated. This suggests that 60% provides a suitable balance between leveraging labeled data and benefiting from semi-supervised learning on unlabeled samples. This threshold is based on experimental observation and performance trends on the evaluated datasets.

**Fig. 7:**
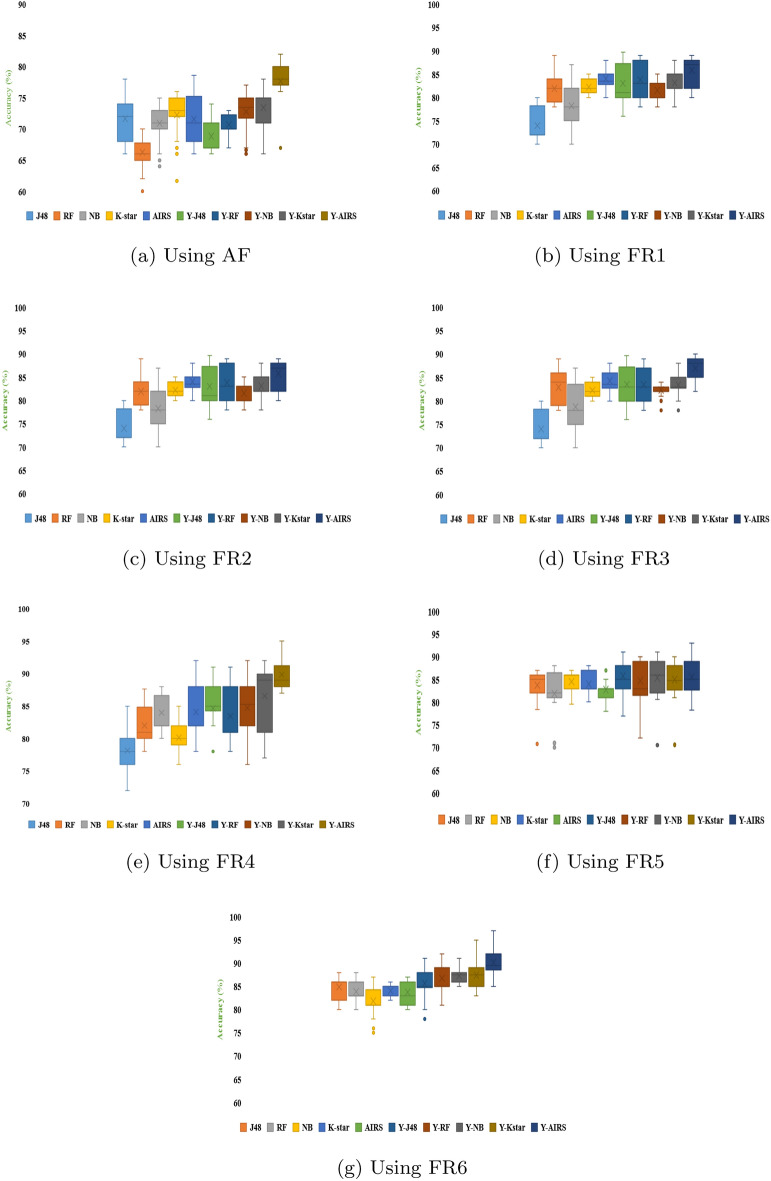
Box-plot diagram of Accuracy with optimal number of labeled dataset (i.e., 60%).

**Fig. 8:**
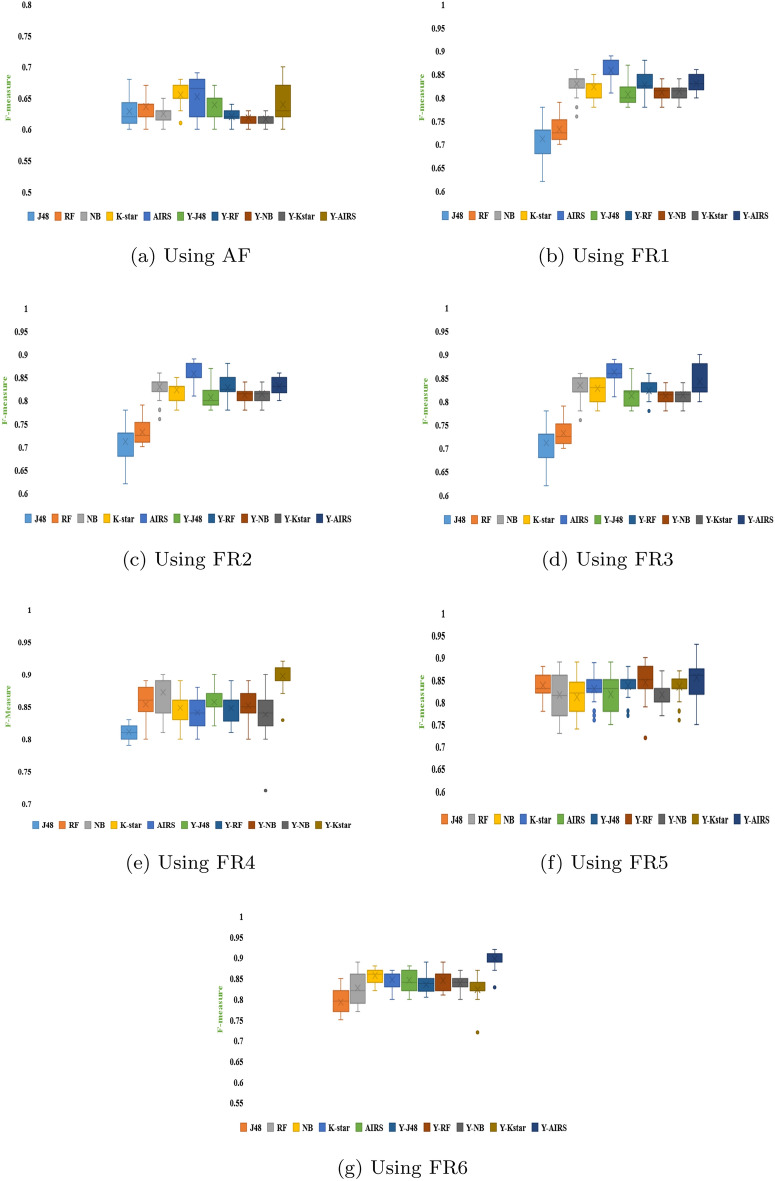
Box-plot diagram of F-measure with optimal number of labeled dataset (i.e., 60%).

In this study, ten distinct machine learning algorithms and six different feature selection approaches are utilized to identify features that enhance Android malware detection. To evaluate which of the developed malware detection models is most effective, box-plot diagrams are created for each individual model. Box-plot diagrams help identify the most suitable model for Android malware detection by analyzing the median value and the presence of fewer outliers. Box-plot diagrams for Accuracy and F-measure for developed models are shown in Figs. 7 and 8. Machine learning algorithms are presented in the *x*-axis of the diagrams. Based on the box-plot diagram, we are having the following observations:

- The model constructed using ten distinct machine learning algorithms and FR6 achieved a higher median value along with fewer outliers.
- Based on the box-plot diagrams in Figs. 7 and 8, the malware detection model developed using FR6 as the feature selection approach achieves higher accuracy as compared to other approaches.
- Based on the box-plot diagrams, it is observed that the model built using our proposed machine learning algorithm and FR6 has a higher median value and fewer outliers. This implies that the Android malware detection model utilizing PCA for detecting malware-infected apps achieved a higher detection rate compared to other approaches.

**Table 8:** Performance comparison of various feature selection approaches based on their computed mean difference.

Accuracy	AF	FR1	FR2	FR3	FR4	FR5	FR6
AF	0	-1.8	– 0.85	– 0.82	– 1.72	– 1.82	– 4.23
FR1	1.67	0	0.67	0.77	– 0.67	– 0.79	– 2.8
FR2	0.76	– 0.67	0	0.46	– 2.3	– 1.99	– 3.89
FR3	0.65	– 0.57	– 0.192	0	– 1.23	– 1.42	– 3.98
FR4	1.78	0.67	1.132	1.136	0	– 2.23	– 2.89
FR5	1.788	0.87	1.72	1.76	0.89	0	– 2.779
FR6	4.5	2.88	2.82	3.89	2.78	2.78	0

### Comparison of experimental results

To evaluate the effectiveness of the implemented machine learning algorithms and feature selection approaches, and to determine whether all techniques perform equally well, we employed a pairwise *t*-test in our study.

**Fig. 9:**
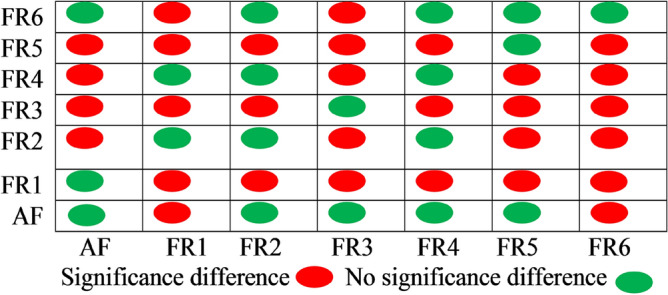
t-test analysis on feature selection techniques.

**Fig. 10:**
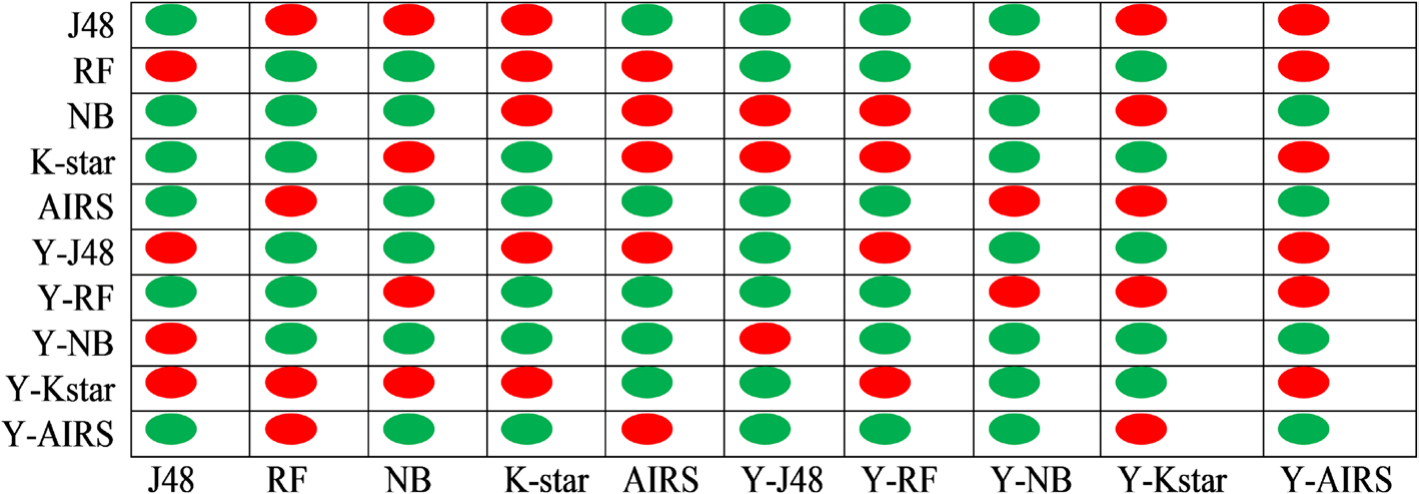
*t*-test analysis on Machine Learning Algorithms.

1. *Feature selection approaches:* In this study, for each implemented feature selection approach, two distinct sets were created, with each approach containing 300 unique data points (30 datasets *×* 10 machine learning techniques). A *t*-test was conducted on the different feature selection approaches, and the corresponding p-values were analyzed to assess their statistical significance. The results of the *t*-test are shown in Fig. 9. In the figure, two different symbols represent the p-value: a red-filled circle indicates a p-value *łe* 0.05 (relevant difference), while a green-filled circle indicates a p-value >0.05 (no relevant difference). From Fig. 9, it is evident that many cells contain green circles, indicating no significant difference between the implemented feature selection approaches. Additionally, by analyzing the mean difference values in Table 8, we observed that feature sets obtained using FR6 yielded the best results compared to other feature selection approaches.

2. *Machine Learning Techniques*: In our study, six different feature subsets are implemented (i.e., 6 feature selection approaches + 1 considering all features) on thirty different Android app datasets, evaluating two performance parameters: Accuracy and F-measure. This resulted in 210 data points (i.e., (1 considering all features + 6 feature ranking methods) *×* 30 datasets). Fig. 10 presents the results of the t-test analysis. Based on Fig. 10, it is evident that there is a significant difference among these techniques, as the p-value is smaller than 0.05. Additionally, by analyzing the mean value differences shown in Table 9, we found that Y-AIRS delivered the best performance when compared to the other machine learning techniques.

3. *Model-wise study*: The ablation study evaluates four configurations: (i) baseline model without AIRS, YATSI, and PCA, (ii) baseline + PCA, (iii) PCA + YATSI, and (iv) full model (PCA + YATSI + AIRS). The experiment results show that PCA improves the performance by 10% while reducing feature redundancy and noise, leading to better generalization. The inclusion of YATSI further enhances performance by 20% effectively leveraging unlabeled data through semi-supervised learning. Finally, integrating AIRS provides the most significant gain by introducing affinity-based selection and memory evolution, which improves the accuracy by 80% and also robustness against noisy pseudo-labels and evolving malware patterns.

###  Evaluation of proposed framework (i.e.,YATSIDroid)

####  Comparison of results on the basis of previously developed approaches or frameworks

In addition to developing a model using various machine learning algorithms, this study compares our approach with existing frameworks from the literature. Previous frameworks often relied on limited datasets, focusing on specific categories of Android apps and training models on a restricted number of malware families. In contrast, our proposed model was trained on the behaviors of 141 distinct malware families. Table 10 presents a comparison between previously developed frameworks and our approach. While earlier frameworks achieved moderate detection rates, slightly lower than our proposed model, they were typically trained on fully labeled datasets. Despite this, our model, “YATSIDroid”, achieved an impressive 98.5% detection rate when tested on real-world apps.

Moreover the approaches present in the literature unlike RNPDroid^19^ and PRAZDroid^21^, which primarily rely on static permission analysis combined with statistical or rule-based techniques, our approach adopts a semi-supervised AIRS-based learning framework that can leverage both labeled and unlabeled data. While prior methods depend on predefined thresholds and do not explicitly handle unlabeled data or noise, our model incorporates confidence-based pseudo-labeling, affinity-driven filtering, and iterative refinement, enabling adaptive learning and improved robustness against noisy samples and evolving malware patterns. Additionally, our framework supports feature ranking and multi-feature integration, providing enhanced generalization and detection capability beyond traditional permission-only analysis.

####  Comparison of results on the basis of different AntiVirus scanners

To validate the proposed framework, ten different freely available antivirus scanners are considered for the experiment. In our study, the comparison with commercial antivirus engines was conducted using VirusTotal, rather than executing individual antivirus products locally. Specifically, each apps in the dataset was submitted to VirusTotal, which aggregates results from multiple antivirus engines. The detection outcomes (e.g., number of engines flagging a sample as malicious) were then used as a reference for comparison.

There are main three reasons to select VirusTotal i.e., (1) Running multiple commercial antivirus engines locally with consistent configurations is challenging and resource-intensive. (2) VirusTotal provides a widely accepted platform for comparative analysis across numerous engines under uniform conditions. and (3) Using VirusTotal ensures that other researchers can replicate the evaluation using the same interface and methodology.

The experiment was performed by using well-established Drebin dataset. Our proposed framework demonstrated superior performance compared to several antivirus scanners evaluated in this study. Table 11 demonstrates the results of the experiment conducted with antivirus scanners. The detection rates of the antivirus scanners varied significantly. Among the scanners tested, the best one could only detect 96.2% of malware-infected apps, while some scanners identified as few as 82% of the malicious samples, likely due to their lack of specialization in detecting Android malware. In contrast, our proposed framework, YATSIDROID, detected 98.5% of the malicious samples, outperforming the top commercial antivirus scanners tested. Based on these empirical results, it can be concluded that our proposed framework is more effective in detecting malware-infected apps rather than traditional signature-based antivirus scanners.

####  Detection based on known and unknown malware families

To evaluate that our proposed framework has the ability to detect unknown malware or not. We implied a family-wise holdout (leave-one-family-out) strategy. Specifically, we partition the dataset such that a subset of malware families is completely excluded from the training phase and used only during testing. This ensures that the model has no prior exposure to these families, thereby simulating a realistic zero-day detection scenario. The model is trained on the remaining known families along with benign samples, and its performance is then evaluated on both seen (known) and unseen (unknown) malware families. Moreover, there is no overlap of samples or family-specific patterns between the training and testing sets to prevent data leakage.

**Fig. 11:**
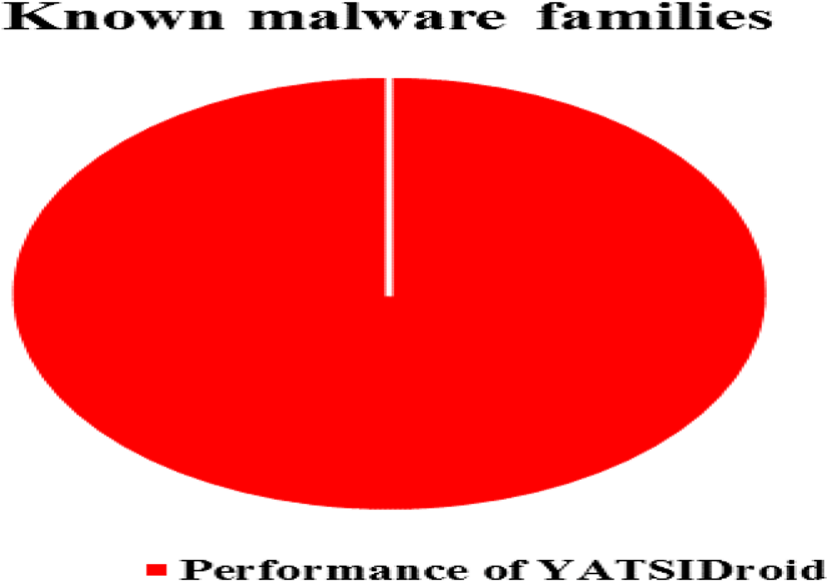
Outcome for known malware families.

**Fig. 12:**
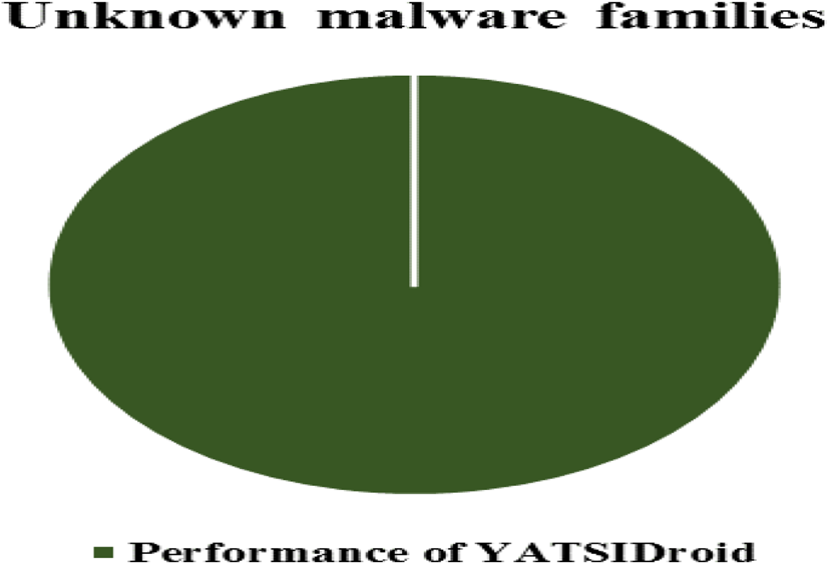
Outcome for unknown malware families.

YATSIDroid, developed using 141 malware families, achieves a detection rate of 100% for known malware families and 98.8% for unknown malware families. Figures 11 and 12 represent its performance in detecting these malware families.

**Table 9:** Average performance difference across various machine learning algorithms.

Accuracy	J48	RF	NB	K-star	AIRS	Y-J48	Y-RF	Y-NB	Y-K-star	Y-AIRS
J48	0	– 2.2	– 2.08	– 2.88	– 2.01	– 2.88	– 2.99	– 2.87	– 2.99	– 3.99
RF	2.89	0	– 2.07	– 1.88	– 1.99	– 2.78	– 2.49	– 2.67	– 2.91	– 3.79
NB	1.88	2.21	0	– 2.18	– 2.31	– 2.99	– 2.67	– 2.57	– 2.49	– 3.09
K-star	2.08	2.89	2.11	0	– 1.88	– 2.79	– 2.97	– 2.88	– 2.89	– 3.89
AIRS	1.88	2.21	2.11	– 2.08	0	– 2.99	– 2.67	– 2.57	– 2.49	– 3.09
Y-J48	2.87	2.99	2.98	2.68	2.88	0	– 2.87	– 2.97	– 2.29	– 2.99
Y-RF	2.07	2.29	2.28	2.38	2.48	1.88	0	– 2.37	– 2.10	– 2.79
Y-NB	2.87	2.89	2.68	2.12	2.23	1.78	1.07	0	– 2.18	– 2.59
Y-Kstar	2.37	2.19	2.48	2.62	2.83	2.08	2.37	2.18	0	– 1.79
Y-AIRS	3.37	3.19	2.89	2.92	2.33	1.89	1.97	1.88	1.898	0

**Table 10:** Comparison of the proposed framework, YATSIDroid, with previously developed frameworks and approaches.

Framework/ approach	Methodology	Goal	dataset	Detection rate	Labeled dataset
Andromaly^9^	Dynamic and Profile-based	Detection	Very-Limited	High	100%
AndroSimilar^74^	Static	Detection	Restricted	Moderate	100%
Andrubis^75^	Static, Dynamic, Profile-based and Behavioural	Analysis and Detection	Higher	Moderate	100%
Aurasium^76^	Dynamic and Behavioural	Detection	Restricted	High	100%
CopperDroid^77^	Dynamic, System/API	Analysis and Detection	Restricted and VMI	Moderate	100%
Crowdroid^8^	Dynamic, API/System call and Behavioural	Detection	Very-Limited	High	100%
Paranoid Android^78^	Behavioural and Dynamic	Detection	Restricted	—	100%
TaintDroid^79^	Dynamic System call/API and Behavioural	Detection	Very-Limited	Moderate	100%
HinDroid^25^	Dynamic and API	Detection	Restricted	Moderate	100%
MalDozer^80^	Dynamic	Detection	Restricted	Moderate	100%
HEMD^81^	Dynamic and Permissions	Detection	Restricted	Moderate	100%
DroidDet^29^	Static	Detection	Restricted	Moderate	100%
Wei Wang^31^	Dynamic	Detection	Restricted	Moderate	100%
MalInsight^82^	Dynamic	Detection	Restricted	High	100%
DroidRL^37^	Used dynamic features	Detection	Restricted	High	100%
PermDroid^2^	Used dynamic features	Detection	Restricted	High	100%
YATSIDroid (Proposed framework)	Dynamic, API calls, Permissions, user-rating and Number of user download app	Detection	Unlimited	Higher (with limited labeled dataset)	60%

YATSIDroid used a limited labeled dataset as input for developing the malware detection model, in contrast to previously developed frameworks that relied on a fully labeled dataset.

**Table 11:** The detection rate of the proposed framework, YATSIDroid, in conjunction with different antivirus scanners.

Name of the Anti-virus	Detection rate ( in %)	Speed to detect malware in sec
Cyren	80.6	60.9
Ikarus	80.68	61.9
VIPRE	88.7	48.7
McAfee	87.6	39.8
AVG	92.7	31.2
AVware	90.8	32.0
ESET NOD32	91.9	22.0
CAT QuickHeal	93.9	31.2
AegisLab	96.1	32.4
NANO Antivirus	95.2	21.0
YATSIDroid (our proposed approach)	98.5	12

Drebin dataset^83^ is used to test the framework and different anti-virus scanners.

####  Experimental findings

This subsection presents a detailed conclusion of our experimental work. The empirical study was conducted on thirty different categories of Android apps, employing ten distinct machine learning algorithms. Based on the experimental outcomes, this study effectively addresses the questions presented in subsection 2.1.

**RQ1:** In this study, ten different machine learning algorithms were implemented to develop a model for classifying apps as either benign or malware. Based on Figs. 5 and 6, it can be inferred that the model developed using YATSI with AIRS, along with features selected by FR6, outperforms the other models.

**RQ2:** To address RQ2, Figs. 7, 8, and Table 11 were analyzed. These analyses indicate that our proposed framework effectively identifies malware-infected apps in real-world scenarios.

**RQ3:** In this research paper, six distinct feature ranking methods are applied to identify a smaller subset of relevant features. These methods are used to select relevant feature subsets that contribute to building a model for classifying an app as benign or malicious. As shown in Figs. 5 and 6, in several cases, the reduced feature sets outperform the complete set of extracted features in developing an effective malware detection model.

**RQ4:** This research paper uses six different feature ranking approaches to identify a reduced subset of features. The *t*-test analysis results show that the PCA-based feature selection approach achieves a higher detection rate compared to the other methods.

**RQ5:** An analysis of Figs. 5 and 6, along with Tables 12,13, 14, 15, 16, 17, 18, 19, 20, 21, 22, 23, 24, 25, 26, 27, 28, 29, 30, 31, 32, 33, 34, 35, 36, 37, 38 and 39, indicates that models built using features selected through feature ranking approaches achieve a higher detection rate than those utilizing all extracted features.

**Fig. 13:**
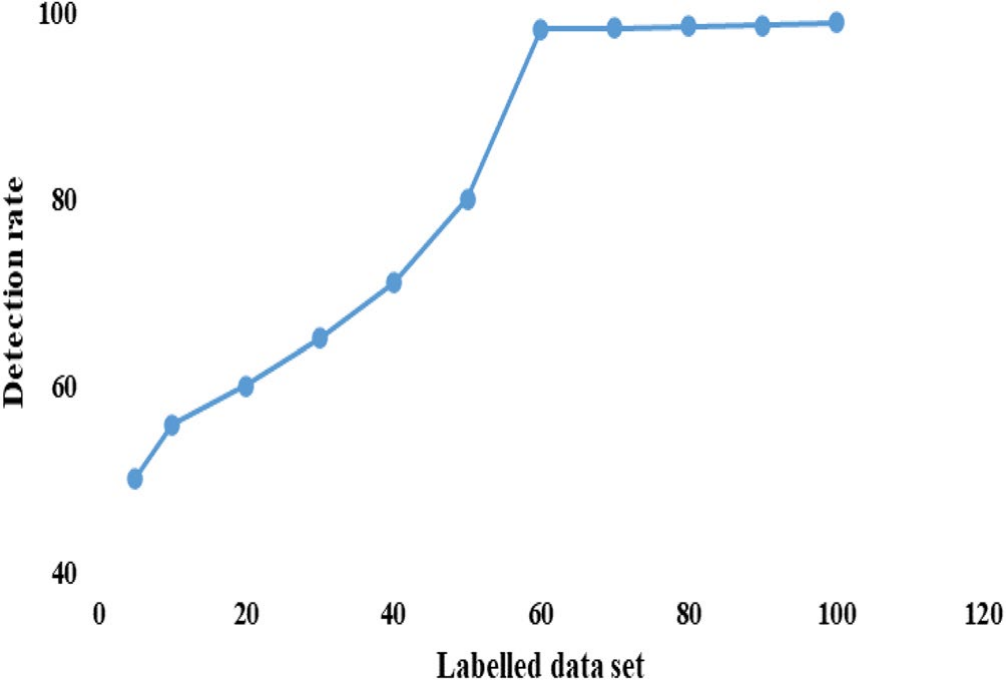
Detection rate with different percentage of labeled dataset and with vary value of *k*-nearest neighbours(KNN).

**Fig. 14:**
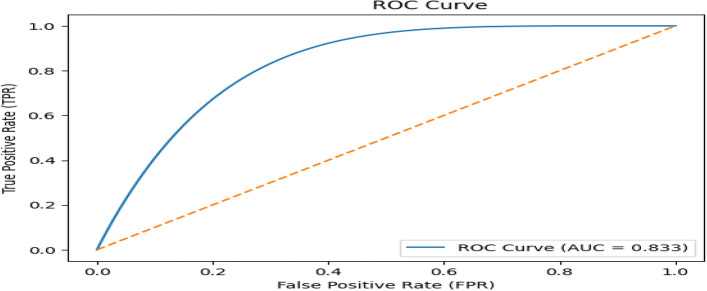
AUC curve for optimal number of labeled dataset.

**RQ6:** To answer this question, we performed an experiment by varying the value of labeled dataset (5%,10%,....100%). Fig. 13 shows the detection rate (i.e., accuracy) with the different labeled dataset. An optimal number of labeled dataset proposed by our machine learning algorithm is 60%. When we compared the detection rate of 60% labeled dataset with 100% labeled dataset the difference is negligible. To make our evaluation more stronger we also include, ROC-AUC metric (In Appendix B (Table 41 and Figure 14)) is used to assess the model’s discriminative ability across varying classification thresholds, while MCC provides a balanced measure by considering all elements of the confusion matrix, making it especially suitable for imbalanced datasets.

## Conclusion

This study help in identifying malware-infected apps by implementing relevant features identified by feature selection techniques. Empirical studies were conducted using various categories of Android applications.

The following are the submissions based on the experiments conducted:

- Empirical results reveal that identifying a small subset of features is feasible. The model built using significant features can identify malware-infected apps with a higher detection rate and a lower rate of misclassification errors.
- Based on empirical studies, it is conclude that feature reduction is achievable through feature selection approaches. Experimental results further demonstrate that malware detection models developed using feature selection approaches achieve a higher detection rate compared to models utilizing all extracted features.
- This study also highlights that, based on the *t*-test, models developed using different machine learning approaches exhibit significant differences.
- This research paper also highlights that models built using significant features are able to detect more malware-infected apps than those that utilize all extracted features.
- The efficiency of the malware detection framework is dependent on the feature selection approaches selected for the study.
- Based on the mean difference, it is observed that models developed using significant features yield a higher detection rate as compared to those using all features.
- In terms of computational complexity, the proposed method is primarily based on lightweight static feature extraction and efficient feature ranking, which ensures low preprocessing overhead. The integration of PCA reduces dimensionality, thereby lowering training time and memory requirements. Additionally, the semi-supervised component (YATSI) leverages unlabeled data without significantly increasing computational cost, while the AIRS-based module operates with controlled memory cell populations, ensuring scalability. From a practical perspective, the framework can be efficiently applied to large-scale datasets, as training can be performed offline and updated incrementally with new data. The modular design also allows parallelization of feature extraction and model training. Overall, the method demonstrates good scalability with respect to dataset size and feature dimensionality, making it suitable for real-world Android malware detection scenarios. This study emphasizes the detection of malware-infected apps. Additionally, it can be extended to examine the behavior of individual features to determine whether a particular feature is effective in detecting malware-infected apps. Furthermore, this work can be expanded by implementing additional soft computing techniques to achieve a better detection rate.

## Data Availability

The datasets used in this study are publicly available in the Mendeley Data repository with the following DOIs:10.17632/mg5c8jxbhm.2 and 10.17632/rvjptkrc34.2.

## Appendices

### Appendix A

F-Measure and Accuracy using 5%, 10%, ... 100% are presented in the appendix from Tables 12, 13, 14, 15, 16, 17, 18, 19, 20, 21, 22, 23, 24, 25, 26, 27, 28, 29, 30, 31, 32, 33, 34, 35, 36, 37 and 38. Additionally, we calculate F-measure and accuracy with an optimal number of labeled data points.

**Table 12:** Accuracy and F-measure using AF with 5% labeled dataset.

Accuracy												F-measure								
ID	J48	RF	NB	K-star	AIRS	Y-J48	Y-RF	Y-NB	Y-Kstar	Y-AIRS	J48	RF	NB	K-star	AIRS	Y-J48	Y-RF	Y-NB	Y-Kstar	Y-AIRS
DS1	55.3	54.4	55.5	55.5	51.5	**57.4**	55	**57.4**	55.5	**57.4**	0.51	0.50	0.51	0.51	0.52	**0.58**	0.55	**0.58**	0.52	**0.58**
DS2	50.3	52.5	58.5	55.9	50.5	57.4	58	59.4	55	**59.9**	0.50	0.51	0.52	0.55	0.50	0.56	0.56	0.53	0.55	**0.57**
DS3	51.0	50.8	52.5	55.0	53.5	57.4	58	57.4	56	**57.9**	0.51	0.50	0.51	0.50	0.51	0.55	0.57	0.56	0.55	**0.58**
DS4	51.3	52.3	58.5	56.5	56.5	59.4	58	58.4	58.8	**59.8**	0.50	0.51	0.50	0.52	0.50	0.58	0.58	0.57	0.57	**0.58**
DS5	50.0	51.8	50.5	52.5	53.5	59.4	58	58.4	58.8	**59.9**	0.53	0.52	0.51	0.50	0.52	0.53	0.56	0.56	0.56	**0.57**
DS6	50.0	52.3	50.5	52.5	52.8	56.4	57	58.4	57.8	**59.2**	0.50	0.52	0.53	0.52	0.53	0.57	0.58	0.58	0.57	**0.59**
DS7	51.0	52.3	56.5	55.5	55.5	59.4	59	59.4	59.8	**60.0**	0.50	0.51	0.50	0.52	0.50	0.57	0.56	0.58	0.58	**0.59**
DS8	52.3	51.8	58.5	55.5	50.55	58.4	58	58.4	58	**59.8**	0.50	0.51	0.56	0.53	0.53	0.56	0.56	0.57	0.57	**0.58**
DS9	50.3	52.3	56.5	55.5	50.55	55.4	56	57.4	57	**58**	0.50	0.51	0.54	0.55	0.50	0.57	0.57	0.58	0.57	**0.59**
DS10	50.0	51.8	50.5	52.5	53.5	59.4	58	58.4	58.8	**59.9**	0.51	0.53	0.54	0.50	0.52	0.57	0.57	0.56	0.57	**0.58**
DS11	51.0	54.3	58.5	55.5	50.5	58.4	52.8	57.4	57.8	**58.9**	0.50	0.51	0.50	0.52	0.50	0.51	0.57	0.56	0.56	**0.58**
DS12	53.0	54.3	57.5	57.5	57.8	58.4	58	57.4	59	**59.9**	0.51	0.50	0.51	0.50	0.51	0.57	0.59	0.58	0.58	**0.60**
DS13	50.3	55.3	58.5	55.5	57.5	59.4	59.8	58.7	58	**60.5**	0.50	0.51	0.59	0.58	0.50	0.58	0.57	0.57	0.57	**0.6**
DS14	51.0	50.3	53.5	57.5	52.5	57.4	58	58.4	58	**59.9**	0.51	0.50	0.51	0.50	0.51	0.58	0.59	0.58	0.57	**0.6**
DS15	50.0	51.8	49.8	50.5	51.5	57.4	58	58.4	58.8	**59.9**	0.51	0.52	0.51	0.50	0.52	0.57	0.58	0.56	0.57	**0.58**
DS16	51.0	52.3	52.5	53.5	52.5	54.4	57	56.4	56.8	**58.2**	0.50	0.51	0.50	0.52	0.50	0.55	0.58	0.57	0.55	**0.57**
DS17	50.0	52.4	53.5	52.8	53.5	57.4	57.8	54.4	55.8	**58.9**	0.49	0.51	0.50	0.52	0.51	0.55	0.53	0.53	0.52	**0.57**
DS18	51.3	52.7	53.5	55.5	54.5	56.4	57	57.4	57	**58**	0.50	0.51	0.52	0.52	0.53	0.55	0.56	0.55	0.54	**0.56**
DS19	50.3	52.3	53.5	53.5	52.5	57.4	57	57.4	57	**58**	0.50	0.51	0.52	0.53	0.50	0.55	0.56	0.57	0.55	**0.59**
DS20	51.0	52.3	53.5	54.5	55.5	56.4	58.8	58.4	58.8	**59.6**	0.50	0.51	0.50	0.52	0.50	0.55	0.58	0.58	0.58	**0.59**
DS21	50.3	55.3	54.5	51.5	53.5	57.4	58	59.4	58	**59.8**	0.50	0.51	0.52	0.52	0.50	0.58	0.58	0.57	0.58	**0.59**
DS22	51.0	53.7	54.5	54.3	55.5	58.4	58.9	58.4	59.8	**60.4**	0.50	0.51	0.50	0.52	0.50	0.57	0.59	0.54	0.55	**0.59**
DS23	54.0	55.3	53.5	54.5	53.5	59.4	58.9	58.4	58.8	**61.9**	0.50	0.52	0.51	0.50	0.52	0.57	0.56	0.53	0.55	**0.58**
DS24	51.0	52.3	53.5	55.5	50.5	56.1	57	57.4	58.8	**59.0**	0.50	0.51	0.50	0.52	0.50	0.57	0.58	0.56	0.56	**0.59**
DS25	53.3	52.8	51.5	50.5	51.5	53.4	56	55.4	55	**57.1**	0.50	0.51	0.52	0.53	0.50	0.54	0.55	0.55	0.54	**0.58**
DS26	51.0	53.3	50.7	53.5	54.5	58.4	57.8	57.4	56.8	**59.0**	0.50	0.51	0.50	0.52	0.50	0.56	0.55	0.57	0.56	**0.56**
DS27	50.3	52.3	54.5	55.5	56.5	56.4	57.8	56.4	58	**58.9**	0.50	0.51	0.53	0.52	0.50	0.54	0.56	0.57	0.56	**0.58**
DS28	51.0	52.3	57.5	56.5	55.5	56.4	56.1	56.4	56	**58.9**	0.51	0.50	0.51	0.50	0.51	0.54	0.55	0.55	0.53	**0.58**
DS29	50.3	52.8	54.5	55.5	55.5	57.4	55	55.4	56	**57.8**	0.50	0.51	0.54	0.53	0.50	0.52	0.52	0.56	0.56	**0.57**
DS30	51.0	52.8	52.9	53.0	53.8	56.4	56.7	57.4	57.8	**58.2**	0.52	0.52	0.53	0.53	0.55	0.56	0.56	0.57	0.58	**0.59**

Significant values are in [bold].

**Table 13:** Accuracy and F-measure using AF with 10% labeled dataset.

Accuracy											F-measure									
ID	J48	RF	NB	K-star	AIRS	Y-J48	Y-RF	Y-NB	Y-Kstar	Y-AIRS	J48	RF	NB	K-star	AIRS	Y-J48	Y-RF	Y-NB	Y-Kstar	Y-AIRS
DS1	52.3	54.8	54.6	58.5	57.5	**60.4**	59.8	**60.4**	59.5	**60.4**	0.53	0.52	0.53	0.53	0.54	**0.63**	0.59	**0.63**	0.59	**0.63**
DS2	51.3	53.3	51.5	52.5	52.7	57.4	58	58.4	58.9	**60.8**	0.52	0.53	0.50	0.51	0.52	0.58	0.59	0.59	0.6	**0.61**
DS3	52.0	51.7	53.5	52.5	52.5	58.4	58.9	59.4	59	**59.9**	0.52	0.51	0.53	0.52	0.53	0.55	0.56	0.57	0.57	**0.59**
DS4	52.0	53.3	54.5	56.5	55.5	57.4	57.8	57.4	58.8	**59.8**	0.52	0.53	0.54	0.54	0.55	0.57	0.56	0.54	0.58	**0.59**
DS5	53.0	54.3	54.5	54.5	57.5	58.4	57	58.4	58.8	**60.9**	0.50	0.53	0.54	0.53	0.53	0.56	0.55	0.57	0.58	**0.60**
DS6	52.0	54.3	51.5	53.5	52.8	58.4	59	59.4	59.8	**60.2**	0.50	0.53	0.51	0.52	0.51	0.58	0.58	0.57	0.58	**0.61**
DS7	53.1	53.3	54.5	56.5	57.5	59.4	59	58.4	58.8	**59.8**	0.52	0.53	0.52	0.51	0.55	0.58	0.57	0.57	0.58	**0.59**
DS8	51.3	52.3	53.5	52.5	54.5	59.4	58.7	59.4	59.8	**61**	0.50	0.52	0.52	0.51	0.52	0.54	0.57	0.57	0.58	**0.59**
DS9	52.0	53.3	51.5	53.5	54.5	57.4	58	58.4	57.8	**59.8**	0.52	0.53	0.52	0.52	0.54	0.56	0.57	0.58	0.57	**0.60**
DS10	52.0	53.3	51.5	52.5	51.5	54.4	57.8	57.9	58.8	**60.8**	0.52	0.53	0.54	0.53	0.55	0.57	0.59	0.57	0.58	**0.61**
DS11	52.0	56.3	54.5	52.5	57.5	58.4	58.3	57.4	58.8	**59.9**	0.52	0.53	0.53	0.53	0.55	0.57	0.56	0.57	0.58	**0.59**
DS12	51.0	50.3	51.5	50.5	51.5	57.4	56.8	56.4	58	**59.9**	0.51	0.50	0.51	0.50	0.51	0.56	0.59	0.57	0.58	**0.60**
DS13	51.3	53.3	56.5	55.5	54.5	58.4	58.7	59.4	59.9	**61.5**	0.50	0.51	0.55	0.57	0.56	0.58	0.58	0.59	0.59	**0.61**
DS14	53.8	55.3	56.5	56.5	57.5	59.4	59	59.4	58.8	**60.8**	0.52	0.53	0.54	0.54	0.55	0.57	0.56	0.56	0.55	**0.59**
DS15	51.0	51.3	54.5	55.5	57.5	59.4	58.8	58.4	59.8	**59.9**	0.50	0.52	0.51	0.50	0.52	0.54	0.56	0.57	0.58	**0.59**
DS16	53.0	55.3	53.5	54.7	55.5	59.4	58.9	57.4	56.8	**59.9**	0.50	0.51	0.50	0.52	0.50	0.56	0.58	0.57	0.57	**0.59**
DS17	50.0	51.3	51.5	52.5	53.5	58.4	59.0	59.4	59.8	**60.9**	0.53	0.52	0.51	0.50	0.52	0.59	0.58	0.56	0.57	**0.60**
DS18	52.5	52.7	53.7	52.5	52.5	57.4	58	59.4	59.3	**61.8**	0.50	0.51	0.54	0.55	0.50	0.57	0.58	0.59	0.54	**0.59**
DS19	51.9	53.8	55.7	52.5	54.7	57.4	58	59.4	59.8	**60.8**	0.50	0.51	0.53	0.54	0.50	0.57	0.58	0.58	0.58	**0.59**
DS20	51.0	55.3	54.5	56.1	50.5	57.4	58.8	58.4	59.8	**60.9**	0.50	0.51	0.50	0.52	0.50	0.57	0.58	0.56	0.57	**0.59**
DS21	52.0	53.3	53.8	55.5	55.8	59.4	59.8	59.4	60.8	**61.2**	0.50	0.53	0.53	0.53	0.53	0.59	0.58	0.58	0.57	**0.60**
DS22	51.0	53.3	53.5	55.5	50.5	58.4	58.9	58.4	59.8	**60.8**	0.52	0.53	0.53	0.54	0.52	0.59	0.57	0.58	0.58	**0.59**
DS23	55.0	57.9	58.5	54.5	56.8	59.4	60	60.4	60.8	**61.2**	0.50	0.53	0.53	0.53	0.53	0.58	0.58	0.57	0.59	**0.60**
DS24	53.0	55.1	53.8	55.5	57.5	59.4	59.8	59.4	59.8	**60.5**	0.53	0.54	0.55	0.55	0.54	0.57	0.54	0.58	0.57	**0.59**
DS25	50.8	52.9	53.8	56.5	57.5	59.4	59.8	58.4	58.8	**60.9**	0.50	0.51	0.59	0.58	0.50	0.57	0.56	0.58	0.59	**0.60**
DS26	52.8	54.3	53.5	55.5	54.8	59.4	58.9	59.4	58.8	**59.8**	0.50	0.53	0.53	0.51	0.51	0.57	0.58	0.57	0.56	**0.59**
DS27	53.0	54.3	53.5	55.5	54.8	58.4	57	58.4	58.8	**59.2**	0.50	0.53	0.53	0.53	0.53	0.57	0.57	0.56	0.58	**0.59**
DS28	53.4	51.3	54.5	53.5	52.5	57.4	58	57.4	58	**59.9**	0.51	0.50	0.51	0.50	0.51	0.53	0.59	0.55	0.56	**0.59**
DS29	54.3	55.3	56.5	53.5	55.5	57.4	57	58.4	54	**58.9**	0.50	0.51	0.59	0.58	0.50	0.54	0.56	0.54	0.53	**0.58**
DS30	53.0	54.3	53.5	55.5	54.8	58.4	58	58.4	58.8	**59.2**	0.50	0.53	0.53	0.53	0.53	0.57	0.57	0.57	0.58	**0.59**

Significant values are in [bold].

**Table 14:** Accuracy and F-measure using AF with optimal number of labeled dataset (i.e., 60%).

Accuracy											F-measure									
ID	J48	RF	NB	K-star	AIRS	Y-J48	Y-RF	Y-NB	Y-Kstar	Y-AIRS	J48	RF	NB	K-star	AIRS	Y-J48	Y-RF	Y-NB	Y-Kstar	Y-AIRS
DS1	72.33	78.33	76.66	78.57	77.66	**78.4**	78.0	**78.4**	73.6	**78.4**	0.7	0.72	0.75	0.71	0.74	**0.78**	0.73	**0.78**	0.72	**0.78**
DS2	72.33	73.33	70.77	70.8	72.77	82.4	83	83.4	81.9	**83.8**	0.72	0.73	0.70	0.71	0.72	0.77	0.79	0.82	0.83	**0.84**
DS3	72.0	72.33	70.77	70.8	72.77	82.4	83	83.4	83	**84.9**	0.72	0.72	0.73	0.72	0.73	0.82	0.81	0.82	0.83	**0.84**
DS4	72.0	73.33	71.77	72.8	72.7	80.4	83	83.4	83.8	**83.8**	0.72	0.73	0.74	0.74	0.75	0.83	0.80	0.81	0.82	**0.83**
DS5	73.0	74.33	74.7	74.8	75.7	80.4	82	83.4	84.8	**84.9**	0.70	0.73	0.74	0.73	0.73	0.82	0.83	0.83	0.82	**0.84**
DS7	72.0	74.33	73.7	75.8	74.8	83.4	84	83.4	84.8	**85.2**	0.70	0.73	0.73	0.73	0.73	0.83	0.84	0.82	0.84	**0.85**
DS8	72.0	73.33	71.77	72.8	72.7	80.4	83	83.4	83.8	**83.8**	0.72	0.73	0.74	0.74	0.75	0.83	0.80	0.81	0.82	**0.83**
DS8	70.33	72.33	71.77	72.8	74.77	83.4	82	84.4	83	**84**	0.70	0.72	0.72	0.71	0.72	0.73	0.75	0.80	0.82	**0.84**
DS9	72.0	73.33	71.77	72.8	72.7	80.4	83	83.4	83.8	**83.8**	0.72	0.73	0.74	0.74	0.75	0.83	0.80	0.81	0.82	**0.83**
DS10	72.0	73.33	71.77	72.8	72.7	80.4	83	83.4	83.8	**83.8**	0.72	0.73	0.74	0.74	0.75	0.83	0.80	0.81	0.82	**0.83**
DS11	72.0	73.33	71.77	72.8	72.7	80.4	83	83.4	83.8	**83.8**	0.72	0.73	0.74	0.74	0.75	0.83	0.80	0.81	0.82	**0.83**
DS12	71.0	70.33	71.77	70.8	71.77	80.4	81	81.4	82	**82.9**	0.71	0.70	0.71	0.70	0.71	0.80	0.79	0.81	0.82	**0.83**
DS13	72.33	74.33	71.77	72.8	73.77	83.4	84	83.4	83	**84.8**	0.70	0.71	0.70	0.78	0.70	0.72	0.78	0.80	0.81	**0.82**
DS14	72.0	73.33	71.77	72.8	72.7	80.4	83	83.4	83.8	**83.8**	0.72	0.73	0.74	0.74	0.75	0.83	0.80	0.81	0.82	**0.83**
DS15	70.0	71.33	70.7	72.8	73.7	79.4	80	82.4	83.8	**83.9**	0.59	0.72	0.71	0.70	0.72	0.80	0.81	0.81	0.82	**0.83**
DS17	71.0	72.33	72.77	71.8	70.7	79.4	82	80.4	81.8	**83.9**	0.70	0.71	0.70	0.72	0.70	0.81	0.78	0.80	0.81	**0.84**
DS18	70.0	71.33	70.7	72.8	73.7	79.4	80	82.4	83.8	**83.9**	0.59	0.72	0.71	0.70	0.72	0.80	0.81	0.81	0.82	**0.83**
DS18	70.33	72.33	73.77	72.8	70.77	81.4	83	83.4	83	**83.8**	0.70	0.71	0.79	0.78	0.70	0.72	0.78	0.80	0.81	**0.82**
DS19	70.33	73.33	71.77	72.8	74.77	83.4	83	83.4	84	**85**	0.70	0.71	0.79	0.78	0.70	0.72	0.78	0.80	0.81	**0.82**
DS20	71.0	72.33	71.77	78.8	73.7	82.4	82.8	83.4	83.8	**83.9**	0.70	0.71	0.70	0.72	0.70	0.81	0.78	0.80	0.81	**0.84**
DS21	72.0	74.33	73.7	75.8	74.8	83.4	84	83.4	84.8	**85.2**	0.70	0.73	0.73	0.73	0.73	0.83	0.84	0.82	0.84	**0.85**
DS22	71.0	74.33	73.77	78.8	70.7	80.4	83	82.4	83.8	**84.0**	0.72	0.73	0.73	0.74	0.72	0.82	0.83	0.82	0.82	**0.85**
DS23	72.0	74.33	73.7	75.8	74.8	83.4	84	83.4	84.8	**85.2**	0.70	0.73	0.73	0.73	0.73	0.83	0.84	0.82	0.84	**0.85**
DS24	75.0	76.0	78.7	78.8	70.7	81.4	83	82.4	84.0	**87.8**	0.70	0.77	0.76	0.78	0.74	0.83	0.83	0.85	0.84	**0.86**
DS25	72.3	75.3	75.7	75.9	74.8	84.4	83	85.4	84	**85.9**	0.72	0.76	0.79	0.78	0.79	0.76	0.80	0.82	0.82	**0.84**
DS26	73.0	75.3	77.07	75.8	74.8	83.4	84	83.4	84.8	**85.2**	0.70	0.73	0.73	0.73	0.73	0.83	0.84	0.82	0.84	**0.85**
DS27	72.0	74.33	73.7	75.8	74.8	83.4	84	83.4	84.8	**85.2**	0.70	0.73	0.73	0.76	0.73	0.83	0.84	0.82	0.84	**0.88**
DS28	71.0	70.33	79.77	79.8	71.77	80.4	81	81.4	82	**82.9**	0.71	0.70	0.71	0.70	0.71	0.80	0.79	0.81	0.82	**0.85**
DS29	76.3	77.33	78.77	78.8	70.77	81.4	82	82.4	81	**83**	0.70	0.71	0.79	0.78	0.70	0.72	0.78	0.80	0.81	**0.83**
DS30	77.0	74.33	73.7	75.8	74.8	83.4	84	83.4	84.8	**85.2**	0.70	0.73	0.73	0.73	0.73	0.83	0.84	0.82	0.84	**0.86**

Significant values are in [bold].

**Table 15:** Accuracy and F-measure using AF with 100% labeled dataset.

Accuracy												F-measure								
ID	J48	RF	NB	K-star	AIRS	Y-J48	Y-RF	Y-NB	Y-Kstar	Y-AIRS	J48	RF	NB	K-star	AIRS	Y-J48	Y-RF	Y-NB	Y-Kstar	Y-AIRS
DS1	72.33	78.33	76.66	78.57	77.66	**78.4**	78.0	**78.4**	73.6	**78.4**	0.7	0.72	0.75	0.71	0.74	**0.78**	0.73	**0.78**	0.72	**0.78**
DS2	72.33	73.33	70.77	70.8	72.77	82.4	83	83.4	81.9	**83.8**	0.72	0.73	0.70	0.71	0.72	0.77	0.79	0.82	0.83	**0.84**
DS3	72.0	72.33	70.77	70.8	72.77	82.4	83	83.4	83	**84.9**	0.72	0.72	0.73	0.72	0.73	0.82	0.81	0.82	0.83	**0.84**
DS4	72.0	73.33	71.77	72.8	72.7	80.4	83	83.4	83.8	**83.8**	0.72	0.73	0.74	0.74	0.75	0.83	0.80	0.81	0.82	**0.83**
DS5	73.0	74.33	74.7	74.8	75.7	80.4	82	83.4	84.8	**84.9**	0.70	0.73	0.74	0.73	0.73	0.82	0.83	0.83	0.82	**0.84**
DS7	72.0	74.33	73.7	75.8	74.8	83.4	84	83.4	84.8	**85.2**	0.70	0.73	0.73	0.73	0.73	0.83	0.84	0.82	0.84	**0.85**
DS8	72.0	73.33	71.77	72.8	72.7	80.4	83	83.4	83.8	**83.8**	0.72	0.73	0.74	0.74	0.75	0.83	0.80	0.81	0.82	**0.83**
DS8	70.33	72.33	71.77	72.8	74.77	83.4	82	84.4	83	**84**	0.70	0.72	0.72	0.71	0.72	0.73	0.75	0.80	0.82	**0.84**
DS9	72.0	73.33	71.77	72.8	72.7	80.4	83	83.4	83.8	**83.8**	0.72	0.73	0.74	0.74	0.75	0.83	0.80	0.81	0.82	**0.83**
DS10	72.0	73.33	71.77	72.8	72.7	80.4	83	83.4	83.8	**83.8**	0.72	0.73	0.74	0.74	0.75	0.83	0.80	0.81	0.82	**0.83**
DS11	72.0	73.33	71.77	72.8	72.7	80.4	83	83.4	83.8	**83.8**	0.72	0.73	0.74	0.74	0.75	0.83	0.80	0.81	0.82	**0.83**
DS12	71.0	70.33	71.77	70.8	71.77	80.4	81	81.4	82	**82.9**	0.71	0.70	0.71	0.70	0.71	0.80	0.79	0.81	0.82	**0.83**
DS13	72.33	74.33	71.77	72.8	73.77	83.4	84	83.4	83	**84.8**	0.70	0.71	0.70	0.78	0.70	0.72	0.78	0.80	0.81	**0.82**
DS14	72.0	73.33	71.77	72.8	72.7	80.4	83	83.4	83.8	**83.8**	0.72	0.73	0.74	0.74	0.75	0.83	0.80	0.81	0.82	**0.83**
DS15	70.0	71.33	70.7	72.8	73.7	79.4	80	82.4	83.8	**83.9**	0.59	0.72	0.71	0.70	0.72	0.80	0.81	0.81	0.82	**0.83**
DS17	71.0	72.33	72.77	71.8	70.7	79.4	82	80.4	81.8	**83.9**	0.70	0.71	0.70	0.72	0.70	0.81	0.78	0.80	0.81	**0.84**
DS18	70.0	71.33	70.7	72.8	73.7	79.4	80	82.4	83.8	**83.9**	0.59	0.72	0.71	0.70	0.72	0.80	0.81	0.81	0.82	**0.83**
DS18	70.33	72.33	73.77	72.8	70.77	81.4	83	83.4	83	**83.8**	0.70	0.71	0.79	0.78	0.70	0.72	0.78	0.80	0.81	**0.82**
DS19	70.33	73.33	71.77	72.8	74.77	83.4	83	83.4	84	**85**	0.70	0.71	0.79	0.78	0.70	0.72	0.78	0.80	0.81	**0.82**
DS20	71.0	72.33	71.77	78.8	73.7	82.4	82.8	83.4	83.8	**83.9**	0.70	0.71	0.70	0.72	0.70	0.81	0.78	0.80	0.81	**0.84**
DS21	72.0	74.33	73.7	75.8	74.8	83.4	84	83.4	84.8	**85.2**	0.70	0.73	0.73	0.73	0.73	0.83	0.84	0.82	0.84	**0.85**
DS22	71.0	74.33	73.77	78.8	70.7	80.4	83	82.4	83.8	**84.0**	0.72	0.73	0.73	0.74	0.72	0.82	0.83	0.82	0.82	**0.85**
DS23	72.0	74.33	73.7	75.8	74.8	83.4	84	83.4	84.8	**85.2**	0.70	0.73	0.73	0.73	0.73	0.83	0.84	0.82	0.84	**0.85**
DS24	75.0	76.0	78.7	78.8	70.7	81.4	83	82.4	84.0	**87.8**	0.70	0.77	0.76	0.78	0.74	0.83	0.83	0.85	0.84	**0.86**
DS25	72.3	75.3	75.7	75.9	74.8	84.4	83	85.4	84	**85.9**	0.72	0.76	0.79	0.78	0.79	0.76	0.80	0.82	0.82	**0.84**
DS26	73.0	75.3	77.07	75.8	74.8	83.4	84	83.4	84.8	**85.2**	0.70	0.73	0.73	0.73	0.73	0.83	0.84	0.82	0.84	**0.85**
DS27	72.0	74.33	73.7	75.8	74.8	83.4	84	83.4	84.8	**85.2**	0.70	0.73	0.73	0.76	0.73	0.83	0.84	0.82	0.84	**0.88**
DS28	71.0	70.33	79.77	79.8	71.77	80.4	81	81.4	82	**82.9**	0.71	0.70	0.71	0.70	0.71	0.80	0.79	0.81	0.82	**0.85**
DS29	76.3	77.33	78.77	78.8	70.77	81.4	82	82.4	81	**83**	0.70	0.71	0.79	0.78	0.70	0.72	0.78	0.80	0.81	**0.83**
DS30	77.0	74.33	73.7	75.8	74.8	83.4	84	83.4	84.8	**85.2**	0.70	0.73	0.73	0.73	0.73	0.83	0.84	0.82	0.84	**0.86**

Significant values are in [bold].

**Table 16:** Accuracy and F-measure using FR1 with 5% labeled dataset.

Accuracy											F-measure									
ID	J48	RF	NB	K-star	AIRS	Y-J48	Y-RF	Y-NB	Y-Kstar	Y-AIRS	J48	RF	NB	K-star	AIRS	Y-J48	Y-RF	Y-NB	Y-Kstar	Y-AIRS
DS1	57.3	54.3	55.5	57.7	51.5	**61.4**	60	**61.4**	60.5	**61.4**	0.51	0.50	0.51	0.51	0.52	**0.62**	0.60	**0.62**	0.60	**0.62**
DS2	50.8	52.3	57.5	57.7	50.5	61.4	62	62.4	61	**63**	0.50	0.51	0.59	0.57	0.50	0.62	0.61	0.60	0.61	**0.63**
DS3	51.0	50.3	59.5	59.7	51.5	60.4	61	61.4	62	**62.9**	0.51	0.50	0.51	0.50	0.51	0.60	0.59	0.61	0.62	**0.63**
DS4	51.0	52.3	57.5	57.7	50.5	59.4	62	60.4	61.7	**63.0**	0.50	0.51	0.50	0.52	0.50	0.61	0.59	0.60	0.61	**0.64**
DS5	50.0	51.3	50.5	52.7	53.5	59.4	60.8	62.4	63.7	**63.9**	0.59	0.52	0.51	0.50	0.52	0.60	0.61	0.61	0.62	**0.63**
DS6	50.0	52.3	52.5	53.7	52.7	61.4	62	61.4	62.7	**63.2**	0.57	0.50	0.52	0.51	0.51	0.61	0.62	0.60	0.63	**0.64**
DS7	51.0	52.3	57.5	57.7	50.5	59.4	60.1	60.4	61.7	**62.9**	0.50	0.51	0.50	0.52	0.50	0.61	0.57	0.60	0.61	**0.62**
DS8	51.3	52.3	57.5	57.7	50.5	61.4	62	62.4	61	**63**	0.50	0.51	0.59	0.57	0.50	0.59	0.57	0.60	0.61	**0.62**
DS9	53.3	52.3	57.5	57.7	50.5	61.4	62	62.4	61	**63**	0.50	0.51	0.59	0.57	0.50	0.59	0.59	0.60	0.61	**0.63**
DS10	50.0	51.3	52.5	52.7	53.5	59.4	60	62.4	63.7	**63.9**	0.59	0.52	0.51	0.50	0.52	0.60	0.61	0.61	0.62	**0.63**
DS11	51.0	52.3	57.5	57.7	50.5	59.4	62	60.4	61.7	**63.0**	0.50	0.51	0.50	0.52	0.50	0.61	0.57	0.60	0.61	**0.62**
DS12	51.0	50.3	59.5	59.7	51.5	60.4	61	61.4	62	**62.9**	0.51	0.50	0.51	0.50	0.51	0.60	0.59	0.61	0.62	**0.63**
DS13	50.3	52.3	57.5	57.7	50.5	61.4	62	62.4	61	**63**	0.50	0.51	0.59	0.57	0.50	0.62	0.57	0.60	0.61	**0.63**
DS14	51.0	50.3	59.5	59.7	51.5	60.4	61	61.4	62	**62.9**	0.51	0.50	0.51	0.50	0.51	0.60	0.59	0.61	0.62	**0.63**
DS15	50.0	51.3	50.5	52.7	53.5	59.4	60	62.4	63.7	**63.9**	0.59	0.52	0.51	0.50	0.52	0.60	0.61	0.61	0.62	**0.63**
DS16	51.0	53.3	57.5	57.7	50.5	61.4	62	60.4	61.7	**63.0**	0.50	0.51	0.50	0.52	0.50	0.61	0.57	0.60	0.61	**0.64**
DS17	50.0	51.3	50.5	52.7	53.5	59.4	60	62.4	63.7	**63.9**	0.59	0.52	0.51	0.50	0.52	0.60	0.61	0.61	0.62	**0.63**
DS18	53.4	54.3	57.5	57.7	50.5	61.4	62	62.4	61	**63**	0.50	0.51	0.59	0.57	0.50	0.59	0.60	0.60	0.61	**0.62**
DS19	50.3	52.3	57.5	57.7	50.5	61.4	62	62.4	61	**63**	0.50	0.51	0.59	0.57	0.50	0.58	0.59	0.60	0.61	**0.62**
DS20	55.0	52.3	57.5	57.7	53.5	59.4	62	60.4	61.7	**63.0**	0.50	0.51	0.50	0.52	0.50	0.61	0.57	0.60	0.61	**0.64**
DS21	50.3	52.3	57.5	57.7	51.6	61.4	62	62.4	61	**63**	0.50	0.51	0.59	0.57	0.50	0.62	0.57	0.60	0.61	**0.62**
DS22	51.0	53.4	57.5	57.7	50.5	59.4	62	60.4	61.7	**63.0**	0.50	0.51	0.50	0.52	0.50	0.61	0.57	0.60	0.61	**0.64**
DS23	50.0	51.3	50.5	52.7	53.5	59.4	60	62.4	63.7	**63.9**	0.59	0.52	0.51	0.50	0.52	0.60	0.61	0.61	0.62	**0.63**
DS24	51.0	52.3	57.5	57.7	50.5	59.4	62	60.4	61.7	**63.0**	0.50	0.51	0.50	0.52	0.50	0.61	0.57	0.60	0.61	**0.64**
DS25	54.3	53.4	57.5	57.7	50.5	61.4	62	62.4	61	**63**	0.50	0.51	0.59	0.57	0.50	0.52	0.57	0.60	0.61	**0.62**
DS26	51.0	52.3	57.5	57.7	50.5	59.4	62	60.4	61.7	**63.0**	0.50	0.51	0.50	0.52	0.50	0.61	0.57	0.60	0.61	**0.64**
DS27	50.3	52.3	57.5	57.7	50.5	61.4	62	62.4	61	**63**	0.50	0.51	0.59	0.57	0.50	0.59	0.59	0.60	0.61	**0.62**
DS28	51.0	54.3	59.5	59.7	51.5	60.4	61	61.4	62	**62.9**	0.51	0.50	0.51	0.50	0.51	0.60	0.59	0.61	0.62	**0.63**
DS29	50.3	52.3	57.5	57.7	50.5	61.4	62	62.4	61	**63**	0.50	0.51	0.59	0.57	0.50	0.59	0.57	0.60	0.61	**0.62**
DS30	50.0	52.3	52.5	53.7	52.7	61.4	62	61.4	62.7	**63.2**	0.57	0.50	0.52	0.51	0.51	0.61	0.62	0.60	0.63	**0.64**

Significant values are in [bold].

**Table 17:** Accuracy and F-measure using FR1 with 10% labeled dataset.

Accuracy											F-measure									
ID	J48	RF	NB	K-star	AIRS	Y-J48	Y-RF	Y-NB	Y-Kstar	Y-AIRS	J48	RF	NB	K-star	AIRS	Y-J48	Y-RF	Y-NB	Y-Kstar	Y-AIRS
DS1	59.3	55.3	55.5	56.5	53.5	**63.4**	63	**63.4**	61.5	**63.4**	0.53	0.52	0.53	0.53	0.54	**0.63**	0.61	**0.63**	0.61	**0.63**
DS2	52.3	53.3	50.5	50.6	52.5	62.4	63	63.4	61.9	**63.6**	0.52	0.53	0.50	0.51	0.52	0.55	0.59	0.62	0.63	**0.64**
DS3	52.0	52.3	50.5	50.6	52.5	62.4	63	63.4	63	**64.9**	0.52	0.52	0.53	0.52	0.53	0.62	0.61	0.62	0.63	**0.64**
DS4	52.0	53.3	51.5	52.6	52.5	60.4	63	63.4	63.6	**63.6**	0.52	0.53	0.54	0.54	0.55	0.63	0.60	0.61	0.62	**0.63**
DS5	53.0	54.3	54.5	54.6	55.5	60.4	62	63.4	64.6	**64.9**	0.50	0.53	0.54	0.53	0.53	0.62	0.63	0.63	0.62	**0.64**
DS6	52.0	54.3	53.5	55.6	54.6	63.4	64	63.4	64.6	**65.2**	0.50	0.53	0.53	0.53	0.53	0.63	0.64	0.62	0.64	**0.65**
DS7	52.0	53.3	51.5	52.6	52.5	60.4	63	63.4	63.6	**63.6**	0.52	0.53	0.54	0.54	0.55	0.63	0.60	0.61	0.62	**0.63**
DS8	50.3	52.3	51.5	52.6	54.5	63.4	62	64.4	63	**64**	0.50	0.52	0.52	0.51	0.52	0.53	0.55	0.60	0.62	**0.64**
DS9	52.0	53.3	51.5	52.6	52.5	60.4	63	63.4	63.6	**63.6**	0.52	0.53	0.54	0.54	0.55	0.63	0.60	0.61	0.62	**0.63**
DS10	52.0	53.3	51.5	52.6	52.5	60.4	63	63.4	61.6	**63.6**	0.52	0.53	0.54	0.54	0.55	0.63	0.60	0.61	0.62	**0.63**
DS11	52.0	53.3	51.5	52.6	52.5	60.4	63	63.4	62.6	**63.6**	0.52	0.53	0.54	0.54	0.55	0.63	0.60	0.61	0.62	**0.63**
DS12	51.0	50.3	51.5	50.6	51.5	60.4	61	61.4	62	**62.9**	0.51	0.50	0.51	0.50	0.51	0.60	0.59	0.61	0.62	**0.63**
DS13	52.3	54.3	51.5	52.6	53.5	63.4	64	63.4	63	**64.6**	0.50	0.51	0.59	0.56	0.50	0.52	0.56	0.60	0.61	**0.62**
DS14	52.0	53.3	51.5	52.6	52.5	60.4	63	63.4	63.6	**63.6**	0.52	0.53	0.54	0.54	0.55	0.63	0.60	0.61	0.62	**0.63**
DS15	50.0	51.3	50.5	52.6	53.5	59.4	60	62.4	63.6	**63.9**	0.59	0.52	0.51	0.50	0.52	0.60	0.61	0.61	0.62	**0.63**
DS15	51.0	52.3	52.5	51.6	50.5	59.4	62	60.4	61.6	**63.9**	0.50	0.51	0.50	0.52	0.50	0.61	0.56	0.60	0.61	**0.64**
DS16	50.0	51.3	50.5	52.6	53.5	59.4	60	62.4	63.6	**63.9**	0.59	0.52	0.51	0.50	0.52	0.60	0.61	0.61	0.62	**0.63**
DS18	50.3	52.3	53.5	52.6	50.5	61.4	63	63.4	63	**63.6**	0.50	0.51	0.59	0.56	0.50	0.52	0.56	0.60	0.61	**0.62**
DS19	50.3	53.3	51.5	52.6	54.5	63.4	63	63.4	64	**65**	0.50	0.51	0.59	0.56	0.50	0.52	0.56	0.60	0.61	**0.62**
DS20	51.0	52.3	51.5	56.6	53.5	62.4	62.6	63.4	63.6	**63.9**	0.50	0.51	0.50	0.52	0.50	0.61	0.56	0.60	0.61	**0.64**
DS21	52.0	54.3	53.5	55.6	54.6	63.4	64	63.4	64.6	**65.2**	0.50	0.53	0.53	0.53	0.53	0.63	0.64	0.62	0.64	**0.65**
DS22	51.0	54.3	53.5	56.6	50.5	60.4	63	62.4	63.6	**64.0**	0.52	0.53	0.53	0.54	0.52	0.62	0.63	0.62	0.62	**0.65**
DS23	52.0	54.3	53.5	55.6	54.6	63.4	64	63.4	64.6	**65.2**	0.50	0.53	0.53	0.53	0.53	0.63	0.64	0.62	0.64	**0.65**
DS24	54.0	55.3	53.5	56.6	50.5	61.4	63	62.4	63.6	**64.6**	0.53	0.54	0.55	0.55	0.54	0.63	0.62	0.63	0.64	**0.65**
DS25	50.3	52.3	51.5	50.6	50.5	61.4	62	62.4	61	**63**	0.50	0.51	0.59	0.56	0.50	0.52	0.56	0.60	0.61	**0.62**
DS26	52.0	54.3	53.5	55.6	54.6	63.4	64	63.4	64.6	**65.2**	0.50	0.53	0.53	0.53	0.53	0.63	0.64	0.62	0.64	**0.65**
DS27	52.0	54.3	53.5	55.6	54.6	63.4	64	63.4	64.6	**65.2**	0.50	0.53	0.53	0.53	0.53	0.63	0.64	0.62	0.64	**0.65**
DS28	51.0	50.3	59.5	59.6	51.5	60.4	61	61.4	62	**62.9**	0.51	0.50	0.51	0.50	0.51	0.60	0.59	0.61	0.62	**0.63**
DS29	50.3	52.3	56.5	56.6	50.5	61.4	62	62.4	61	**63**	0.50	0.51	0.59	0.56	0.50	0.52	0.56	0.60	0.61	**0.62**
DS30	52.0	54.3	53.5	55.6	54.6	63.4	64	63.4	64.6	**65.2**	0.50	0.53	0.53	0.53	0.53	0.63	0.64	0.62	0.64	**0.65**

Significant values are in [bold].

**Table 18:** Accuracy and F-measure using FR1 with optimal number of labeled dataset (i.e., 60%).

Accuracy											F-measure									
ID	J48	RF	NB	K-star	AIRS	Y-J48	Y-RF	Y-NB	Y-Kstar	Y-AIRS	J48	RF	NB	K-star	AIRS	Y-J48	Y-RF	Y-NB	Y-Kstar	Y-AIRS
DS1	73.3	72.3	77.6	79.7	78.6	**79.4**	78.0	**79.4**	75.6	**79.4**	0.72	0.73	0.75	0.75	0.76	**0.79**	0.78	**0.79**	0.76	**0.79**
DS2	77.3	75.3	75.7	75.8	76.7	83.4	83.9	82.4	83.9	**85.8**	0.73	0.73	0.73	0.71	0.75	0.78	0.80	0.82	0.83	**0.83**
DS3	72.0	72.3	70.7	70.8	72.7	82.4	83	83.4	83	**84.9**	0.72	0.72	0.73	0.72	0.73	0.82	0.81	0.82	0.83	**0.84**
DS4	72.0	73.3	71.8	72.8	72.7	80.4	83	83.4	83.8	**83.8**	0.72	0.73	0.74	0.74	0.75	0.83	0.80	0.81	0.82	**0.83**
DS5	73.0	74.3	74.7	74.8	75.7	80.4	82	83.4	84.8	**84.9**	0.70	0.73	0.74	0.73	0.73	0.82	0.83	0.83	0.82	**0.84**
DS6	72.0	74.3	77.7	75.8	74.8	83.4	84	83.4	84.8	**85.2**	0.70	0.73	0.73	0.73	0.73	0.83	0.84	0.82	0.84	**0.85**
DS7	72.0	73.3	74.7	72.8	72.7	82.4	83	83.4	83.8	**83.8**	0.72	0.73	0.74	0.74	0.75	0.83	0.80	0.81	0.82	**0.83**
DS8	70.3	72.3	71.7	72.8	74.7	83.4	83	84.4	83	**84.7**	0.70	0.72	0.72	0.71	0.72	0.73	0.75	0.80	0.82	**0.84**
DS9	72.0	72.3	72.7	72.8	72.7	80.4	83	83.4	83.8	**83.8**	0.72	0.75	0.74	0.74	0.75	0.83	0.80	0.81	0.82	**0.83**
DS10	72.0	71.3	71.9	72.8	72.7	80.4	83	83.4	83.8	**83.8**	0.73	0.73	0.74	0.74	0.75	0.83	0.80	0.81	0.82	**0.83**
DS11	72.0	74.3	74.7	72.8	72.7	81.4	83	83.4	83.8	**83.8**	0.72	0.74	0.74	0.74	0.75	0.83	0.80	0.81	0.82	**0.83**
DS12	71.0	70.3	71.7	70.8	71.7	80.4	81	81.4	82	**82.9**	0.71	0.72	0.71	0.70	0.71	0.80	0.79	0.81	0.82	**0.83**
DS13	72.3	74.3	71.7	72.8	73.7	83.4	84	83.4	83	**84.8**	0.70	0.73	0.79	0.78	0.70	0.72	0.78	0.80	0.81	**0.82**
DS14	72.0	73.3	71.7	72.8	72.7	80.4	83	83.4	83.8	**83.8**	0.72	0.73	0.74	0.74	0.75	0.83	0.80	0.81	0.82	**0.83**
DS15	70.0	71.3	70.7	72.8	73.7	79.4	80	82.4	83.8	**83.9**	0.79	0.72	0.71	0.70	0.72	0.80	0.81	0.81	0.82	**0.83**
DS17	71.0	72.3	72.7	71.8	70.7	79.4	82	80.4	81.8	**83.9**	0.70	0.71	0.70	0.72	0.70	0.81	0.78	0.80	0.81	**0.84**
DS18	70.0	71.3	70.7	72.8	73.7	79.4	82	82.4	83.8	**83.9**	0.79	0.72	0.71	0.70	0.72	0.80	0.81	0.81	0.82	**0.83**
DS19	70.3	73.3	71.7	72.8	74.7	83.4	83	83.4	84	**85**	0.70	0.71	0.79	0.78	0.70	0.72	0.78	0.80	0.81	**0.82**
DS20	71.0	72.3	71.7	78.8	73.7	82.4	82.8	83.4	83.8	**88.9**	0.70	0.71	0.70	0.72	0.70	0.81	0.78	0.80	0.81	**0.84**
DS21	72.0	74.3	73.7	75.8	74.8	83.4	84	83.4	84.8	**87.2**	0.70	0.73	0.73	0.73	0.73	0.83	0.84	0.82	0.84	**0.85**
DS22	71.0	74.3	73.7	78.8	70.7	83.4	83	82.4	83.8	**84.0**	0.72	0.73	0.73	0.74	0.72	0.82	0.83	0.82	0.82	**0.85**
DS23	72.0	74.3	73.7	75.8	74.8	83.4	84	83.4	84.8	**85.2**	0.70	0.73	0.73	0.73	0.73	0.83	0.84	0.82	0.84	**0.85**
DS24	74.0	75.3	73.7	78.8	70.7	81.4	83	82.4	83.8	**84.8**	0.73	0.74	0.75	0.77	0.74	0.83	0.82	0.83	0.84	**0.85**
DS25	70.3	72.3	71.7	70.8	70.7	81.4	82	82.4	81	**86.3**	0.70	0.71	0.79	0.78	0.70	0.72	0.78	0.80	0.81	**0.82**
DS26	72.0	74.3	73.7	75.8	74.8	83.4	84	83.4	84.8	**85.2**	0.70	0.73	0.73	0.73	0.73	0.83	0.84	0.82	0.84	**0.85**
DS27	72.0	74.3	73.7	75.8	74.8	83.4	84	83.4	84.8	**87.2**	0.70	0.73	0.73	0.73	0.73	0.83	0.84	0.82	0.84	**0.85**
DS28	71.0	70.3	69.7	69.8	71.77	80.4	81	81.4	82	**82.9**	0.71	0.70	0.68	0.67	0.71	0.80	0.79	0.81	0.82	**0.83**
DS29	70.3	72.3	68.7	68.8	70.77	81.4	82	82.4	81	**83.9**	0.70	0.71	0.69	0.68	0.70	0.72	0.78	0.80	0.81	**0.82**
DS30	72.0	74.3	73.7	75.8	74.8	83.4	84	83.4	84.8	**86.2**	0.70	0.73	0.73	0.73	0.73	0.83	0.84	0.82	0.84	**0.85**

Significant values are in [bold].

**Table 19:** Accuracy and F-measure using FR1 with 100% labeled dataset.

Accuracy											F-measure									
ID	J48	RF	NB	K-star	AIRS	Y-J48	Y-RF	Y-NB	Y-Kstar	Y-AIRS	J48	RF	NB	K-star	AIRS	Y-J48	Y-RF	Y-NB	Y-Kstar	Y-AIRS
DS1	73.3	72.3	77.6	79.7	78.6	**79.4**	78.0	**79.4**	75.6	**79.4**	0.72	0.73	0.75	0.75	0.76	**0.79**	0.78	**0.79**	0.76	**0.79**
DS2	77.3	75.3	75.7	75.8	76.7	83.4	83.9	82.4	83.9	**85.8**	0.73	0.73	0.73	0.71	0.75	0.78	0.8	0.82	0.83	**0.83**
DS3	72.0	72.3	70.7	70.8	72.7	82.4	83	83.4	83	**84.9**	0.72	0.72	0.73	0.72	0.73	0.82	0.81	0.82	0.83	**0.84**
DS4	72.0	73.3	71.8	72.8	72.7	80.4	83	83.4	83.8	**83.8**	0.72	0.73	0.74	0.74	0.75	0.83	0.80	0.81	0.82	**0.83**
DS5	73.0	74.3	74.7	74.8	75.7	80.4	82	83.4	84.8	**84.9**	0.70	0.73	0.74	0.73	0.73	0.82	0.83	0.83	0.82	**0.84**
DS6	72.0	74.3	77.7	75.8	74.8	83.4	84	83.4	84.8	**85.2**	0.70	0.73	0.73	0.73	0.73	0.83	0.84	0.82	0.84	**0.85**
DS7	72.0	73.3	74.7	72.8	72.7	82.4	83	83.4	83.8	**83.8**	0.72	0.73	0.74	0.74	0.75	0.83	0.80	0.81	0.82	**0.83**
DS8	70.3	72.3	71.7	72.8	74.7	83.4	83	84.4	83	**84.7**	0.70	0.72	0.72	0.71	0.72	0.73	0.75	0.80	0.82	**0.84**
DS9	72.0	72.3	72.7	72.8	72.7	80.4	83	83.4	83.8	**83.8**	0.72	0.75	0.74	0.74	0.75	0.83	0.80	0.81	0.82	**0.83**
DS10	72.0	71.3	71.9	72.8	72.7	80.4	83	83.4	83.8	**83.8**	0.73	0.73	0.74	0.74	0.75	0.83	0.80	0.81	0.82	**0.83**
DS11	72.0	74.3	74.7	72.8	72.7	81.4	83	83.4	83.8	**83.8**	0.72	0.74	0.74	0.74	0.75	0.83	0.80	0.81	0.82	**0.83**
DS12	71.0	70.3	71.7	70.8	71.7	80.4	81	81.4	82	**82.9**	0.71	0.72	0.71	0.70	0.71	0.80	0.79	0.81	0.82	**0.83**
DS13	72.3	74.3	71.7	72.8	73.7	83.4	84	83.4	83	**84.8**	0.70	0.73	0.79	0.78	0.70	0.72	0.78	0.80	0.81	**0.82**
DS14	72.0	73.3	71.7	72.8	72.7	80.4	83	83.4	83.8	**83.8**	0.72	0.73	0.74	0.74	0.75	0.83	0.80	0.81	0.82	**0.83**
DS15	70.0	71.3	70.7	72.8	73.7	79.4	80	82.4	83.8	**83.9**	0.79	0.72	0.71	0.70	0.72	0.80	0.81	0.81	0.82	**0.83**
DS17	71.0	72.3	72.7	71.8	70.7	79.4	82	80.4	81.8	**83.9**	0.70	0.71	0.70	0.72	0.70	0.81	0.78	0.80	0.81	**0.84**
DS18	70.0	71.3	70.7	72.8	73.7	79.4	82	82.4	83.8	**83.9**	0.79	0.72	0.71	0.70	0.72	0.80	0.81	0.81	0.82	**0.83**
DS18	70.3	72.3	73.7	72.8	70.7	81.4	83	83.4	83	**83.8**	0.70	0.71	0.79	0.78	0.70	0.72	0.78	0.80	0.81	**0.82**
DS19	70.3	73.3	71.7	72.8	74.7	83.4	83	83.4	84	**85**	0.70	0.71	0.79	0.78	0.70	0.72	0.78	0.80	0.81	**0.82**
DS20	71.0	72.3	71.7	78.8	73.7	82.4	82.8	83.4	83.8	**88.9**	0.70	0.71	0.70	0.72	0.70	0.81	0.78	0.80	0.81	**0.84**
DS21	72.0	74.3	73.7	75.8	74.8	83.4	84	83.4	84.8	**87.2**	0.70	0.73	0.73	0.73	0.73	0.83	0.84	0.82	0.84	**0.85**
DS22	71.0	74.3	73.7	78.8	70.7	83.4	83	82.4	83.8	**84.0**	0.72	0.73	0.73	0.74	0.72	0.82	0.83	0.82	0.82	**0.85**
DS23	72.0	74.3	73.7	75.8	74.8	83.4	84	83.4	84.8	**85.2**	0.70	0.73	0.73	0.73	0.73	0.83	0.84	0.82	0.84	**0.85**
DS24	74.0	75.3	73.7	78.8	70.7	81.4	83	82.4	83.8	**84.8**	0.73	0.74	0.75	0.77	0.74	0.83	0.82	0.83	0.84	**0.85**
DS25	70.3	72.3	71.7	70.8	70.7	81.4	82	82.4	81	**86.3**	0.70	0.71	0.79	0.78	0.70	0.72	0.78	0.80	0.81	**0.82**
DS26	72.0	74.33	73.7	75.8	74.8	83.4	84	83.4	84.8	**85.2**	0.70	0.73	0.73	0.73	0.73	0.83	0.84	0.82	0.84	**0.85**
DS27	72.0	74.3	73.7	75.8	74.8	83.4	84	83.4	84.8	**87.2**	0.70	0.73	0.73	0.73	0.73	0.83	0.84	0.82	0.84	**0.85**
DS28	71.0	70.3	69.7	69.8	71.77	80.4	81	81.4	82	**82.9**	0.71	0.70	0.68	0.67	0.71	0.80	0.79	0.81	0.82	**0.83**
DS29	70.3	72.3	68.7	68.8	70.77	81.4	82	82.4	81	**83.9**	0.70	0.71	0.69	0.68	0.70	0.72	0.78	0.80	0.81	**0.82**
DS30	72.0	74.3	73.7	75.8	74.8	83.4	84	83.4	84.8	**86.2**	0.70	0.73	0.73	0.73	0.73	0.83	0.84	0.82	0.84	**0.85**

Significant values are in [bold].

**Table 20:** Accuracy and F-measure using FR2 with 5% labeled dataset.

Accuracy											F-measure									
ID	J48	RF	NB	K-star	AIRS	Y-J48	Y-RF	Y-NB	Y-Kstar	Y-AIRS	J48	RF	NB	K-star	AIRS	Y-J48	Y-RF	Y-NB	Y-Kstar	Y-AIRS
DS1	56.3	58.3	58.3	58.7	58.5	**62.4**	60	**62.4**	60.5	**62.4**	0.56	0.53	0.55	0.56	0.54	**0.61**	0.60	**0.61**	0.60	**0.61**
DS2	53.8	56.3	56.5	57.8	56.5	61.4	62.8	61.4	62.8	**62.8**	0.52	0.51	0.59	0.57	0.50	0.62	0.61	0.60	0.61	**0.64**
DS3	56.0	57.3	58.5	59.7	57.5	61.4	61.4	62.4	62	**64.9**	0.51	0.52	0.53	0.50	0.53	0.61	0.62	0.61	0.62	**0.64**
DS4	58.0	58.3	57.5	59.7	59.8	63.4	62.8	62.4	63.7	**65.3**	0.50	0.51	0.50	0.52	0.53	0.61	0.61	0.60	0.61	**0.64**
DS5	57.8	57.3	58.8	58.9	59.5	63.4	62.8	64.4	63.7	**65**	0.59	0.52	0.53	0.51	0.52	0.60	0.61	0.62	0.62	**0.64**
DS6	53.0	53.3	54.5	58.7	56.7	61.4	62.8	62.4	62.7	**63.8**	0.57	0.53	0.52	0.53	0.51	0.61	0.62	0.60	0.63	**0.65**
DS7	53.0	52.3	57.5	57.7	50.5	59.4	60.1	60.4	61.7	**63.9**	0.50	0.51	0.50	0.52	0.50	0.61	0.57	0.60	0.61	**0.62**
DS8	53.3	52.3	57.5	57.7	54.5	61.4	62	62.4	61	**63.3**	0.50	0.51	0.59	0.57	0.50	0.59	0.57	0.60	0.61	**0.62**
DS9	53.3	52.3	57.5	57.7	52.5	61.4	62	62.4	61	**64.1**	0.50	0.51	0.59	0.57	0.50	0.59	0.59	0.60	0.61	**0.63**
DS10	56.0	56.3	58.5	52.7	53.5	61.4	60.9	62.4	63.7	**63.9**	0.59	0.52	0.51	0.50	0.52	0.60	0.61	0.61	0.62	**0.63**
DS11	53.0	52.3	57.5	57.7	50.5	61.4	62	60.4	62.7	**63.4**	0.50	0.51	0.50	0.52	0.50	0.61	0.57	0.60	0.61	**0.62**
DS12	54.0	53.3	59.5	59.7	51.5	60.4	61	61.4	62	**61.9**	0.51	0.50	0.51	0.50	0.51	0.60	0.59	0.61	0.62	**0.63**
DS13	54.3	52.3	57.5	57.7	50.5	61.4	62	62.4	61	**63.4**	0.50	0.51	0.59	0.57	0.50	0.62	0.57	0.60	0.61	**0.63**
DS14	55.0	57.3	59.5	59.7	54.5	62.4	61	61.4	62	**63.9**	0.51	0.50	0.51	0.50	0.51	0.60	0.59	0.61	0.62	**0.63**
DS15	53.9	53.3	50.5	52.7	53.5	59.4	61	62.4	63.7	**64.9**	0.59	0.52	0.51	0.50	0.52	0.60	0.61	0.61	0.62	**0.63**
DS16	54.3	53.3	57.5	57.7	53.5	61.4	62	60.4	61.7	**63.9**	0.50	0.51	0.50	0.52	0.50	0.61	0.57	0.60	0.61	**0.64**
DS17	53.0	51.3	54.5	52.7	53.5	59.4	60	62.4	63.7	**63.9**	0.59	0.52	0.51	0.50	0.52	0.60	0.61	0.61	0.62	**0.63**
DS18	53.4	54.3	57.5	57.7	50.5	61.4	62	62.4	61	**64.2**	0.50	0.51	0.59	0.57	0.50	0.59	0.60	0.60	0.61	**0.62**
DS19	54.3	52.3	57.5	57.7	50.5	61.4	62	62.4	61	**63.7**	0.50	0.51	0.59	0.57	0.50	0.58	0.59	0.60	0.61	**0.62**
DS20	56.0	52.3	57.5	59.7	53.5	59.4	62	60.4	61.7	**64.8**	0.50	0.51	0.50	0.52	0.50	0.61	0.57	0.60	0.61	**0.64**
DS21	53.3	52.3	57.5	57.7	51.6	61.4	62	62.4	61	**63.9**	0.50	0.51	0.59	0.57	0.50	0.62	0.57	0.60	0.61	**0.62**
DS22	51.0	53.4	57.5	57.7	50.5	59.4	62	60.4	61.7	**64.7**	0.50	0.51	0.50	0.52	0.50	0.61	0.57	0.60	0.61	**0.64**
DS23	55.0	51.3	50.5	52.7	53.5	59.4	60	62.4	63.7	**64.9**	0.59	0.52	0.51	0.50	0.52	0.60	0.61	0.61	0.62	**0.63**
DS24	54.0	54.3	57.5	57.7	50.5	59.4	62	60.4	61.7	**64.7**	0.50	0.51	0.50	0.52	0.50	0.61	0.57	0.60	0.61	**0.64**
DS25	54.3	53.4	57.5	57.7	50.5	61.4	62	62.4	61	**64.2**	0.50	0.51	0.59	0.57	0.50	0.52	0.57	0.60	0.61	**0.62**
DS26	54.0	54.3	57.5	57.7	50.5	59.4	62	60.4	61.7	**63.7**	0.50	0.51	0.50	0.52	0.50	0.61	0.57	0.60	0.61	**0.64**
DS27	53.3	52.3	57.5	57.7	50.5	61.4	62	62.4	61	**63.8**	0.50	0.51	0.59	0.57	0.50	0.59	0.59	0.60	0.61	**0.62**
DS28	53.0	54.3	59.5	59.7	51.5	60.4	61	61.4	62	**63.9**	0.51	0.50	0.51	0.50	0.51	0.60	0.59	0.61	0.62	**0.63**
DS29	54.3	52.3	57.5	57.7	50.5	61.4	62	62.4	61	**63.2**	0.50	0.51	0.59	0.57	0.50	0.59	0.57	0.60	0.61	**0.62**
DS30	54.0	52.3	52.5	53.7	52.7	61.4	62	61.4	62.7	**63.2**	0.57	0.50	0.52	0.51	0.51	0.61	0.62	0.60	0.63	**0.64**

Significant values are in [bold].

**Table 21:** Accuracy and F-measure using FR2 with 10% labeled dataset.

Accuracy											F-measure									
ID	J48	RF	NB	K-star	AIRS	Y-J48	Y-RF	Y-NB	Y-Kstar	Y-AIRS	J48	RF	NB	K-star	AIRS	Y-J48	Y-RF	Y-NB	Y-Kstar	Y-AIRS
DS1	57.9	57.8	57.5	58.5	53.5	**67.4**	65	**67.4**	65.5	**67.4**	0.52	0.52	0.53	0.53	0.54	**0.64**	0.62	**0.64**	0.62	**0.64**
DS2	52.8	53.8	53.5	53.6	52.7	62.4	63	63.4	62.9	**64.0**	0.52	0.53	0.50	0.51	0.52	0.55	0.59	0.62	0.63	**0.64**
DS3	52.8	52.3	52.5	52.6	52.5	62.4	63	63.4	63	**64.9**	0.52	0.52	0.53	0.52	0.53	0.62	0.61	0.62	0.63	**0.64**
DS4	52.0	53.3	53.5	53.6	53.5	60.4	63	63.4	63.6	**64.6**	0.52	0.53	0.54	0.54	0.55	0.63	0.60	0.61	0.62	**0.63**
DS5	53.0	54.3	54.5	55.6	55.5	60.4	62	63.4	64.6	**65.9**	0.50	0.53	0.54	0.53	0.53	0.62	0.63	0.63	0.62	**0.64**
DS6	52.0	54.3	53.5	55.6	54.6	63.4	64	63.4	64.6	**65.2**	0.50	0.53	0.53	0.53	0.53	0.63	0.64	0.62	0.64	**0.65**
DS7	56.0	56.3	56.5	55.6	52.5	60.4	63	63.4	63.6	**63.6**	0.52	0.53	0.54	0.54	0.55	0.63	0.60	0.61	0.62	**0.63**
DS8	56.3	52.3	53.5	52.6	54.5	63.4	62	64.4	63	**64**	0.50	0.52	0.52	0.51	0.52	0.53	0.55	0.60	0.62	**0.64**
DS9	57.6	54.3	53.5	52.6	52.5	60.4	63	63.4	63.6	**62.6**	0.52	0.53	0.54	0.54	0.55	0.63	0.60	0.61	0.62	**0.63**
DS10	52.0	53.3	51.5	52.6	52.5	60.4	63	62.4	62.6	**63.6**	0.52	0.53	0.54	0.54	0.55	0.63	0.60	0.61	0.62	**0.63**
DS11	52.8	52.3	51.5	52.6	52.5	62.4	63.1	62.4	62.4	**62.6**	0.52	0.53	0.54	0.54	0.55	0.63	0.60	0.61	0.62	**0.63**
DS12	51.0	50.3	51.5	50.6	51.5	61.4	61.1	61.4	62	**62.9**	0.51	0.50	0.51	0.50	0.51	0.60	0.59	0.61	0.62	**0.63**
DS13	52.3	54.3	51.5	52.6	53.5	63.4	64.1	63.4	63.2	**64.6**	0.50	0.51	0.59	0.56	0.50	0.52	0.56	0.60	0.61	**0.62**
DS14	52.0	53.3	51.5	52.6	52.5	60.4	63	63.4	63.6	**64.0**	0.52	0.53	0.54	0.54	0.55	0.63	0.60	0.61	0.62	**0.63**
DS15	54.0	51.3	50.5	52.6	53.5	59.4	60	62.4	63.6	**64.9**	0.59	0.52	0.51	0.50	0.52	0.60	0.61	0.61	0.62	**0.63**
DS15	53.0	52.3	52.5	51.6	50.5	59.4	62	60.4	61.6	**62.9**	0.50	0.51	0.50	0.52	0.50	0.61	0.56	0.60	0.61	**0.64**
DS16	52.5	51.3	50.5	52.6	53.5	59.4	60	62.4	63.6	**64.9**	0.59	0.52	0.51	0.50	0.52	0.60	0.61	0.61	0.62	**0.63**
DS18	53.3	52.3	53.5	52.6	50.5	61.4	63	63.4	63.4	**64.6**	0.50	0.51	0.59	0.56	0.50	0.52	0.56	0.60	0.61	**0.62**
DS19	50.3	53.3	51.5	52.6	54.5	63.4	63.5	63.4	64.3	**65.1**	0.50	0.51	0.59	0.56	0.50	0.52	0.56	0.60	0.61	**0.62**
DS20	51.8	52.3	51.5	56.6	53.5	62.4	62.6	63.4	63.6	**63.9**	0.50	0.51	0.50	0.52	0.50	0.61	0.56	0.60	0.61	**0.64**
DS21	52.4	54.3	53.5	55.6	54.6	63.4	64	63.4	64.6	**65.2**	0.50	0.53	0.53	0.53	0.53	0.63	0.64	0.62	0.64	**0.65**
DS22	53.7	54.3	53.5	56.6	50.5	62.4	63.3	62.4	63.6	**64.0**	0.52	0.53	0.53	0.54	0.52	0.62	0.63	0.62	0.62	**0.65**
DS23	52.6	54.3	53.5	55.6	54.6	63.4	64.1	63.4	64.6	**65.0**	0.50	0.53	0.53	0.53	0.53	0.63	0.64	0.62	0.64	**0.65**
DS24	54.0	55.3	53.5	56.6	50.5	61.4	63	61.4	63.6	**64.6**	0.53	0.54	0.55	0.55	0.54	0.63	0.62	0.63	0.64	**0.65**
DS25	52.3	52.3	51.5	50.6	50.5	61.4	62	62.4	61	**63.2**	0.50	0.51	0.59	0.56	0.50	0.52	0.56	0.60	0.61	**0.62**
DS26	52.8	54.3	53.5	55.6	54.6	63.4	64	63.4	64.6	**65.2**	0.50	0.53	0.53	0.53	0.53	0.63	0.64	0.62	0.64	**0.65**
DS27	53.5	54.3	53.5	55.6	54.6	63.4	64	63.4	64.6	**65.2**	0.50	0.53	0.53	0.53	0.53	0.63	0.64	0.62	0.64	**0.65**
DS28	53.0	50.3	59.5	59.6	51.5	60.4	61	61.4	62	**62.9**	0.51	0.50	0.51	0.50	0.51	0.60	0.59	0.61	0.62	**0.63**
DS29	54.3	52.3	56.5	56.6	50.5	61.4	62	62.4	61	**63.9**	0.50	0.51	0.59	0.56	0.50	0.52	0.56	0.60	0.61	**0.62**
DS30	55.3	54.3	53.5	55.6	54.6	63.4	64	63.4	64.6	**65.6**	0.50	0.53	0.53	0.53	0.53	0.63	0.64	0.62	0.64	**0.65**

Significant values are in [bold].

**Table 22:** Accuracy and F-measure using FR2 with optimal number of labeled dataset (i.e., 60%)

Accuracy											F-measure									
ID	J48	RF	NB	K-star	AIRS	Y-J48	Y-RF	Y-NB	Y-Kstar	Y-AIRS	J48	RF	NB	K-star	AIRS	Y-J48	Y-RF	Y-NB	Y-Kstar	Y-AIRS
DS1	78.3	79.6	78.6	79.7	79.2	**88.4**	83.0	**88.4**	85.2	**88.4**	0.73	0.75	0.78	0.78	0.79	**0.83**	0.81	**0.83**	0.82	**0.83**
DS2	79.0	79.8	79.7	80.8	82.7	85.4	87.3	85.4	84.9	**87.8**	0.79	0.78	0.75	0.79	0.80	0.82	0.81	0.83	0.83	**0.85**
DS3	72.0	72.3	70.7	70.8	72.7	82.4	83	83.4	83	**84.9**	0.72	0.72	0.73	0.72	0.73	0.82	0.81	0.82	0.83	**0.84**
DS4	75.0	73.3	71.8	72.8	72.7	80.4	83	83.4	83.8	**83.8**	0.72	0.73	0.74	0.74	0.75	0.83	0.80	0.81	0.82	**0.83**
DS5	78.0	74.33	74.7	74.8	75.7	80.4	82	83.4	84.8	**84.9**	0.70	0.73	0.74	0.73	0.73	0.82	0.83	0.83	0.82	**0.84**
DS6	74.3	74.33	77.7	75.8	74.8	83.4	84	83.4	84.8	**85.2**	0.70	0.73	0.73	0.73	0.73	0.83	0.84	0.82	0.84	**0.85**
DS7	72.7	73.6	74.7	72.8	72.7	82.4	83	83.4	83.8	**83.8**	0.72	0.73	0.74	0.74	0.75	0.83	0.80	0.81	0.82	**0.85**
DS8	74.3	71.33	72.7	71.8	74.7	80.4	83	82.4	83	**83.7**	0.72	0.73	0.73	0.74	0.73	0.84	0.80	0.80	0.82	**0.84**
DS9	74.3	73.3	74.7	73.8	74.7	80.4	83	83.4	83.8	**85.8**	0.72	0.75	0.74	0.74	0.75	0.83	0.80	0.81	0.82	**0.83**
DS10	76.0	75.3	73.9	74.8	74.7	80.4	83	82.4	84.8	**87.8**	0.73	0.75	0.76	0.77	0.75	0.83	0.82	0.84	0.84	**0.85**
DS11	75.0	74.9	77.7	76.8	79.7	82.4	83.7	85.4	85.8	**86.8**	0.72	0.77	0.77	0.76	0.77	0.83	0.82	0.83	0.82	**0.84**
DS12	72.0	77.3	76.7	77.8	77.7	82.4	83	83.4	83	**83.9**	0.76	0.75	0.74	0.73	0.74	0.81	0.80	0.82	0.83	**0.84**
DS13	78.3	75.3	76.7	75.8	76.7	83.4	84	83.4	83	**84.8**	0.70	0.73	0.79	0.78	0.70	0.80	0.81	0.80	0.81	**0.82**
DS14	74.0	75.3	76.7	77.8	76.7	81.4	83	83.4	81.8	**83.8**	0.72	0.74	0.75	0.75	0.77	0.83	0.80	0.81	0.82	**0.84**
DS15	76.0	76.3	76.7	77.8	78.7	80.4	80	82.4	83.8	**83.9**	0.79	0.72	0.71	0.70	0.72	0.80	0.81	0.81	0.82	**0.83**
DS17	73.0	77.3	78.7	79.8	79.7	82.4	82.8	83.4	81.8	**83.9**	0.70	0.71	0.70	0.72	0.70	0.81	0.78	0.80	0.81	**0.84**
DS18	76.0	74.3	78.7	76.8	79.7	80.4	82.3	83.4	83.8	**84.1**	0.79	0.72	0.71	0.70	0.72	0.80	0.81	0.81	0.82	**0.83**
DS18	79.3	75.3	76.7	74.8	77.8	81.4	83	83.4	83	**83.8**	0.70	0.71	0.75	0.76	0.70	0.80	0.82	0.80	0.81	**0.82**
DS19	77.8	77.3	78.8	79.8	78.0	83.4	83	83.4	84	**85**	0.70	0.71	0.79	0.78	0.70	0.80	0.82	0.80	0.81	**0.82**
DS20	76.0	77.3	78.7	76.8	79.7	82.4	82.8	83.4	87.8	**88.9**	0.70	0.71	0.70	0.72	0.76	0.81	0.82	0.80	0.81	**0.84**
DS21	75.3	75.8	77.9	78.8	71.8	83.4	84	83.4	84.8	**87.2**	0.70	0.73	0.73	0.73	0.73	0.83	0.84	0.82	0.84	**0.85**
DS22	73.8	74.3	77.6	78.8	79.7	83.4	83	82.4	83.8	**84.9**	0.72	0.73	0.73	0.74	0.72	0.82	0.83	0.82	0.82	**0.85**
DS23	76.0	75.3	75.7	75.8	74.8	83.4	84	83.4	84.8	**85.2**	0.70	0.73	0.77	0.78	0.77	0.83	0.84	0.82	0.84	**0.85**
DS24	76.0	78.3	74.7	78.8	79.7	81.4	83	82.4	83.8	**84.8**	0.73	0.74	0.75	0.77	0.74	0.83	0.82	0.83	0.84	**0.85**
DS25	77.3	77.9	77.7	79.8	78.7	83.4	82	82.4	81	**86.3**	0.70	0.71	0.79	0.78	0.70	0.82	0.81	0.82	0.81	**0.83**
DS26	72.0	76.3	73.7	75.8	74.8	83.4	84	83.4	84.8	**85.2**	0.70	0.73	0.73	0.73	0.73	0.83	0.84	0.82	0.84	**0.85**
DS27	72.0	77.3	73.7	75.8	74.8	83.4	84	83.4	84.8	**87.2**	0.70	0.73	0.73	0.73	0.73	0.83	0.84	0.82	0.84	**0.85**
DS28	71.0	70.9	79.77	79.8	71.77	80.4	81	81.4	82	**82.9**	0.71	0.70	0.71	0.70	0.71	0.80	0.79	0.81	0.82	**0.83**
DS29	70.33	72.9	78.77	78.8	70.77	81.4	82	82.4	81	**83.9**	0.70	0.71	0.79	0.78	0.70	0.72	0.78	0.80	0.81	**0.82**
DS30	72.0	74.8	73.7	75.8	74.8	83.4	84	83.4	84.8	**86.2**	0.70	0.73	0.73	0.73	0.73	0.83	0.84	0.82	0.84	**0.85**

Significant values are in [bold].

**Table 23:** Accuracy and F-measure using FR2 with 100% labeled dataset.

Accuracy											F-measure									
ID	J48	RF	NB	K-star	AIRS	Y-J48	Y-RF	Y-NB	Y-Kstar	Y-AIRS	J48	RF	NB	K-star	AIRS	Y-J48	Y-RF	Y-NB	Y-Kstar	Y-AIRS
DS1	78.3	79.6	78.6	79.7	79.2	**88.4**	83.0	**88.4**	85.2	**88.4**	0.73	0.75	0.78	0.78	0.79	**0.83**	0.81	**0.83**	0.82	**0.83**
DS2	79.0	79.8	79.7	80.8	82.7	85.4	87.3	85.4	84.9	**87.8**	0.79	0.78	0.75	0.79	0.80	0.82	0.81	0.83	0.83	**0.85**
DS3	72.0	72.3	70.7	70.8	72.7	82.4	83	83.4	83	**84.9**	0.72	0.72	0.73	0.72	0.73	0.82	0.81	0.82	0.83	**0.84**
DS4	75.0	73.3	71.8	72.8	72.7	80.4	83	83.4	83.8	**83.8**	0.72	0.73	0.74	0.74	0.75	0.83	0.80	0.81	0.82	**0.83**
DS5	78.0	74.33	74.7	74.8	75.7	80.4	82	83.4	84.8	**84.9**	0.70	0.73	0.74	0.73	0.73	0.82	0.83	0.83	0.82	**0.84**
DS6	74.3	74.33	77.7	75.8	74.8	83.4	84	83.4	84.8	**85.2**	0.70	0.73	0.73	0.73	0.73	0.83	0.84	0.82	0.84	**0.85**
DS7	72.7	73.6	74.7	72.8	72.7	82.4	83	83.4	83.8	**83.8**	0.72	0.73	0.74	0.74	0.75	0.83	0.80	0.81	0.82	**0.85**
DS8	74.3	71.33	72.7	71.8	74.7	80.4	83	82.4	83	**83.7**	0.72	0.73	0.73	0.74	0.73	0.84	0.80	0.80	0.82	**0.84**
DS9	74.3	73.3	74.7	73.8	74.7	80.4	83	83.4	83.8	**85.8**	0.72	0.75	0.74	0.74	0.75	0.83	0.80	0.81	0.82	**0.83**
DS10	76.0	75.3	73.9	74.8	74.7	80.4	83	82.4	84.8	**87.8**	0.73	0.75	0.76	0.77	0.75	0.83	0.82	0.84	0.84	**0.85**
DS11	75.0	74.9	77.7	76.8	79.7	82.4	83.7	85.4	85.8	**86.8**	0.72	0.77	0.77	0.76	0.77	0.83	0.82	0.83	0.82	**0.84**
DS12	72.0	77.3	76.7	77.8	77.7	82.4	83	83.4	83	**83.9**	0.76	0.75	0.74	0.73	0.74	0.81	0.80	0.82	0.83	**0.84**
DS13	78.3	75.3	76.7	75.8	76.7	83.4	84	83.4	83	**84.8**	0.70	0.73	0.79	0.78	0.70	0.80	0.81	0.80	0.81	**0.82**
DS14	74.0	75.3	76.7	77.8	76.7	81.4	83	83.4	81.8	**83.8**	0.72	0.74	0.75	0.75	0.77	0.83	0.80	0.81	0.82	**0.84**
DS15	76.0	76.3	76.7	77.8	78.7	80.4	80	82.4	83.8	**83.9**	0.79	0.72	0.71	0.70	0.72	0.80	0.81	0.81	0.82	**0.83**
DS17	73.0	77.3	78.7	79.8	79.7	82.4	82.8	83.4	81.8	**83.9**	0.70	0.71	0.70	0.72	0.70	0.81	0.78	0.80	0.81	**0.84**
DS18	76.0	74.3	78.7	76.8	79.7	80.4	82.3	83.4	83.8	**84.1**	0.79	0.72	0.71	0.70	0.72	0.80	0.81	0.81	0.82	**0.83**
DS18	79.3	75.3	76.7	74.8	77.8	81.4	83	83.4	83	**83.8**	0.70	0.71	0.75	0.76	0.70	0.80	0.82	0.80	0.81	**0.82**
DS19	77.8	77.3	78.8	79.8	78.0	83.4	83	83.4	84	**85**	0.70	0.71	0.79	0.78	0.70	0.80	0.82	0.80	0.81	**0.82**
DS20	76.0	77.3	78.7	76.8	79.7	82.4	82.8	83.4	87.8	**88.9**	0.70	0.71	0.70	0.72	0.76	0.81	0.82	0.80	0.81	**0.84**
DS21	75.3	75.8	77.9	78.8	71.8	83.4	84	83.4	84.8	**87.2**	0.70	0.73	0.73	0.73	0.73	0.83	0.84	0.82	0.84	**0.85**
DS22	73.8	74.3	77.6	78.8	79.7	83.4	83	82.4	83.8	**84.9**	0.72	0.73	0.73	0.74	0.72	0.82	0.83	0.82	0.82	**0.85**
DS23	76.0	75.3	75.7	75.8	74.8	83.4	84	83.4	84.8	**85.2**	0.70	0.73	0.77	0.78	0.77	0.83	0.84	0.82	0.84	**0.85**
DS24	76.0	78.3	74.7	78.8	79.7	81.4	83	82.4	83.8	**84.8**	0.73	0.74	0.75	0.77	0.74	0.83	0.82	0.83	0.84	**0.85**
DS25	77.3	77.9	77.7	79.8	78.7	83.4	82	82.4	81	**86.3**	0.70	0.71	0.79	0.78	0.70	0.82	0.81	0.82	0.81	**0.83**
DS26	72.0	76.3	73.7	75.8	74.8	83.4	84	83.4	84.8	**85.2**	0.70	0.73	0.73	0.73	0.73	0.83	0.84	0.82	0.84	**0.85**
DS27	72.0	77.3	73.7	75.8	74.8	83.4	84	83.4	84.8	**87.2**	0.70	0.73	0.73	0.73	0.73	0.83	0.84	0.82	0.84	**0.85**
DS28	71.0	70.9	79.77	79.8	71.77	80.4	81	81.4	82	**82.9**	0.71	0.70	0.71	0.70	0.71	0.80	0.79	0.81	0.82	**0.83**
DS29	70.33	72.9	78.77	78.8	70.77	81.4	82	82.4	81	**83.9**	0.70	0.71	0.79	0.78	0.70	0.72	0.78	0.80	0.81	**0.82**
DS30	72.0	74.8	73.7	75.8	74.8	83.4	84	83.4	84.8	**86.2**	0.70	0.73	0.73	0.73	0.73	0.83	0.84	0.82	0.84	**0.85**

Significant values are in [bold].

**Table 24:** Accuracy and F-measure using FR3 with 5% labeled dataset.

Accuracy											F-measure									
ID	J48	RF	NB	K-star	AIRS	Y-J48	Y-RF	Y-NB	Y-Kstar	Y-AIRS	J48	RF	NB	K-star	AIRS	Y-J48	Y-RF	Y-NB	Y-Kstar	Y-AIRS
DS1	58.8	58.3	58.6	58.7	58.5	**63.4**	62	**63.4**	60.5	**63.4**	0.56	0.53	0.55	0.56	0.54	**0.62**	0.60	**0.62**	0.60	**0.62**
DS2	57.8	56.8	56.3	57.8	56.5	62.4	62.8	62.4	62.8	**62.9**	0.52	0.51	0.59	0.57	0.50	0.62	0.61	0.60	0.61	**0.63**
DS3	56.3	57.8	58.5	59.7	58.5	61.4	61.4	62.4	62	**64.9**	0.51	0.52	0.53	0.50	0.53	0.61	0.62	0.61	0.62	**0.64**
DS4	58.0	58.3	57.5	59.7	59.8	63.4	62.8	62.4	63.7	**65.3**	0.50	0.51	0.50	0.52	0.53	0.61	0.61	0.60	0.61	**0.64**
DS5	57.8	57.3	58.8	58.9	59.5	63.4	62.8	64.4	63.7	**65.8**	0.59	0.52	0.53	0.51	0.52	0.60	0.61	0.62	0.62	**0.64**
DS6	53.0	53.3	54.5	58.7	56.7	62.4	62.8	62.4	62.7	**65.8**	0.57	0.53	0.52	0.53	0.51	0.61	0.62	0.60	0.63	**0.65**
DS7	55.6	52.3	57.5	57.7	50.5	59.4	60.1	60.4	61.7	**63.9**	0.50	0.51	0.50	0.52	0.50	0.61	0.57	0.60	0.61	**0.62**
DS8	53.3	52.3	57.5	57.7	54.5	63.4	62	62.4	63	**65.3**	0.50	0.51	0.59	0.57	0.50	0.59	0.57	0.60	0.61	**0.62**
DS9	53.3	58.3	57.5	57.7	52.5	61.4	62	62.4	61	**64.1**	0.50	0.51	0.59	0.57	0.50	0.59	0.59	0.60	0.61	**0.63**
DS10	56.8	56.9	58.9	52.7	53.5	61.4	60.9	62.4	63.7	**63.9**	0.59	0.52	0.51	0.50	0.52	0.60	0.61	0.61	0.62	**0.63**
DS11	55.9	52.3	57.5	57.7	50.5	61.4	62	60.4	62.7	**63.4**	0.50	0.51	0.50	0.52	0.50	0.61	0.57	0.60	0.61	**0.62**
DS12	57.7	53.3	59.5	59.7	51.5	60.4	61	61.4	62	**61.9**	0.51	0.50	0.51	0.50	0.51	0.60	0.59	0.61	0.62	**0.63**
DS13	55.3	52.3	57.5	57.7	50.5	61.4	62	62.4	61	**65.4**	0.50	0.51	0.59	0.57	0.50	0.62	0.57	0.60	0.61	**0.63**
DS14	56.9	57.3	59.5	59.7	54.5	62.4	61	61.4	62	**65.9**	0.51	0.50	0.51	0.50	0.51	0.60	0.59	0.61	0.62	**0.63**
DS15	53.9	53.3	50.5	52.7	53.5	59.4	61	62.4	63.7	**64.9**	0.59	0.52	0.51	0.50	0.52	0.60	0.61	0.61	0.62	**0.63**
DS16	54.3	53.3	57.5	57.7	53.5	61.4	62	60.4	61.7	**63.9**	0.50	0.51	0.50	0.52	0.50	0.61	0.57	0.60	0.61	**0.64**
DS17	53.0	51.3	54.5	52.7	53.5	59.4	60	62.4	63.7	**63.9**	0.59	0.52	0.51	0.50	0.52	0.60	0.61	0.61	0.62	**0.63**
DS18	53.4	54.3	57.5	57.7	50.5	61.4	62	62.4	61	**65.2**	0.50	0.51	0.59	0.57	0.50	0.59	0.60	0.60	0.61	**0.62**
DS19	53.3	52.3	57.5	57.7	50.5	62.4	62	62.4	61	**66.7**	0.50	0.51	0.59	0.57	0.50	0.58	0.59	0.60	0.61	**0.62**
DS20	56.0	52.3	57.5	59.7	53.5	59.4	62	60.4	61.7	**65.8**	0.50	0.51	0.50	0.52	0.50	0.61	0.57	0.60	0.61	**0.64**
DS21	53.3	52.3	57.5	57.7	51.6	61.4	62	62.4	61	**64.9**	0.50	0.51	0.59	0.57	0.50	0.62	0.57	0.60	0.61	**0.62**
DS22	51.0	53.4	57.5	57.7	50.5	59.4	62	60.4	61.7	**64.8**	0.50	0.51	0.50	0.52	0.50	0.61	0.57	0.60	0.61	**0.64**
DS23	55.7	51.3	50.5	52.7	53.5	59.4	60	62.4	63.7	**64.9**	0.59	0.52	0.51	0.50	0.52	0.60	0.61	0.61	0.62	**0.63**
DS24	54.6	54.3	57.5	57.7	50.5	59.4	62	60.4	61.7	**64.8**	0.50	0.51	0.50	0.52	0.50	0.61	0.57	0.60	0.61	**0.64**
DS25	54.3	53.4	57.5	57.7	50.5	61.4	62	62.4	61	**63.8**	0.50	0.51	0.59	0.57	0.50	0.52	0.57	0.60	0.61	**0.62**
DS26	54.0	54.3	57.5	57.7	50.5	59.4	62	60.4	61.7	**64.7**	0.50	0.51	0.50	0.52	0.50	0.61	0.57	0.60	0.61	**0.64**
DS27	57.3	52.3	57.5	57.7	50.5	61.4	62	62.4	61	**62.8**	0.50	0.51	0.59	0.57	0.50	0.59	0.59	0.60	0.61	**0.62**
DS28	53.8	54.3	59.5	59.7	51.5	60.4	61	61.4	62	**62.9**	0.51	0.50	0.51	0.50	0.51	0.60	0.59	0.61	0.62	**0.63**
DS29	54.3	52.3	57.5	57.7	50.5	61.4	62	62.4	61	**62.9**	0.50	0.51	0.59	0.57	0.50	0.59	0.57	0.60	0.61	**0.62**
DS30	54.0	52.3	52.5	53.7	52.7	61.4	62	61.4	62.7	**63.2**	0.57	0.50	0.52	0.51	0.51	0.61	0.62	0.60	0.63	**0.64**

Significant values are in [bold].

**Table 25:** Accuracy and F-measure using FR3 with 10% labeled dataset.

Accuracy											F-measure									
ID	J48	RF	NB	K-star	AIRS	Y-J48	Y-RF	Y-NB	Y-Kstar	Y-AIRS	J48	RF	NB	K-star	AIRS	Y-J48	Y-RF	Y-NB	Y-Kstar	Y-AIRS
DS1	56.9	58.8	58.5	59.5	57.5	**66.8**	65.2	**66.8**	65.5	**66.8**	0.52	0.52	0.53	0.53	0.54	**0.63**	0.62	**0.63**	0.62	**0.63**
DS2	53.8	53.4	54.5	55.6	56.7	63.4	63.5	63.4	64.9	**65.2**	0.52	0.53	0.50	0.51	0.52	0.55	0.59	0.62	0.63	**0.64**
DS3	52.8	52.3	52.5	52.6	52.5	62.4	63.2	63.4	63.3	**63.9**	0.52	0.52	0.53	0.52	0.53	0.62	0.63	0.62	0.63	**0.65**
DS4	52.7	53.8	58.5	58.6	53.5	62.4	63	63.4	63.6	**64.8**	0.52	0.53	0.53	0.54	0.58	0.63	0.62	0.61	0.62	**0.64**
DS5	53.0	54.3	54.5	55.6	55.5	63.4	62	63.4	64.6	**66.2**	0.50	0.53	0.54	0.53	0.53	0.62	0.61	0.61	0.62	**0.65**
DS6	52.8	56.3	53.5	57.6	58.6	63.4	64.5	65.4	64.6	**65.8**	0.52	0.53	0.53	0.53	0.54	0.63	0.64	0.63	0.64	**0.66**
DS7	56.0	56.6	56.5	57.6	57.5	61.4	63.3	63.4	63.6	**65.6**	0.52	0.53	0.54	0.56	0.55	0.63	0.62	0.61	0.62	**0.64**
DS8	56.3	52.3	52.5	55.6	57.5	63.4	62	64.4	63.3	**64.5**	0.50	0.52	0.52	0.51	0.52	0.53	0.55	0.61	0.62	**0.63**
DS9	57.6	54.3	55.5	56.6	56.5	62.4	63.3	63.4	64.6	**65.6**	0.52	0.53	0.54	0.54	0.55	0.63	0.62	0.62	0.62	**0.64**
DS10	52.0	53.3	51.5	52.6	52.5	60.4	63	62.4	62.6	**63.6**	0.52	0.53	0.54	0.54	0.55	0.63	0.60	0.61	0.62	**0.63**
DS11	52.8	52.3	51.5	52.6	52.5	62.4	63.1	62.4	62.4	**62.6**	0.52	0.53	0.54	0.54	0.55	0.63	0.60	0.61	0.62	**0.63**
DS12	51.0	50.3	51.5	50.6	51.5	61.4	61.1	61.4	62	**62.9**	0.51	0.50	0.51	0.50	0.51	0.60	0.59	0.61	0.62	**0.63**
DS13	52.3	54.3	51.5	52.6	53.5	63.4	64.1	63.4	63.2	**64.6**	0.50	0.51	0.59	0.56	0.50	0.52	0.56	0.60	0.61	**0.62**
DS14	52.0	53.3	51.5	52.6	52.5	60.4	63	63.4	63.6	**64.0**	0.52	0.53	0.54	0.54	0.55	0.63	0.60	0.61	0.62	**0.63**
DS15	54.0	51.3	50.5	52.6	53.5	59.4	60	62.4	63.6	**64.9**	0.59	0.52	0.51	0.50	0.52	0.60	0.61	0.61	0.62	**0.63**
DS15	53.0	52.3	52.5	51.6	50.5	59.4	62	60.4	61.6	**63.0**	0.50	0.51	0.50	0.52	0.50	0.61	0.56	0.60	0.61	**0.64**
DS16	52.5	53.3	53.5	52.6	53.5	59.4	62.1	62.4	61.6	**63.9**	0.59	0.52	0.51	0.50	0.52	0.60	0.61	0.61	0.62	**0.63**
DS18	53.3	56.3	56.5	56.6	50.5	61.4	63	63.4	63.4	**63.6**	0.50	0.51	0.59	0.56	0.50	0.52	0.56	0.60	0.61	**0.62**
DS19	50.3	53.3	51.5	52.6	54.5	63.4	63.5	63.4	64.3	**65.1**	0.50	0.51	0.59	0.56	0.50	0.52	0.56	0.60	0.61	**0.62**
DS20	53.8	52.3	51.5	56.6	53.5	62.4	62.6	63.4	63.6	**63.9**	0.50	0.51	0.50	0.52	0.50	0.61	0.56	0.60	0.61	**0.64**
DS21	55.4	54.3	53.5	55.6	54.6	63.4	64	63.4	64.6	**65.2**	0.50	0.53	0.53	0.53	0.53	0.63	0.64	0.62	0.64	**0.65**
DS22	54.7	54.3	53.5	56.6	50.5	62.4	63.3	62.4	63.6	**64.0**	0.52	0.53	0.53	0.54	0.52	0.62	0.63	0.62	0.62	**0.65**
DS23	55.6	54.3	53.5	55.6	54.6	63.4	64.1	63.4	64.6	**65.0**	0.50	0.53	0.53	0.53	0.53	0.63	0.64	0.62	0.64	**0.65**
DS24	56.0	56.3	53.5	56.6	50.5	62.4	63.4	62.4	63.6	**65.6**	0.53	0.54	0.55	0.55	0.54	0.63	0.62	0.63	0.64	**0.65**
DS25	56.3	57.3	53.5	50.6	50.5	61.4	62	62.4	61	**63.2**	0.50	0.51	0.59	0.56	0.50	0.52	0.56	0.60	0.61	**0.62**
DS26	52.8	53.6	54.5	55.6	54.6	63.4	64.1	63.4	63.6	**65.2**	0.50	0.53	0.53	0.53	0.53	0.63	0.64	0.62	0.64	**0.65**
DS27	53.5	54.3	53.5	55.6	54.6	63.4	64	63.4	64.6	**65.2**	0.50	0.53	0.53	0.53	0.53	0.63	0.64	0.62	0.64	**0.65**
DS28	53.0	50.3	59.5	59.6	51.5	60.4	61	61.4	62	**64.9**	0.51	0.50	0.51	0.50	0.51	0.60	0.59	0.61	0.62	**0.63**
DS29	54.3	52.3	56.5	56.6	50.5	61.4	62	62.4	61	**64.9**	0.50	0.51	0.59	0.56	0.50	0.52	0.56	0.60	0.61	**0.62**
DS30	55.3	54.3	53.5	55.6	54.6	64.4	64	63.4	64.6	**65.6**	0.50	0.53	0.53	0.53	0.53	0.63	0.64	0.62	0.64	**0.65**

Significant values are in [bold].

**Table 26:** Accuracy and F-measure using FR3 with optimal number of labeled dataset (i.e., 60%).

Accuracy											F-measure									
ID	J48	RF	NB	K-star	AIRS	Y-J48	Y-RF	Y-NB	Y-Kstar	Y-AIRS	J48	RF	NB	K-star	AIRS	Y-J48	Y-RF	Y-NB	Y-Kstar	Y-AIRS
DS1	76.3	78.6	79.6	78.7	79.2	**86.4**	83.0	**86.4**	85.2	**86.4**	0.72	0.74	0.78	0.78	0.79	**0.82**	0.80	**0.82**	0.81	**0.82**
DS2	79.2	79.3	75.7	80.8	82.7	84.4	87.8	86.4	85.9	**88.8**	0.79	0.78	0.75	0.78	0.81	0.82	0.83	0.83	0.83	**0.87**
DS3	72.6	72.7	72.7	72.8	75.7	82.4	83.8	86.4	85.7	**88.9**	0.72	0.72	0.73	0.72	0.70	0.82	0.81	0.82	0.83	**0.86**
DS4	75.3	76.3	73.8	73.8	72.7	81.4	83.3	83.4	82.8	**85.8**	0.72	0.73	0.74	0.74	0.75	0.83	0.80	0.81	0.81	**0.85**
DS5	78.6	74.3	74.8	75.8	76.7	81.4	82.6	83.4	84.8	**86.9**	0.70	0.73	0.74	0.73	0.73	0.83	0.82	0.82	0.83	**0.85**
DS6	74.3	75.7	78.7	75.8	77.8	83.4	84.1	83.4	84.8	**87.2**	0.70	0.73	0.73	0.73	0.73	0.82	0.83	0.82	0.84	**0.86**
DS7	72.5	75.6	74.7	72.8	72.7	81.4	83	82.4	82.8	**84.8**	0.72	0.73	0.74	0.74	0.75	0.82	0.81	0.82	0.82	**0.84**
DS8	74.3	73.3	72.7	71.8	74.7	82.4	83.3	82.4	83	**86.7**	0.72	0.73	0.73	0.74	0.73	0.84	0.80	0.80	0.82	**0.85**
DS9	72.3	78.3	74.6	72.8	74.7	81.4	83.3	83.4	83.8	**87.8**	0.72	0.75	0.74	0.74	0.75	0.83	0.82	0.82	0.82	**0.84**
DS10	76.0	75.3	73.9	74.8	74.6	80.4	83	82.4	84.8	**86.8**	0.73	0.75	0.76	0.77	0.75	0.82	0.82	0.84	0.84	**0.86**
DS11	75.0	73.9	76.7	76.8	78.7	82.4	83.7	83.4	85.8	**87.8**	0.72	0.77	0.77	0.76	0.77	0.83	0.82	0.83	0.82	**0.85**
DS12	72.0	76.3	76.7	74.8	77.7	82.4	83.2	83.4	83	**85.9**	0.76	0.75	0.74	0.73	0.73	0.81	0.80	0.82	0.83	**0.86**
DS13	78.3	75.3	76.7	75.8	76.7	82.4	84.6	84.4	83.5	**87.8**	0.70	0.73	0.79	0.78	0.70	0.80	0.81	0.80	0.81	**0.86**
DS14	73.9	77.3	76.7	77.8	76.7	82.4	83	83.4	81.8	**87.8**	0.72	0.74	0.75	0.75	0.78	0.83	0.82	0.81	0.82	**0.86**
DS15	76.0	76.3	76.7	77.8	78.7	80.4	82	83.4	83.8	**86.9**	0.79	0.72	0.71	0.70	0.72	0.80	0.81	0.81	0.82	**0.83**
DS17	73.0	77.3	78.7	79.8	79.7	82.4	82.8	83.4	81.8	**83.9**	0.70	0.71	0.70	0.72	0.70	0.81	0.78	0.80	0.81	**0.84**
DS18	76.0	74.3	78.7	76.8	79.7	80.4	82.3	83.4	83.8	**84.1**	0.79	0.72	0.71	0.70	0.72	0.80	0.81	0.81	0.82	**0.83**
DS18	79.3	75.3	76.7	74.8	77.8	81.4	83	83.4	83	**83.8**	0.70	0.71	0.75	0.76	0.70	0.80	0.82	0.80	0.81	**0.82**
DS19	77.8	77.3	78.8	79.8	78.0	83.4	83	85.4	84	**87.6**	0.70	0.71	0.79	0.78	0.70	0.80	0.82	0.80	0.81	**0.82**
DS20	76.0	77.3	78.7	76.8	79.7	82.4	82.8	83.4	87.8	**86.9**	0.70	0.71	0.70	0.72	0.76	0.81	0.82	0.80	0.81	**0.84**
DS21	75.3	75.8	77.9	78.8	70.8	82.4	84	83.4	84.8	**88.2**	0.70	0.73	0.73	0.73	0.73	0.83	0.84	0.82	0.84	**0.85**
DS22	73.8	74.3	77.6	78.8	79.7	83.4	83	82.4	83.8	**84.9**	0.72	0.73	0.73	0.74	0.72	0.82	0.83	0.82	0.82	**0.85**
DS23	76.0	75.3	74.7	75.8	74.8	82.4	84	83.4	84.8	**85.2**	0.70	0.73	0.77	0.78	0.77	0.83	0.84	0.82	0.84	**0.85**
DS24	76.0	78.3	71.7	78.8	79.7	81.4	83	82.4	83.8	**87.8**	0.73	0.74	0.75	0.77	0.74	0.83	0.82	0.83	0.84	**0.85**
DS25	77.3	77.9	77.7	79.8	78.7	83.4	82	82.4	81	**86.5**	0.70	0.71	0.79	0.78	0.70	0.82	0.81	0.82	0.81	**0.83**
DS26	75.0	76.3	73.7	75.8	77.8	84.4	84	83.4	84.8	**88.2**	0.70	0.73	0.73	0.73	0.73	0.83	0.84	0.82	0.84	**0.85**
DS27	77.0	77.3	73.7	75.8	74.8	83.4	84	86.4	84.8	**89.2**	0.70	0.73	0.73	0.73	0.73	0.83	0.84	0.82	0.84	**0.85**
DS28	75.0	70.9	79.77	79.8	71.77	80.4	83	81.4	82	**88.9**	0.71	0.70	0.71	0.70	0.71	0.80	0.79	0.81	0.82	**0.83**
DS29	77.3	72.9	78.77	78.8	70.77	84.4	82	82.4	81	**83.9**	0.70	0.71	0.79	0.78	0.70	0.72	0.78	0.80	0.81	**0.82**
DS30	76.0	77.8	79.7	75.8	74.8	83.4	84	83.4	84.8	**89.2**	0.70	0.73	0.73	0.73	0.73	0.83	0.84	0.82	0.84	**0.85**

Significant values are in [bold].

**Table 27:** Accuracy and F-measure using FR3 with 100% labeled dataset.

Accuracy											F-measure									
ID	J48	RF	NB	K-star	AIRS	Y-J48	Y-RF	Y-NB	Y-Kstar	Y-AIRS	J48	RF	NB	K-star	AIRS	Y-J48	Y-RF	Y-NB	Y-Kstar	Y-AIRS
DS1	76.3	78.6	79.6	78.7	79.2	**86.4**	83.0	**86.4**	85.2	**86.4**	0.72	0.74	0.78	0.78	0.79	**0.82**	0.80	**0.82**	0.81	**0.82**
DS2	79.2	79.3	75.7	80.8	82.7	84.4	87.8	86.4	85.9	**88.8**	0.79	0.78	0.75	0.78	0.81	0.82	0.83	0.83	0.83	**0.87**
DS3	72.6	72.7	72.7	72.8	75.7	82.4	83.8	86.4	85.7	**88.9**	0.72	0.72	0.73	0.72	0.70	0.82	0.81	0.82	0.83	**0.86**
DS4	75.3	76.3	73.8	73.8	72.7	81.4	83.3	83.4	82.8	**85.8**	0.72	0.73	0.74	0.74	0.75	0.83	0.80	0.81	0.81	**0.85**
DS5	78.6	74.3	74.8	75.8	76.7	81.4	82.6	83.4	84.8	**86.9**	0.70	0.73	0.74	0.73	0.73	0.83	0.82	0.82	0.83	**0.85**
DS6	74.3	75.7	78.7	75.8	77.8	83.4	84.1	83.4	84.8	**87.2**	0.70	0.73	0.73	0.73	0.73	0.82	0.83	0.82	0.84	**0.86**
DS7	72.5	75.6	74.7	72.8	72.7	81.4	83	82.4	82.8	**84.8**	0.72	0.73	0.74	0.74	0.75	0.82	0.81	0.82	0.82	**0.84**
DS8	74.3	73.3	72.7	71.8	74.7	82.4	83.3	82.4	83	**86.7**	0.72	0.73	0.73	0.74	0.73	0.84	0.80	0.80	0.82	**0.85**
DS9	72.3	78.3	74.6	72.8	74.7	81.4	83.3	83.4	83.8	**87.8**	0.72	0.75	0.74	0.74	0.75	0.83	0.82	0.82	0.82	**0.84**
DS10	76.0	75.3	73.9	74.8	74.6	80.4	83	82.4	84.8	**86.8**	0.73	0.75	0.76	0.77	0.75	0.82	0.82	0.84	0.84	**0.86**
DS11	75.0	73.9	76.7	76.8	78.7	82.4	83.7	83.4	85.8	**87.8**	0.72	0.77	0.77	0.76	0.77	0.83	0.82	0.83	0.82	**0.85**
DS12	72.0	76.3	76.7	74.8	77.7	82.4	83.2	83.4	83	**85.9**	0.76	0.75	0.74	0.73	0.73	0.81	0.80	0.82	0.83	**0.86**
DS13	78.3	75.3	76.7	75.8	76.7	82.4	84.6	84.4	83.5	**87.8**	0.70	0.73	0.79	0.78	0.70	0.80	0.81	0.80	0.81	**0.86**
DS14	73.9	77.3	76.7	77.8	76.7	82.4	83	83.4	81.8	**87.8**	0.72	0.74	0.75	0.75	0.78	0.83	0.82	0.81	0.82	**0.86**
DS15	76.0	76.3	76.7	77.8	78.7	80.4	82	83.4	83.8	**86.9**	0.79	0.72	0.71	0.70	0.72	0.80	0.81	0.81	0.82	**0.83**
DS17	73.0	77.3	78.7	79.8	79.7	82.4	82.8	83.4	81.8	**83.9**	0.70	0.71	0.70	0.72	0.70	0.81	0.78	0.80	0.81	**0.84**
DS18	76.0	74.3	78.7	76.8	79.7	80.4	82.3	83.4	83.8	**84.1**	0.79	0.72	0.71	0.70	0.72	0.80	0.81	0.81	0.82	**0.83**
DS18	79.3	75.3	76.7	74.8	77.8	81.4	83	83.4	83	**83.8**	0.70	0.71	0.75	0.76	0.70	0.80	0.82	0.80	0.81	**0.82**
DS19	77.8	77.3	78.8	79.8	78.0	83.4	83	85.4	84	**87.6**	0.70	0.71	0.79	0.78	0.70	0.80	0.82	0.80	0.81	**0.82**
DS20	76.0	77.3	78.7	76.8	79.7	82.4	82.8	83.4	87.8	**86.9**	0.70	0.71	0.70	0.72	0.76	0.81	0.82	0.80	0.81	**0.84**
DS21	75.3	75.8	77.9	78.8	70.8	82.4	84	83.4	84.8	**88.2**	0.70	0.73	0.73	0.73	0.73	0.83	0.84	0.82	0.84	**0.85**
DS22	73.8	74.3	77.6	78.8	79.7	83.4	83	82.4	83.8	**84.9**	0.72	0.73	0.73	0.74	0.72	0.82	0.83	0.82	0.82	**0.85**
DS23	76.0	75.3	74.7	75.8	74.8	82.4	84	83.4	84.8	**85.2**	0.70	0.73	0.77	0.78	0.77	0.83	0.84	0.82	0.84	**0.85**
DS24	76.0	78.3	71.7	78.8	79.7	81.4	83	82.4	83.8	**87.8**	0.73	0.74	0.75	0.77	0.74	0.83	0.82	0.83	0.84	**0.85**
DS25	77.3	77.9	77.7	79.8	78.7	83.4	82	82.4	81	**86.5**	0.70	0.71	0.79	0.78	0.70	0.82	0.81	0.82	0.81	**0.83**
DS26	75.0	76.3	73.7	75.8	77.8	84.4	84	83.4	84.8	**88.2**	0.70	0.73	0.73	0.73	0.73	0.83	0.84	0.82	0.84	**0.85**
DS27	77.0	77.3	73.7	75.8	74.8	83.4	84	86.4	84.8	**89.2**	0.70	0.73	0.73	0.73	0.73	0.83	0.84	0.82	0.84	**0.85**
DS28	75.0	70.9	79.77	79.8	71.77	80.4	83	81.4	82	**88.9**	0.71	0.70	0.71	0.70	0.71	0.80	0.79	0.81	0.82	**0.83**
DS29	77.3	72.9	78.77	78.8	70.77	84.4	82	82.4	81	**83.9**	0.70	0.71	0.79	0.78	0.70	0.72	0.78	0.80	0.81	**0.82**
DS30	76.0	77.8	79.7	75.8	74.8	83.4	84	83.4	84.8	**89.2**	0.70	0.73	0.73	0.73	0.73	0.83	0.84	0.82	0.84	**0.85**

Significant values are in [bold].

**Table 28:** Accuracy and F-measure using FR4 with 5% labeled dataset.

Accuracy											F-measure									
ID	J48	RF	NB	K-star	AIRS	Y-J48	Y-RF	Y-NB	Y-Kstar	Y-AIRS	J48	RF	NB	K-star	AIRS	Y-J48	Y-RF	Y-NB	Y-Kstar	Y-AIRS
DS1	57.3	56.3	59.5	57.7	57.5	**61.4**	60.5	**61.4**	60.5	**61.4**	0.56	0.50	0.55	0.56	0.54	**0.61**	0.60	**0.61**	0.60	**0.61**
DS2	53.8	52.3	56.5	57.7	56.5	62.4	62.8	62.4	61.8	**63.8**	0.52	0.51	0.59	0.57	0.50	0.62	0.61	0.60	0.61	**0.63**
DS3	56.0	57.3	59.5	59.7	57.5	61.4	61	62.4	62	**62.9**	0.51	0.50	0.51	0.50	0.51	0.61	0.59	0.61	0.62	**0.63**
DS4	58.0	58.3	57.5	59.7	59.5	64.4	62	60.4	61.7	**65.7**	0.50	0.51	0.50	0.52	0.50	0.61	0.59	0.60	0.61	**0.64**
DS5	57.8	57.3	58.5	58.7	59.5	61.4	62.8	62.4	63.7	**63.8**	0.59	0.52	0.51	0.50	0.52	0.60	0.61	0.61	0.62	**0.63**
DS6	53.5	53.6	52.5	57.7	52.7	62.4	62	61.4	62.7	**63.2**	0.57	0.50	0.52	0.51	0.51	0.61	0.62	0.60	0.63	**0.64**
DS7	50.0	53.3	57.5	57.7	50.5	59.4	61.1	60.4	61.7	**63.9**	0.50	0.51	0.50	0.52	0.50	0.61	0.57	0.60	0.61	**0.62**
DS8	52.3	52.5	57.5	57.7	54.5	62.4	62	62.4	61	**63**	0.50	0.51	0.59	0.57	0.50	0.59	0.57	0.60	0.61	**0.62**
DS9	51.3	52.6	57.5	57.7	52.5	62.4	62.1	62.4	61	**63.7**	0.50	0.51	0.59	0.57	0.50	0.59	0.59	0.60	0.61	**0.63**
DS10	56.0	56.3	58.5	52.7	53.5	61.4	60.9	62.4	63.7	**63.9**	0.59	0.52	0.51	0.50	0.52	0.60	0.61	0.61	0.62	**0.63**
DS11	53.0	52.3	57.5	57.7	50.5	62.4	62	60.4	61.7	**63.0**	0.50	0.51	0.50	0.52	0.50	0.61	0.57	0.60	0.61	**0.62**
DS12	53.0	53.3	57.5	57.7	51.5	60.4	61	61.4	62	**62.9**	0.51	0.50	0.51	0.50	0.51	0.60	0.59	0.61	0.62	**0.63**
DS13	56.3	52.3	57.5	56.7	50.5	61.4	62	62.4	61	**63**	0.50	0.51	0.59	0.57	0.50	0.62	0.57	0.60	0.61	**0.63**
DS14	53.8	53.3	59.5	59.7	51.5	61.4	61	61.4	62	**62.8**	0.51	0.50	0.51	0.50	0.51	0.60	0.59	0.61	0.62	**0.62**
DS15	53.7	53.3	50.5	52.7	53.5	59.4	60	62.4	63.7	**63.9**	0.59	0.52	0.51	0.50	0.52	0.60	0.61	0.61	0.62	**0.64**
DS16	54.7	56.3	57.5	57.7	50.5	61.4	62	60.4	61.7	**63.4**	0.50	0.51	0.50	0.52	0.50	0.61	0.57	0.60	0.61	**0.64**
DS17	50.8	51.3	50.5	52.7	53.5	59.4	60	62.4	63.7	**63.9**	0.59	0.52	0.51	0.50	0.52	0.60	0.61	0.61	0.62	**0.63**
DS18	52.4	54.3	57.5	57.7	50.5	61.4	62	62.4	61	**62.8**	0.50	0.51	0.59	0.57	0.50	0.59	0.60	0.60	0.61	**0.62**
DS19	53.3	56.3	57.5	57.7	50.5	61.4	62	62.4	61	**62.7**	0.50	0.51	0.59	0.57	0.50	0.58	0.59	0.60	0.61	**0.62**
DS20	55.0	52.3	57.5	57.7	53.5	59.4	62	60.4	61.7	**63.0**	0.50	0.51	0.50	0.52	0.50	0.61	0.57	0.60	0.61	**0.64**
DS21	52.3	53.3	54.5	57.7	51.6	61.4	62	62.4	61	**63.6**	0.50	0.51	0.59	0.57	0.50	0.62	0.57	0.60	0.61	**0.62**
DS22	52.0	53.4	57.5	57.7	50.5	59.4	62	60.4	61.7	**63.7**	0.50	0.51	0.50	0.52	0.50	0.61	0.57	0.60	0.61	**0.64**
DS23	53.4	53.3	52.5	51.7	53.5	59.4	60	62.4	63.7	**63.9**	0.59	0.52	0.51	0.50	0.52	0.60	0.61	0.61	0.62	**0.63**
DS24	53.0	54.3	57.5	57.7	50.5	59.4	62	60.4	61.7	**63.7**	0.50	0.51	0.50	0.52	0.50	0.61	0.57	0.60	0.61	**0.64**
DS25	54.3	53.4	57.5	56.7	50.5	61.4	62	62.4	61	**64.2**	0.50	0.51	0.59	0.57	0.50	0.52	0.57	0.60	0.61	**0.62**
DS26	54.0	54.3	57.5	52.7	50.5	59.4	62	60.4	61.7	**63.0**	0.50	0.51	0.50	0.52	0.50	0.61	0.57	0.60	0.61	**0.64**
DS27	53.3	52.3	57.5	53.7	50.5	61.4	62	62.4	61	**63.1**	0.50	0.51	0.59	0.57	0.50	0.59	0.59	0.60	0.61	**0.62**
DS28	53.0	54.3	59.5	59.7	51.5	60.4	61	61.4	62	**62.9**	0.51	0.50	0.51	0.50	0.51	0.60	0.59	0.61	0.62	**0.63**
DS29	54.3	52.3	57.5	57.7	50.5	61.4	62	62.4	61	**63.2**	0.50	0.51	0.59	0.57	0.50	0.59	0.57	0.60	0.61	**0.62**
DS30	54.0	52.3	52.5	53.7	52.7	61.4	62	61.4	62.7	**63.2**	0.57	0.50	0.52	0.51	0.51	0.61	0.62	0.60	0.63	**0.64**

Significant values are in [bold].

**Table 29:** Accuracy and F-measure using FR4 with 10% labeled dataset.

Accuracy											F-measure									
ID	J48	RF	NB	K-star	AIRS	Y-J48	Y-RF	Y-NB	Y-Kstar	Y-AIRS	J48	RF	NB	K-star	AIRS	Y-J48	Y-RF	Y-NB	Y-Kstar	Y-AIRS
DS1	53.9	58.6	58.7	58.5	57.5	**65.8**	65.2	**65.8**	65.5	**65.8**	0.53	0.52	0.53	0.55	0.54	**0.62**	0.61	**0.62**	0.61	**0.62**
DS2	53.9	54.1	54.8	55.6	57.7	63.4	62.5	63.4	64.9	**66.2**	0.52	0.53	0.50	0.51	0.52	0.55	0.59	0.62	0.63	**0.65**
DS3	52.8	52.3	52.5	52.6	58.5	63.4	63.7	63.9	63.6	**64.1**	0.52	0.52	0.53	0.52	0.53	0.62	0.63	0.62	0.63	**0.65**
DS4	51.9	53.8	58.7	58.6	53.5	62.4	63.1	62.4	62.6	**63.8**	0.57	0.53	0.53	0.54	0.58	0.63	0.63	0.63	0.62	**0.64**
DS5	53.0	54.3	54.5	55.6	55.5	63.4	62	63.4	64.6	**66.2**	0.50	0.53	0.54	0.53	0.53	0.62	0.61	0.61	0.62	**0.65**
DS6	52.8	56.3	53.5	57.6	58.6	63.4	64.5	65.4	64.6	**65.8**	0.52	0.53	0.53	0.53	0.54	0.63	0.64	0.63	0.64	**0.66**
DS7	56.0	56.6	56.5	57.6	57.5	61.4	63.3	63.4	63.6	**65.6**	0.52	0.53	0.54	0.56	0.55	0.63	0.62	0.61	0.62	**0.64**
DS8	56.3	52.3	52.5	55.6	57.5	63.4	62	64.4	63.3	**64.5**	0.50	0.52	0.52	0.51	0.52	0.53	0.55	0.61	0.62	**0.63**
DS9	57.6	54.3	55.5	56.6	56.5	62.4	63.3	63.4	64.6	**65.6**	0.52	0.53	0.54	0.54	0.55	0.63	0.62	0.62	0.62	**0.64**
DS10	52.0	53.3	51.5	52.6	52.5	60.4	63	62.4	62.6	**63.6**	0.52	0.53	0.54	0.54	0.55	0.63	0.60	0.61	0.62	**0.63**
DS11	56.8	52.3	53.5	52.6	52.5	61.4	63.8	62.7	62.4	**65.9**	0.52	0.53	0.54	0.54	0.55	0.63	0.60	0.61	0.62	**0.63**
DS12	51.0	50.3	51.5	50.6	51.5	63.4	61.8	63.4	62	**64.9**	0.51	0.50	0.51	0.50	0.51	0.60	0.59	0.61	0.62	**0.63**
DS13	52.3	54.3	51.5	52.6	53.5	63.8	64.9	63.4	63.7	**65.6**	0.50	0.51	0.59	0.56	0.50	0.52	0.56	0.60	0.61	**0.62**
DS14	52.0	53.3	51.5	52.6	52.5	62.4	63.3	63.4	63.6	**64.7**	0.52	0.53	0.54	0.54	0.55	0.63	0.60	0.61	0.62	**0.63**
DS15	58.3	58.3	57.5	58.6	51.5	59.4	62.3	63.7	63.6	**64.9**	0.59	0.52	0.51	0.50	0.52	0.60	0.61	0.61	0.62	**0.63**
DS15	53.9	53.8	52.8	57.6	53.5	59.4	62	60.4	63.6	**63.9**	0.50	0.51	0.50	0.52	0.50	0.61	0.56	0.60	0.61	**0.64**
DS16	52.5	53.3	53.5	52.6	53.5	59.4	62.9	62.4	61.6	**64.1**	0.59	0.52	0.51	0.50	0.52	0.60	0.61	0.61	0.62	**0.63**
DS18	53.3	56.3	56.5	56.8	56.5	61.4	63	63.4	63.4	**63.9**	0.50	0.51	0.59	0.56	0.50	0.52	0.56	0.60	0.61	**0.62**
DS19	58.3	58.3	51.5	52.6	59.5	63.4	63.5	63.4	64.3	**65.3**	0.50	0.51	0.59	0.56	0.50	0.52	0.56	0.60	0.61	**0.62**
DS20	53.8	52.3	51.5	56.6	53.5	62.4	62.6	63.8	63.6	**64.9**	0.50	0.51	0.50	0.52	0.50	0.61	0.56	0.60	0.61	**0.64**
DS21	58.4	54.3	59.5	55.6	54.6	63.4	64.1	63.4	64.6	**65.3**	0.50	0.53	0.53	0.53	0.53	0.63	0.64	0.62	0.64	**0.65**
DS22	59.7	57.3	58.5	57.6	53.5	62.4	63.3	62.4	63.6	**64.8**	0.52	0.53	0.53	0.54	0.52	0.62	0.63	0.62	0.62	**0.65**
DS23	55.6	54.3	53.5	55.6	54.6	63.4	64.1	63.4	64.6	**65.0**	0.50	0.53	0.53	0.53	0.53	0.63	0.64	0.62	0.64	**0.65**
DS24	56.0	56.3	53.5	56.6	50.5	62.4	63.4	62.4	63.6	**64.6**	0.53	0.54	0.55	0.55	0.54	0.63	0.62	0.63	0.64	**0.65**
DS25	57.3	57.3	53.5	50.6	50.5	62.4	62.2	63.4	63	**65.2**	0.50	0.51	0.59	0.56	0.50	0.52	0.56	0.60	0.61	**0.62**
DS26	59.8	53.6	54.5	55.6	54.6	63.4	64.1	63.4	63.6	**64.9**	0.50	0.53	0.53	0.53	0.53	0.63	0.64	0.62	0.64	**0.65**
DS27	57.5	54.3	53.5	55.6	54.6	63.4	64.2	63.4	64.8	**65.2**	0.50	0.53	0.53	0.53	0.53	0.63	0.64	0.62	0.64	**0.65**
DS28	53.0	50.3	59.5	59.6	51.5	60.4	61	61.4	62	**64.9**	0.51	0.50	0.51	0.50	0.51	0.60	0.59	0.61	0.62	**0.63**
DS29	58.3	56.3	56.9	59.6	50.5	63.4	62.9	62.4	61	**63.9**	0.50	0.51	0.59	0.56	0.50	0.52	0.56	0.60	0.61	**0.62**
DS30	55.3	54.3	53.5	55.6	54.6	64.4	64	63.4	64.6	**65.6**	0.50	0.53	0.53	0.53	0.53	0.63	0.64	0.62	0.64	**0.65**

Significant values are in [bold].

**Table 30:** Accuracy and F-measure using FR4 with optimal number of labeled dataset (i.e., 60%).

Accuracy											F-measure									
ID	J48	RF	NB	K-star	AIRS	Y-J48	Y-RF	Y-NB	Y-Kstar	Y-AIRS	J48	RF	NB	K-star	AIRS	Y-J48	Y-RF	Y-NB	Y-Kstar	Y-AIRS
DS1	78.8	78.6	79.9	78.7	79.2	**87.4**	83.9	**87.4**	85.2	**87.4**	0.72	0.74	0.78	0.78	0.79	**0.83**	0.82	**0.83**	0.82	**0.83**
DS2	80.2	79.3	75.7	80.8	82.7	84.4	83.8	86.4	86.9	**89.8**	0.79	0.78	0.75	0.78	0.81	0.82	0.83	0.83	0.83	**0.87**
DS3	78.6	72.7	72.7	72.8	75.7	82.9	84.8	86.4	87.7	**89.9**	0.72	0.72	0.73	0.72	0.70	0.82	0.81	0.82	0.83	**0.87**
DS4	75.3	76.3	76.8	73.8	72.7	82.4	83.3	83.4	82.8	**88.5**	0.72	0.73	0.74	0.74	0.75	0.83	0.80	0.81	0.81	**0.87**
DS5	78.9	74.3	75.8	75.8	76.7	81.4	82.6	83.4	85.8	**87.9**	0.70	0.73	0.74	0.73	0.73	0.83	0.82	0.82	0.83	**0.86**
DS6	78.3	75.7	78.7	75.8	77.8	83.7	84.1	83.4	84.8	**87.8**	0.70	0.73	0.73	0.73	0.73	0.82	0.83	0.82	0.84	**0.86**
DS7	78.9	75.6	78.7	72.8	72.7	81.4	83	82.4	83.8	**85.9**	0.72	0.73	0.74	0.74	0.75	0.82	0.81	0.82	0.82	**0.84**
DS8	74.3	73.3	72.7	71.8	74.7	82.4	83.3	82.4	83	**86.7**	0.72	0.73	0.73	0.74	0.73	0.84	0.80	0.80	0.82	**0.85**
DS9	72.3	78.3	74.6	72.8	74.7	81.4	83.3	83.4	83.8	**89.8**	0.72	0.75	0.74	0.74	0.75	0.83	0.82	0.82	0.82	**0.84**
DS10	76.0	75.3	73.9	74.8	74.6	83.4	83	87.4	84.8	**87.8**	0.73	0.75	0.76	0.77	0.75	0.82	0.82	0.84	0.84	**0.86**
DS11	75.0	73.9	76.7	76.8	78.7	82.4	83.7	83.4	85.8	**87.8**	0.72	0.77	0.77	0.76	0.77	0.83	0.82	0.83	0.82	**0.85**
DS12	77.9	76.3	76.7	74.8	77.7	85.4	83.2	83.4	83	**86.9**	0.76	0.75	0.74	0.73	0.73	0.81	0.80	0.82	0.83	**0.86**
DS13	80.3	78.3	76.7	75.8	76.7	82.4	84.6	84.4	83.5	**88.9**	0.70	0.73	0.79	0.78	0.70	0.80	0.81	0.80	0.81	**0.86**
DS14	75.9	77.3	76.7	77.8	76.7	82.4	83	83.4	86.8	**88.9**	0.72	0.74	0.75	0.75	0.78	0.83	0.82	0.81	0.82	**0.86**
DS15	76.0	76.3	76.7	77.8	78.7	80.4	82	83.4	83.8	**86.9**	0.79	0.72	0.71	0.70	0.72	0.80	0.81	0.81	0.82	**0.83**
DS17	78.3	77.3	78.7	79.8	79.7	82.4	82.8	83.4	81.8	**87.9**	0.70	0.71	0.70	0.72	0.70	0.81	0.78	0.80	0.81	**0.84**
DS18	76.0	74.3	78.7	76.8	79.7	80.4	82.3	83.4	83.8	**84.1**	0.79	0.72	0.71	0.70	0.72	0.80	0.81	0.81	0.82	**0.83**
DS19	77.8	77.3	78.8	79.8	78.0	83.4	83	85.4	84	**87.6**	0.70	0.71	0.79	0.78	0.70	0.80	0.82	0.80	0.81	**0.82**
DS20	79.7	77.3	78.7	76.8	79.7	82.4	82.8	83.4	87.8	**87.9**	0.70	0.71	0.70	0.72	0.76	0.81	0.82	0.80	0.81	**0.84**
DS21	78.8	75.8	77.9	78.8	70.8	82.4	84	83.4	84.8	**88.2**	0.70	0.73	0.73	0.73	0.73	0.83	0.84	0.82	0.84	**0.86**
DS22	77.8	74.3	77.6	78.8	79.7	85.4	83	82.4	83.8	**87.9**	0.72	0.73	0.73	0.74	0.72	0.82	0.83	0.82	0.82	**0.85**
DS23	78.0	75.3	74.7	75.8	74.8	82.4	84.3	85.4	84.8	**87.8**	0.70	0.73	0.77	0.78	0.77	0.83	0.84	0.82	0.84	**0.87**
DS24	76.8	78.3	78.7	78.8	79.7	81.4	83	82.4	83.8	**88.2**	0.73	0.74	0.75	0.77	0.74	0.83	0.82	0.83	0.84	**0.85**
DS25	77.3	77.9	77.7	79.8	78.7	83.4	82	82.4	83	**87.5**	0.70	0.71	0.79	0.78	0.70	0.82	0.81	0.82	0.81	**0.83**
DS26	75.0	76.3	73.7	75.8	77.8	84.4	84	83.4	84.8	**88.2**	0.70	0.73	0.73	0.73	0.73	0.83	0.84	0.82	0.84	**0.85**
DS27	77.0	77.3	73.7	75.8	74.8	83.4	84	86.4	84.8	**89.2**	0.70	0.73	0.73	0.73	0.73	0.83	0.84	0.82	0.84	**0.85**
DS28	77.0	70.9	79.77	79.8	71.77	80.4	83	81.4	82	**88.9**	0.71	0.70	0.71	0.70	0.71	0.80	0.79	0.81	0.82	**0.83**
DS29	78.3	72.9	78.77	78.8	70.77	84.4	83	82.4	81	**89.9**	0.70	0.71	0.79	0.78	0.70	0.72	0.78	0.80	0.81	**0.82**
DS30	76.0	77.8	79.7	75.8	74.8	83.4	84	83.4	84.8	**89.8**	0.70	0.73	0.73	0.73	0.73	0.83	0.84	0.82	0.84	**0.85**

Significant values are in [bold].

**Table 31:** Accuracy and F-measure using FR4 with 100% labeled dataset.

Accuracy											F-measure									
ID	J48	RF	NB	K-star	AIRS	Y-J48	Y-RF	Y-NB	Y-Kstar	Y-AIRS	J48	RF	NB	K-star	AIRS	Y-J48	Y-RF	Y-NB	Y-Kstar	Y-AIRS
DS1	78.8	78.6	79.9	78.7	79.2	**87.4**	83.9	**87.4**	85.2	**87.4**	0.72	0.74	0.78	0.78	0.79	**0.83**	0.82	**0.83**	0.82	**0.83**
DS2	80.2	79.3	75.7	80.8	82.7	84.4	83.8	86.4	86.9	**89.8**	0.79	0.78	0.75	0.78	0.81	0.82	0.83	0.83	0.83	**0.87**
DS3	78.6	72.7	72.7	72.8	75.7	82.9	84.8	86.4	87.7	**89.9**	0.72	0.72	0.73	0.72	0.70	0.82	0.81	0.82	0.83	**0.87**
DS4	75.3	76.3	76.8	73.8	72.7	82.4	83.3	83.4	82.8	**88.5**	0.72	0.73	0.74	0.74	0.75	0.83	0.80	0.81	0.81	**0.87**
DS5	78.9	74.3	75.8	75.8	76.7	81.4	82.6	83.4	85.8	**87.9**	0.70	0.73	0.74	0.73	0.73	0.83	0.82	0.82	0.83	**0.86**
DS6	78.3	75.7	78.7	75.8	77.8	83.7	84.1	83.4	84.8	**87.8**	0.70	0.73	0.73	0.73	0.73	0.82	0.83	0.82	0.84	**0.86**
DS7	78.9	75.6	78.7	72.8	72.7	81.4	83	82.4	83.8	**85.9**	0.72	0.73	0.74	0.74	0.75	0.82	0.81	0.82	0.82	**0.84**
DS8	74.3	73.3	72.7	71.8	74.7	82.4	83.3	82.4	83	**86.7**	0.72	0.73	0.73	0.74	0.73	0.84	0.80	0.80	0.82	**0.85**
DS9	72.3	78.3	74.6	72.8	74.7	81.4	83.3	83.4	83.8	**89.8**	0.72	0.75	0.74	0.74	0.75	0.83	0.82	0.82	0.82	**0.84**
DS10	76.0	75.3	73.9	74.8	74.6	83.4	83	87.4	84.8	**87.8**	0.73	0.75	0.76	0.77	0.75	0.82	0.82	0.84	0.84	**0.86**
DS11	75.0	73.9	76.7	76.8	78.7	82.4	83.7	83.4	85.8	**87.8**	0.72	0.77	0.77	0.76	0.77	0.83	0.82	0.83	0.82	**0.85**
DS12	77.9	76.3	76.7	74.8	77.7	85.4	83.2	83.4	83	**86.9**	0.76	0.75	0.74	0.73	0.73	0.81	0.80	0.82	0.83	**0.86**
DS13	80.3	78.3	76.7	75.8	76.7	82.4	84.6	84.4	83.5	**88.9**	0.70	0.73	0.79	0.78	0.70	0.80	0.81	0.80	0.81	**0.86**
DS14	75.9	77.3	76.7	77.8	76.7	82.4	83	83.4	86.8	**88.9**	0.72	0.74	0.75	0.75	0.78	0.83	0.82	0.81	0.82	**0.86**
DS15	76.0	76.3	76.7	77.8	78.7	80.4	82	83.4	83.8	**86.9**	0.79	0.72	0.71	0.70	0.72	0.80	0.81	0.81	0.82	**0.83**
DS17	78.3	77.3	78.7	79.8	79.7	82.4	82.8	83.4	81.8	**87.9**	0.70	0.71	0.70	0.72	0.70	0.81	0.78	0.80	0.81	**0.84**
DS18	76.0	74.3	78.7	76.8	79.7	80.4	82.3	83.4	83.8	**84.1**	0.79	0.72	0.71	0.70	0.72	0.80	0.81	0.81	0.82	**0.83**
DS18	79.3	75.3	76.7	74.8	77.8	81.4	83	83.4	83	**83.8**	0.70	0.71	0.75	0.76	0.70	0.80	0.82	0.80	0.81	**0.82**
DS19	77.8	77.3	78.8	79.8	78.0	83.4	83	85.4	84	**87.6**	0.70	0.71	0.79	0.78	0.70	0.80	0.82	0.80	0.81	**0.82**
DS20	79.7	77.3	78.7	76.8	79.7	82.4	82.8	83.4	87.8	**87.9**	0.70	0.71	0.70	0.72	0.76	0.81	0.82	0.80	0.81	**0.84**
DS21	78.8	75.8	77.9	78.8	70.8	82.4	84	83.4	84.8	**88.2**	0.70	0.73	0.73	0.73	0.73	0.83	0.84	0.82	0.84	**0.86**
DS22	77.8	74.3	77.6	78.8	79.7	85.4	83	82.4	83.8	**87.9**	0.72	0.73	0.73	0.74	0.72	0.82	0.83	0.82	0.82	**0.85**
DS23	78.0	75.3	74.7	75.8	74.8	82.4	84.3	85.4	84.8	**87.8**	0.70	0.73	0.77	0.78	0.77	0.83	0.84	0.82	0.84	**0.87**
DS24	76.8	78.3	78.7	78.8	79.7	81.4	83	82.4	83.8	**88.2**	0.73	0.74	0.75	0.77	0.74	0.83	0.82	0.83	0.84	**0.85**
DS25	77.3	77.9	77.7	79.8	78.7	83.4	82	82.4	83	**87.5**	0.70	0.71	0.79	0.78	0.70	0.82	0.81	0.82	0.81	**0.83**
DS26	75.0	76.3	73.7	75.8	77.8	84.4	84	83.4	84.8	**88.2**	0.70	0.73	0.73	0.73	0.73	0.83	0.84	0.82	0.84	**0.85**
DS27	77.0	77.3	73.7	75.8	74.8	83.4	84	86.4	84.8	**89.2**	0.70	0.73	0.73	0.73	0.73	0.83	0.84	0.82	0.84	**0.85**
DS28	77.0	70.9	79.77	79.8	71.77	80.4	83	81.4	82	**88.9**	0.71	0.70	0.71	0.70	0.71	0.80	0.79	0.81	0.82	**0.83**
DS29	78.3	72.9	78.77	78.8	70.77	84.4	83	82.4	81	**89.9**	0.70	0.71	0.79	0.78	0.70	0.72	0.78	0.80	0.81	**0.82**
DS30	76.0	77.8	79.7	75.8	74.8	83.4	84	83.4	84.8	**89.8**	0.70	0.73	0.73	0.73	0.73	0.83	0.84	0.82	0.84	**0.85**

Significant values are in [bold].

**Table 32:** Accuracy and F-measure using FR5 with 5% labeled dataset.

Accuracy											F-measure									
ID	J48	RF	NB	K-star	AIRS	Y-J48	Y-RF	Y-NB	Y-Kstar	Y-AIRS	J48	RF	NB	K-star	AIRS	Y-J48	Y-RF	Y-NB	Y-Kstar	Y-AIRS
DS1	58.3	57.3	59.8	58.7	59.5	**63.4**	62.5	**63.4**	61.5	**63.4**	0.56	0.50	0.55	0.58	0.56	**0.62**	0.61	**0.62**	0.61	**0.62**
DS2	54.7	54.3	56.5	57.7	59.5	63.4	63.8	64.4	62.8	**64.8**	0.53	0.52	0.59	0.57	0.50	0.62	0.60	0.60	0.62	**0.63**
DS3	56.9	58.3	59.5	59.7	59.5	60.4	62	62.4	63	**63.9**	0.51	0.50	0.51	0.50	0.53	0.61	0.60	0.62	0.63	**0.64**
DS4	58.9	58.9	59.5	59.7	59.9	64.4	62.8	62.4	63.7	**64.8**	0.52	0.52	0.53	0.51	0.52	0.61	0.62	0.62	0.61	**0.63**
DS5	57.3	57.2	58.5	54.7	58.5	61.4	63.8	62.4	63.8	**63.9**	0.59	0.52	0.51	0.50	0.52	0.61	0.60	0.61	0.63	**0.64**
DS6	53.5	53.6	52.5	57.7	52.7	62.4	62	61.4	62.7	**64.0**	0.57	0.50	0.52	0.51	0.51	0.61	0.62	0.60	0.63	**0.64**
DS7	50.0	53.3	57.5	57.7	50.5	59.4	61.1	60.4	61.7	**64.7**	0.50	0.51	0.50	0.52	0.50	0.61	0.57	0.60	0.61	**0.62**
DS8	52.3	52.5	57.5	57.7	54.5	62.4	62	62.4	61	**63.4**	0.50	0.51	0.59	0.57	0.50	0.59	0.57	0.60	0.61	**0.62**
DS9	51.3	52.6	57.5	57.7	52.5	62.4	62.1	62.4	61	**62.7**	0.50	0.51	0.59	0.57	0.50	0.59	0.59	0.60	0.61	**0.63**
DS10	56.3	56.3	58.5	52.7	53.5	61.4	60.9	62.4	63.7	**62.9**	0.59	0.52	0.51	0.50	0.52	0.60	0.61	0.61	0.62	**0.63**
DS11	53.3	52.3	57.5	57.7	50.5	62.4	62	60.4	61.7	**63.0**	0.50	0.51	0.50	0.52	0.50	0.61	0.57	0.60	0.61	**0.62**
DS12	53.4	53.3	57.5	57.7	51.5	60.4	61	61.4	62	**62.9**	0.51	0.50	0.51	0.50	0.51	0.60	0.59	0.61	0.62	**0.63**
DS13	56.8	52.3	57.5	56.7	50.5	61.4	62	62.4	61	**63.4**	0.50	0.51	0.59	0.57	0.50	0.62	0.57	0.60	0.61	**0.63**
DS14	53.8	53.3	59.5	59.7	51.5	61.4	61	61.4	62	**63.8**	0.51	0.50	0.51	0.50	0.51	0.60	0.59	0.61	0.62	**0.62**
DS15	53.7	53.3	50.5	52.7	53.5	59.4	60	62.4	63.7	**64.2**	0.59	0.52	0.51	0.50	0.52	0.60	0.61	0.61	0.62	**0.64**
DS16	54.7	56.3	57.5	57.7	50.5	61.4	62	60.4	61.7	**62.9**	0.50	0.51	0.50	0.52	0.50	0.61	0.57	0.60	0.61	**0.64**
DS17	50.8	51.3	50.5	52.7	53.5	59.4	60	62.4	63.7	**63.9**	0.59	0.52	0.51	0.50	0.52	0.60	0.61	0.61	0.62	**0.63**
DS18	52.4	54.3	57.5	57.7	50.5	61.4	62	62.4	61	**62.8**	0.50	0.51	0.59	0.57	0.50	0.59	0.60	0.60	0.61	**0.62**
DS19	53.3	56.3	57.5	57.7	50.5	61.4	62	62.4	61	**62.9**	0.50	0.51	0.59	0.57	0.50	0.58	0.59	0.60	0.61	**0.62**
DS20	59.8	58.3	59.5	58.7	58.5	60.4	62.1	62.4	61.7	**63.8**	0.50	0.51	0.50	0.52	0.50	0.61	0.57	0.60	0.61	**0.64**
DS21	56.3	58.3	57.5	58.7	58.6	62.4	62.3	62.4	62	**63.9**	0.50	0.51	0.59	0.57	0.50	0.62	0.57	0.60	0.61	**0.63**
DS22	54.9	54.8	58.5	58.7	53.5	60.4	62.3	63.4	62.7	**63.7**	0.50	0.51	0.50	0.52	0.50	0.61	0.57	0.60	0.61	**0.64**
DS23	58.4	58.3	59.5	59.7	59.5	61.4	62.9	63.4	62.9	**64.8**	0.59	0.52	0.51	0.50	0.52	0.60	0.61	0.61	0.62	**0.63**
DS24	53.0	54.3	57.5	57.7	50.5	59.4	62	60.4	61.7	**63.7**	0.50	0.51	0.50	0.52	0.50	0.61	0.57	0.60	0.61	**0.64**
DS25	58.3	59.4	58.5	58.7	58.5	63.4	63.8	63.9	62	**66.8**	0.50	0.51	0.59	0.57	0.50	0.52	0.57	0.60	0.61	**0.62**
DS26	54.0	54.3	57.5	52.7	50.5	59.4	62	62.4	63.7	**63.5**	0.50	0.51	0.50	0.52	0.50	0.61	0.57	0.60	0.61	**0.64**
DS27	53.3	52.3	57.5	53.7	50.5	61.4	62	62.4	61	**63.7**	0.50	0.51	0.59	0.57	0.50	0.59	0.59	0.60	0.61	**0.62**
DS28	53.0	54.3	59.5	59.7	51.5	62.4	61	61.4	62	**65.9**	0.51	0.50	0.51	0.50	0.51	0.60	0.59	0.61	0.62	**0.63**
DS29	54.3	52.3	57.5	57.7	50.5	61.4	62	62.4	61	**63.2**	0.50	0.51	0.59	0.57	0.50	0.59	0.57	0.60	0.61	**0.62**
DS30	58.0	57.3	59.5	58.7	58.7	63.4	63.8	63.8	62.7	**65.8**	0.57	0.50	0.52	0.51	0.51	0.61	0.62	0.60	0.63	**0.64**

Significant values are in [bold].

**Table 33:** Accuracy and F-measure using FR5 with 10% labeled dataset.

Accuracy											F-measure									
ID	J48	RF	NB	K-star	AIRS	Y-J48	Y-RF	Y-NB	Y-Kstar	Y-AIRS	J48	RF	NB	K-star	AIRS	Y-J48	Y-RF	Y-NB	Y-Kstar	Y-AIRS
DS1	58.9	58.2	59.7	58.5	55.5	**63.8**	63.2	**63.8**	62.5	**63.8**	0.52	0.53	0.50	0.52	0.53	**0.61**	0.60	**0.61**	0.60	**0.61**
DS2	54.9	54.8	53.8	56.6	59.7	63.7	62.9	63.8	65.9	**66.2**	0.52	0.53	0.50	0.51	0.52	0.55	0.59	0.62	0.63	**0.65**
DS3	52.8	52.3	52.5	52.6	58.5	63.4	63.7	63.9	64.6	**64.1**	0.52	0.52	0.53	0.52	0.53	0.62	0.63	0.62	0.63	**0.65**
DS4	51.9	53.8	58.7	58.6	53.5	62.4	63.1	62.4	62.6	**63.8**	0.57	0.53	0.53	0.54	0.58	0.63	0.63	0.63	0.62	**0.64**
DS5	58.9	59.3	58.5	59.6	59.5	63.4	62.0	63.4	64.6	**67.8**	0.50	0.53	0.54	0.53	0.53	0.62	0.63	0.64	0.62	**0.68**
DS6	52.8	56.9	59.5	59.6	58.6	63.4	64.5	66.4	64.6	**66.8**	0.53	0.54	0.52	0.53	0.54	0.63	0.64	0.63	0.64	**0.67**
DS7	56.9	59.6	59.5	59.6	59.5	62.4	63.3	63.4	65.6	**65.9**	0.53	0.56	0.54	0.56	0.57	0.63	0.63	0.62	0.64	**0.66**
DS8	56.3	52.3	52.5	55.6	57.5	63.4	62.8	64.4	63.3	**65.8**	0.53	0.53	0.55	0.51	0.52	0.58	0.59	0.62	0.62	**0.65**
DS9	59.8	58.3	58.5	59.6	58.5	62.4	63.3	63.4	64.6	**66.6**	0.52	0.53	0.56	0.57	0.55	0.63	0.62	0.62	0.62	**0.66**
DS10	52.0	53.3	51.5	52.6	52.5	60.4	63.0	62.4	62.6	**63.6**	0.52	0.53	0.54	0.54	0.55	0.63	0.60	0.61	0.62	**0.63**
DS11	56.8	52.3	53.5	52.6	52.5	61.4	63.8	62.7	62.4	**65.9**	0.52	0.53	0.54	0.54	0.55	0.63	0.60	0.61	0.62	**0.63**
DS12	57.3	58.3	59.5	58.6	59.5	63.4	61.8	63.4	62.0	**63.9**	0.51	0.50	0.51	0.50	0.51	0.60	0.59	0.61	0.62	**0.63**
DS13	59.3	58.3	57.5	58.6	57.5	63.8	64.9	63.4	63.7	**67.6**	0.50	0.51	0.59	0.56	0.50	0.52	0.56	0.60	0.61	**0.62**
DS14	58.9	53.3	57.5	52.6	52.5	62.8	63.8	64.4	64.6	**66.7**	0.52	0.53	0.54	0.54	0.55	0.63	0.62	0.63	0.61	**0.65**
DS15	58.9	59.3	59.5	58.9	59.5	62.4	62.8	63.7	63.6	**66.9**	0.59	0.52	0.51	0.50	0.52	0.60	0.61	0.61	0.62	**0.65**
DS15	53.9	58.8	59.8	58.6	58.5	60.4	62.0	62.4	63.6	**63.9**	0.50	0.51	0.50	0.52	0.50	0.61	0.56	0.60	0.61	**0.64**
DS16	59.5	57.3	58.5	58.6	58.5	60.4	62.9	62.4	63.6	**64.1**	0.59	0.52	0.51	0.50	0.52	0.61	0.63	0.63	0.63	**0.67**
DS18	57.8	58.3	58.8	57.9	59.8	61.4	63.0	63.4	63.4	**64.1**	0.50	0.52	0.59	0.56	0.50	0.59	0.60	0.60	0.62	**0.63**
DS19	58.8	59.8	59.9	60.6	60.5	65.4	63.5	63.4	64.3	**65.8**	0.50	0.51	0.59	0.56	0.50	0.52	0.56	0.60	0.61	**0.62**
DS20	53.8	52.3	51.5	56.6	53.5	62.4	62.6	63.8	63.6	**64.9**	0.50	0.51	0.50	0.52	0.50	0.61	0.56	0.60	0.61	**0.64**
DS21	58.4	54.3	59.5	55.6	54.6	63.4	64.9	63.4	64.6	**65.9**	0.50	0.53	0.53	0.53	0.53	0.63	0.64	0.62	0.64	**0.65**
DS22	59.7	57.3	58.5	57.6	53.5	62.4	63.3	62.4	63.6	**64.8**	0.52	0.53	0.53	0.54	0.52	0.62	0.63	0.62	0.62	**0.65**
DS23	55.6	54.3	53.5	55.6	54.6	63.4	64.1	63.4	64.6	**65.9**	0.50	0.53	0.53	0.53	0.53	0.63	0.64	0.62	0.64	**0.65**
DS24	56.0	56.3	59.5	56.6	58.5	63.4	64.4	65.4	64.6	**66.6**	0.53	0.54	0.55	0.55	0.54	0.63	0.62	0.63	0.64	**0.65**
DS25	57.3	57.3	53.5	50.6	50.5	62.4	62.2	63.4	63.0	**65.2**	0.50	0.51	0.59	0.56	0.50	0.52	0.56	0.60	0.61	**0.62**
DS26	59.8	53.6	54.5	55.6	54.6	63.4	64.1	63.4	63.6	**64.9**	0.50	0.53	0.53	0.53	0.53	0.63	0.64	0.62	0.64	**0.65**
DS27	57.5	54.3	53.5	55.6	54.6	63.4	64.2	63.4	64.8	**65.2**	0.50	0.53	0.53	0.53	0.53	0.63	0.64	0.62	0.64	**0.65**
DS28	53.0	50.3	59.5	59.6	59.5	62.4	63.0	62.4	63.0	**65.3**	0.51	0.50	0.51	0.50	0.51	0.60	0.59	0.61	0.62	**0.63**
DS29	58.3	56.3	56.9	59.6	50.5	63.4	62.9	62.4	61.0	**63.9**	0.50	0.51	0.59	0.56	0.50	0.52	0.56	0.60	0.61	**0.62**
DS30	55.3	54.3	53.5	55.6	54.6	64.4	64.0	63.4	64.6	**65.6**	0.50	0.53	0.53	0.53	0.53	0.63	0.64	0.62	0.64	**0.65**

Significant values are in [bold].

**Table 34:** Accuracy and F-measure using FR5 with optimal number of labeled dataset (i.e., 60%)

Accuracy											F-measure									
ID	J48	RF	NB	K-star	AIRS	Y-J48	Y-RF	Y-NB	Y-Kstar	Y-AIRS	J48	RF	NB	K-star	AIRS	Y-J48	Y-RF	Y-NB	Y-Kstar	Y-AIRS
DS1	78.8	78.6	79.9	78.7	79.2	**87.4**	83.9	**87.4**	85.2	**87.4**	0.72	0.74	0.78	0.78	0.79	**0.83**	0.82	**0.83**	0.82	**0.83**
DS2	80.2	79.3	75.7	80.8	82.7	84.4	83.8	86.4	86.9	**88.8**	0.79	0.78	0.75	0.78	0.81	0.82	0.83	0.83	0.83	**0.87**
DS3	78.6	72.7	72.7	72.8	75.7	82.9	84.8	86.4	85.7	**87.9**	0.72	0.72	0.73	0.72	0.70	0.82	0.81	0.82	0.83	**0.87**
DS4	75.3	76.3	76.8	73.8	72.7	82.4	83.3	83.4	82.8	**88.9**	0.72	0.73	0.74	0.74	0.75	0.83	0.80	0.81	0.81	**0.87**
DS5	78.9	74.3	75.8	75.8	76.7	81.4	82.6	83.4	85.8	**89.9**	0.70	0.73	0.74	0.73	0.73	0.83	0.82	0.82	0.83	**0.86**
DS6	78.3	75.7	78.7	75.8	77.8	83.7	84.1	83.4	84.8	**88.8**	0.70	0.73	0.73	0.73	0.73	0.82	0.83	0.82	0.84	**0.86**
DS7	78.9	75.6	78.7	72.8	72.7	81.4	83	82.4	83.8	**89.9**	0.72	0.73	0.74	0.74	0.75	0.82	0.81	0.82	0.82	**0.84**
DS8	74.3	73.3	72.7	71.8	74.7	82.4	83.3	82.4	83	**89.7**	0.72	0.73	0.73	0.74	0.73	0.84	0.80	0.80	0.82	**0.85**
DS9	72.3	78.3	74.6	72.8	74.7	83.4	83.3	83.4	83.8	**89.8**	0.72	0.75	0.74	0.74	0.75	0.83	0.82	0.82	0.82	**0.84**
DS10	76.0	75.3	73.9	74.8	74.6	83.9	86	87.4	84.8	**87.8**	0.73	0.75	0.76	0.77	0.75	0.82	0.82	0.84	0.84	**0.86**
DS11	75.0	73.9	76.7	76.8	78.7	82.4	83.7	83.4	85.8	**87.8**	0.72	0.77	0.77	0.76	0.77	0.83	0.82	0.83	0.82	**0.85**
DS12	77.9	76.3	76.7	74.8	77.7	85.4	87.2	83.4	83	**88.9**	0.76	0.75	0.74	0.73	0.73	0.81	0.80	0.82	0.83	**0.86**
DS13	80.3	78.3	76.7	75.8	76.7	82.4	84.6	84.8	83.5	**88.7**	0.70	0.73	0.79	0.78	0.70	0.80	0.81	0.80	0.81	**0.86**
DS14	75.9	77.3	76.7	77.8	76.7	82.4	83	83.4	86.8	**88.9**	0.72	0.74	0.75	0.75	0.78	0.83	0.82	0.81	0.82	**0.86**
DS15	76.0	76.3	76.7	77.8	78.7	80.4	82	86.4	86.8	**87.9**	0.79	0.72	0.71	0.70	0.72	0.80	0.81	0.81	0.82	**0.85**
DS17	78.3	77.3	78.7	79.8	79.7	82.4	84.8	87.4	87.8	**88.9**	0.70	0.71	0.70	0.72	0.70	0.81	0.78	0.80	0.81	**0.84**
DS18	76.0	74.3	78.7	76.8	79.7	80.4	84.3	86.4	85.8	**87.1**	0.79	0.72	0.71	0.70	0.72	0.80	0.81	0.81	0.82	**0.85**
DS18	79.3	75.3	76.7	74.8	77.8	81.4	83	83.4	83	**83.8**	0.70	0.71	0.75	0.76	0.70	0.80	0.82	0.80	0.81	**0.82**
DS19	77.8	77.3	78.8	79.8	78.0	83.4	83	85.4	84	**87.6**	0.70	0.71	0.79	0.78	0.70	0.80	0.82	0.80	0.81	**0.82**
DS20	79.7	77.3	78.7	76.8	79.7	82.9	86.8	86.4	87.8	**89.9**	0.70	0.71	0.70	0.72	0.76	0.81	0.82	0.80	0.81	**0.84**
DS21	79.8	75.8	77.9	79.8	73.8	82.4	84.8	87.4	87.8	**89.2**	0.70	0.73	0.73	0.73	0.73	0.83	0.84	0.82	0.84	**0.86**
DS22	78.8	74.3	77.6	78.8	79.7	85.4	83	82.4	83.8	**87.9**	0.72	0.73	0.73	0.74	0.72	0.82	0.83	0.82	0.82	**0.85**
DS23	79.0	75.3	74.7	75.8	74.8	82.4	84.3	85.4	84.8	**87.8**	0.70	0.73	0.77	0.78	0.77	0.83	0.84	0.82	0.84	**0.87**
DS24	79.8	79.3	79.7	79.8	79.7	81.4	83	82.4	83.8	**88.2**	0.73	0.74	0.75	0.77	0.74	0.83	0.82	0.83	0.84	**0.85**
DS25	77.3	77.9	77.7	79.8	78.7	83.4	82	82.4	83	**87.5**	0.70	0.71	0.79	0.78	0.70	0.82	0.81	0.82	0.81	**0.83**
DS26	75.0	76.3	73.7	75.8	77.8	84.4	84	83.4	84.8	**88.2**	0.70	0.73	0.73	0.73	0.73	0.83	0.84	0.82	0.84	**0.85**
DS27	77.0	77.3	73.7	75.8	74.8	83.4	84	86.4	84.8	**89.2**	0.70	0.73	0.73	0.73	0.73	0.83	0.84	0.82	0.84	**0.85**
DS28	77.0	70.9	79.77	79.8	71.77	80.4	83	81.4	82	**88.9**	0.71	0.70	0.71	0.70	0.71	0.80	0.79	0.81	0.82	**0.83**
DS29	78.3	72.9	78.77	78.8	70.77	84.4	83	82.4	81	**89.9**	0.70	0.71	0.79	0.78	0.70	0.72	0.78	0.80	0.81	**0.82**
DS30	76.0	77.8	79.7	75.8	74.8	83.4	84	83.4	84.8	**89.8**	0.70	0.73	0.73	0.73	0.73	0.83	0.84	0.82	0.84	**0.85**

Significant values are in [bold].

**Table 35:** Accuracy and F-measure using FR5 with 100% labeled dataset.

Accuracy											F-measure									
ID	J48	RF	NB	K-star	AIRS	Y-J48	Y-RF	Y-NB	Y-Kstar	Y-AIRS	J48	RF	NB	K-star	AIRS	Y-J48	Y-RF	Y-NB	Y-Kstar	Y-AIRS
DS1	78.8	78.6	79.9	78.7	79.2	**87.4**	83.9	**87.4**	85.2	**87.4**	0.72	0.74	0.78	0.78	0.79	**0.83**	0.82	**0.83**	0.82	**0.83**
DS2	80.2	79.3	75.7	80.8	82.7	84.4	83.8	86.4	86.9	**88.8**	0.79	0.78	0.75	0.78	0.81	0.82	0.83	0.83	0.83	**0.87**
DS3	78.6	72.7	72.7	72.8	75.7	82.9	84.8	86.4	85.7	**87.9**	0.72	0.72	0.73	0.72	0.70	0.82	0.81	0.82	0.83	**0.87**
DS4	75.3	76.3	76.8	73.8	72.7	82.4	83.3	83.4	82.8	**88.9**	0.72	0.73	0.74	0.74	0.75	0.83	0.80	0.81	0.81	**0.87**
DS5	78.9	74.3	75.8	75.8	76.7	81.4	82.6	83.4	85.8	**89.9**	0.70	0.73	0.74	0.73	0.73	0.83	0.82	0.82	0.83	**0.86**
DS6	78.3	75.7	78.7	75.8	77.8	83.7	84.1	83.4	84.8	**88.8**	0.70	0.73	0.73	0.73	0.73	0.82	0.83	0.82	0.84	**0.86**
DS7	78.9	75.6	78.7	72.8	72.7	81.4	83	82.4	83.8	**89.9**	0.72	0.73	0.74	0.74	0.75	0.82	0.81	0.82	0.82	**0.84**
DS8	74.3	73.3	72.7	71.8	74.7	82.4	83.3	82.4	83	**89.7**	0.72	0.73	0.73	0.74	0.73	0.84	0.80	0.80	0.82	**0.85**
DS9	72.3	78.3	74.6	72.8	74.7	83.4	83.3	83.4	83.8	**89.8**	0.72	0.75	0.74	0.74	0.75	0.83	0.82	0.82	0.82	**0.84**
DS10	76.0	75.3	73.9	74.8	74.6	83.9	86	87.4	84.8	**87.8**	0.73	0.75	0.76	0.77	0.75	0.82	0.82	0.84	0.84	**0.86**
DS11	75.0	73.9	76.7	76.8	78.7	82.4	83.7	83.4	85.8	**87.8**	0.72	0.77	0.77	0.76	0.77	0.83	0.82	0.83	0.82	**0.85**
DS12	77.9	76.3	76.7	74.8	77.7	85.4	87.2	83.4	83	**88.9**	0.76	0.75	0.74	0.73	0.73	0.81	0.80	0.82	0.83	**0.86**
DS13	80.3	78.3	76.7	75.8	76.7	82.4	84.6	84.8	83.5	**88.7**	0.70	0.73	0.79	0.78	0.70	0.80	0.81	0.80	0.81	**0.86**
DS14	75.9	77.3	76.7	77.8	76.7	82.4	83	83.4	86.8	**88.9**	0.72	0.74	0.75	0.75	0.78	0.83	0.82	0.81	0.82	**0.86**
DS15	76.0	76.3	76.7	77.8	78.7	80.4	82	86.4	86.8	**87.9**	0.79	0.72	0.71	0.70	0.72	0.80	0.81	0.81	0.82	**0.85**
DS17	78.3	77.3	78.7	79.8	79.7	82.4	84.8	87.4	87.8	**88.9**	0.70	0.71	0.70	0.72	0.70	0.81	0.78	0.80	0.81	**0.84**
DS18	76.0	74.3	78.7	76.8	79.7	80.4	84.3	86.4	85.8	**87.1**	0.79	0.72	0.71	0.70	0.72	0.80	0.81	0.81	0.82	**0.85**
DS19	77.8	77.3	78.8	79.8	78.0	83.4	83	85.4	84	**87.6**	0.70	0.71	0.79	0.78	0.70	0.80	0.82	0.80	0.81	**0.82**
DS20	79.7	77.3	78.7	76.8	79.7	82.9	86.8	86.4	87.8	**89.9**	0.70	0.71	0.70	0.72	0.76	0.81	0.82	0.80	0.81	**0.84**
DS21	79.8	75.8	77.9	79.8	73.8	82.4	84.8	87.4	87.8	**89.2**	0.70	0.73	0.73	0.73	0.73	0.83	0.84	0.82	0.84	**0.86**
DS22	78.8	74.3	77.6	78.8	79.7	85.4	83	82.4	83.8	**87.9**	0.72	0.73	0.73	0.74	0.72	0.82	0.83	0.82	0.82	**0.85**
DS23	79.0	75.3	74.7	75.8	74.8	82.4	84.3	85.4	84.8	**87.8**	0.70	0.73	0.77	0.78	0.77	0.83	0.84	0.82	0.84	**0.87**
DS24	79.8	79.3	79.7	79.8	79.7	81.4	83	82.4	83.8	**88.2**	0.73	0.74	0.75	0.77	0.74	0.83	0.82	0.83	0.84	**0.85**
DS25	77.3	77.9	77.7	79.8	78.7	83.4	82	82.4	83	**87.5**	0.70	0.71	0.79	0.78	0.70	0.82	0.81	0.82	0.81	**0.83**
DS26	75.0	76.3	73.7	75.8	77.8	84.4	84	83.4	84.8	**88.2**	0.70	0.73	0.73	0.73	0.73	0.83	0.84	0.82	0.84	**0.85**
DS27	77.0	77.3	73.7	75.8	74.8	83.4	84	86.4	84.8	**89.2**	0.70	0.73	0.73	0.73	0.73	0.83	0.84	0.82	0.84	**0.85**
DS28	77.0	70.9	79.77	79.8	71.77	80.4	83	81.4	82	**88.9**	0.71	0.70	0.71	0.70	0.71	0.80	0.79	0.81	0.82	**0.83**
DS29	78.3	72.9	78.77	78.8	70.77	84.4	83	82.4	81	**89.9**	0.70	0.71	0.79	0.78	0.70	0.72	0.78	0.80	0.81	**0.82**
DS30	76.0	77.8	79.7	75.8	74.8	83.4	84	83.4	84.8	**89.8**	0.70	0.73	0.73	0.73	0.73	0.83	0.84	0.82	0.84	**0.85**

Significant values are in [bold].

**Table 36:** Accuracy and F-measure using FR6 with 5% labeled dataset.

Accuracy											F-measure									
ID	J48	RF	NB	K-star	AIRS	Y-J48	Y-RF	Y-NB	Y-Kstar	Y-AIRS	J48	RF	NB	K-star	AIRS	Y-J48	Y-RF	Y-NB	Y-Kstar	Y-AIRS
DS1	58.3	59.3	59.2	59.7	59.8	**63.8**	63.5	**63.8**	62.5	**63.8**	0.58	0.53	0.55	0.58	0.58	**0.63**	0.62	**0.63**	0.61	**0.63**
DS2	54.7	54.3	56.5	57.7	59.5	65.4	65.1	63.4	63.8	**66.8**	0.53	0.52	0.59	0.57	0.50	0.62	0.61	0.61	0.63	**0.64**
DS3	57.9	58.8	59.7	59.7	59.5	62.4	62.8	62.4	63	**64.0**	0.51	0.50	0.51	0.50	0.53	0.61	0.60	0.62	0.63	**0.64**
DS4	59.8	58.9	59.5	59.7	59.9	64.4	63.8	62.4	63.7	**64.7**	0.52	0.52	0.53	0.51	0.52	0.61	0.62	0.62	0.61	**0.63**
DS5	57.3	57.2	58.5	54.7	58.5	61.4	63.8	62.4	63.8	**65.2**	0.59	0.52	0.51	0.50	0.52	0.61	0.60	0.61	0.63	**0.64**
DS6	53.5	53.6	52.5	57.7	52.7	62.4	62	61.4	62.7	**63.9**	0.57	0.50	0.52	0.51	0.51	0.61	0.62	0.60	0.63	**0.64**
DS7	50.0	53.3	57.5	57.7	50.5	59.4	61.1	60.4	61.7	**63.7**	0.50	0.51	0.50	0.52	0.50	0.61	0.57	0.60	0.61	**0.62**
DS8	52.3	52.5	57.5	57.7	54.5	62.4	62	62.4	61	**63.9**	0.50	0.51	0.59	0.57	0.50	0.59	0.57	0.60	0.61	**0.62**
DS9	51.3	52.6	57.5	57.7	52.5	62.4	62.1	62.4	61	**62.9**	0.50	0.51	0.59	0.57	0.50	0.59	0.59	0.60	0.61	**0.63**
DS10	56.3	56.3	58.5	52.7	53.5	61.4	60.9	62.4	63.7	**63.9**	0.59	0.52	0.51	0.50	0.52	0.60	0.61	0.61	0.62	**0.63**
DS11	59.3	58.9	59.5	59.7	59.8	62.4	62	60.4	61.7	**63.8**	0.50	0.51	0.50	0.52	0.50	0.61	0.57	0.60	0.61	**0.62**
DS12	58.4	53.9	59.5	57.7	59.8	62.4	63	63.4	62	**63.9**	0.51	0.50	0.51	0.50	0.51	0.60	0.59	0.61	0.62	**0.63**
DS13	56.8	52.3	57.5	56.7	50.5	61.4	62	62.4	61	**63.9**	0.50	0.51	0.59	0.57	0.50	0.62	0.57	0.60	0.61	**0.63**
DS14	59.8	59.3	59.9	59.7	51.5	61.4	61	61.4	62	**64.8**	0.51	0.50	0.51	0.50	0.51	0.60	0.59	0.61	0.62	**0.62**
DS15	59.8	53.3	50.5	59.7	59.5	61.4	60	62.4	63.7	**64.2**	0.59	0.52	0.51	0.50	0.52	0.60	0.61	0.61	0.62	**0.64**
DS16	59.8	59.3	59.5	59.7	60.5	63.4	62	60.4	61.7	**63.8**	0.50	0.51	0.50	0.52	0.50	0.61	0.57	0.60	0.61	**0.64**
DS17	58.8	51.3	50.5	52.7	53.5	61.4	60.5	62.4	63.9	**63.9**	0.59	0.52	0.51	0.50	0.52	0.60	0.61	0.61	0.62	**0.63**
DS18	52.4	54.3	57.5	57.7	50.5	61.4	62	62.4	61	**62.8**	0.50	0.51	0.59	0.57	0.50	0.59	0.60	0.60	0.61	**0.62**
DS19	53.3	56.3	57.5	57.7	50.5	61.4	62	62.4	61	**62.9**	0.50	0.51	0.59	0.57	0.50	0.58	0.59	0.60	0.61	**0.62**
DS20	59.9	58.3	59.5	58.7	58.5	62.4	62.1	62.4	61.7	**64.8**	0.50	0.51	0.50	0.52	0.50	0.61	0.57	0.60	0.61	**0.64**
DS21	59.3	59.3	59.5	59.7	59.6	62.4	62.3	62.4	62	**64.9**	0.50	0.51	0.59	0.57	0.50	0.62	0.57	0.60	0.61	**0.63**
DS22	54.9	54.8	58.5	58.7	53.5	60.4	62.3	63.4	62.7	**63.7**	0.50	0.51	0.50	0.52	0.50	0.61	0.57	0.60	0.61	**0.64**
DS23	58.4	58.3	59.5	59.7	59.5	61.4	62.9	63.4	62.9	**64.8**	0.59	0.52	0.51	0.50	0.52	0.60	0.61	0.61	0.62	**0.63**
DS24	53.0	54.3	57.5	57.7	50.5	59.4	62	60.4	61.7	**65.7**	0.50	0.51	0.50	0.52	0.50	0.61	0.57	0.60	0.61	**0.64**
DS25	58.3	59.4	58.5	58.7	58.5	63.4	63.8	63.9	62	**66.2**	0.50	0.51	0.59	0.57	0.50	0.52	0.57	0.60	0.61	**0.62**
DS26	54.0	54.3	57.5	52.7	50.5	59.4	62	62.4	63.7	**63.5**	0.50	0.51	0.50	0.52	0.50	0.61	0.57	0.60	0.61	**0.64**
DS27	53.3	52.3	57.5	53.7	50.5	61.4	62	62.4	61	**63.7**	0.50	0.51	0.59	0.57	0.50	0.59	0.59	0.60	0.61	**0.62**
DS28	53.0	54.3	59.5	59.7	51.5	62.4	61	61.4	62	**65.9**	0.51	0.50	0.51	0.50	0.51	0.60	0.59	0.61	0.62	**0.63**
DS29	54.3	52.3	57.5	57.7	50.5	61.4	62	62.4	61	**65.2**	0.50	0.51	0.59	0.57	0.50	0.59	0.57	0.60	0.61	**0.62**
DS30	58.0	57.3	59.5	58.7	58.7	63.4	63.8	63.8	62.7	**65.8**	0.57	0.50	0.52	0.51	0.51	0.61	0.62	0.60	0.63	**0.64**

Significant values are in [bold].

**Table 37:** Accuracy and F-measure using FR6 with 10% labeled dataset.

Accuracy											F-measure									
ID	J48	RF	NB	K-star	AIRS	Y-J48	Y-RF	Y-NB	Y-Kstar	Y-AIRS	J48	RF	NB	K-star	AIRS	Y-J48	Y-RF	Y-NB	Y-Kstar	Y-AIRS
DS1	58.9	58.2	59.7	58.5	55.5	**63.8**	63.2	**63.8**	62.5	**63.8**	0.52	0.53	0.50	0.52	0.53	**0.61**	0.60	**0.61**	0.60	**0.61**
DS2	54.9	54.8	53.8	56.6	59.7	63.7	62.9	63.8	65.9	**67.2**	0.52	0.53	0.50	0.51	0.52	0.55	0.59	0.62	0.63	**0.65**
DS3	58.8	58.3	57.5	58.6	58.5	63.4	63.7	63.9	64.6	**65.9**	0.52	0.52	0.53	0.52	0.53	0.62	0.63	0.62	0.63	**0.65**
DS4	58.9	59.8	58.7	58.6	53.5	62.4	63.7	62.4	62.6	**64.9**	0.57	0.53	0.53	0.54	0.58	0.63	0.63	0.63	0.62	**0.65**
DS5	59.9	59.3	58.5	59.6	59.5	63.4	62	63.8	65.6	**68.9**	0.50	0.53	0.54	0.53	0.53	0.64	0.63	0.67	0.65	**0.69**
DS6	59.8	56.9	59.8	59.6	58.6	63.4	64.5	66.4	67.6	**67.8**	0.53	0.54	0.52	0.53	0.54	0.63	0.64	0.63	0.64	**0.67**
DS7	59.9	59.8	59.5	59.6	59.5	62.4	63.8	63.6	66.6	**67.9**	0.53	0.56	0.54	0.56	0.57	0.63	0.63	0.62	0.64	**0.66**
DS8	56.3	52.3	52.5	55.6	57.5	63.4	62.8	64.4	63.3	**68.8**	0.53	0.53	0.55	0.51	0.52	0.58	0.59	0.62	0.62	**0.65**
DS9	60.8	60.3	58.5	60.6	60.5	67.4	67.3	67.4	65.6	**68.9**	0.52	0.53	0.56	0.57	0.55	0.63	0.62	0.62	0.62	**0.66**
DS10	58.9	58.3	57.5	57.8	58.5	62.4	63	62.4	64.6	**68.7**	0.52	0.53	0.54	0.54	0.55	0.63	0.60	0.61	0.62	**0.68**
DS11	56.8	52.3	53.5	52.6	52.5	61.4	63.8	64.7	64.8	**68.9**	0.52	0.53	0.54	0.54	0.55	0.63	0.60	0.61	0.62	**0.67**
DS12	60.3	60.3	60.5	60.6	61.5	67.4	67.8	67.4	67.8	**63.9**	0.51	0.50	0.51	0.50	0.51	0.60	0.59	0.61	0.62	**0.63**
DS13	59.3	58.3	57.5	58.6	57.5	65.8	65.9	65.4	65.7	**67.6**	0.50	0.51	0.59	0.56	0.50	0.52	0.56	0.60	0.61	**0.62**
DS14	58.9	53.3	57.5	52.6	52.5	62.8	63.8	65.8	64.8	**68.7**	0.52	0.53	0.54	0.54	0.55	0.63	0.62	0.63	0.61	**0.65**
DS15	58.9	59.3	59.5	58.9	59.5	62.4	62.8	63.7	63.6	**66.9**	0.59	0.52	0.51	0.50	0.52	0.60	0.61	0.61	0.62	**0.65**
DS15	53.9	58.8	59.8	58.6	58.5	60.4	62	62.4	63.6	**63.9**	0.50	0.51	0.50	0.52	0.50	0.61	0.56	0.60	0.61	**0.64**
DS16	59.5	57.3	58.5	58.6	58.5	60.4	62.9	62.4	63.6	**64.1**	0.59	0.52	0.51	0.50	0.52	0.61	0.63	0.63	0.63	**0.67**
DS18	57.8	58.3	58.8	57.9	59.8	61.4	63	63.4	63.4	**64.1**	0.50	0.52	0.59	0.56	0.50	0.59	0.6	0.60	0.62	**0.63**
DS19	58.8	59.8	59.9	60.6	60.5	65.4	63.5	63.4	64.3	**65.8**	0.50	0.51	0.59	0.56	0.50	0.52	0.56	0.60	0.61	**0.62**
DS20	53.8	52.3	51.5	56.6	53.5	62.4	62.6	63.8	63.6	**64.9**	0.50	0.51	0.50	0.52	0.50	0.61	0.56	0.60	0.61	**0.64**
DS21	58.4	54.3	59.5	55.6	54.6	63.4	64.9	63.4	64.6	**65.9**	0.50	0.53	0.53	0.53	0.53	0.63	0.64	0.62	0.64	**0.65**
DS22	59.7	57.3	58.5	57.6	53.5	62.4	63.3	62.4	63.6	**64.8**	0.52	0.53	0.53	0.54	0.52	0.62	0.63	0.62	0.62	**0.65**
DS23	55.6	54.3	53.5	55.6	54.6	63.4	64.1	63.4	64.6	**65.9**	0.50	0.53	0.53	0.53	0.53	0.63	0.64	0.62	0.64	**0.65**
DS24	56.0	56.3	59.5	56.6	58.5	63.4	64.4	65.4	64.6	**66.6**	0.53	0.54	0.55	0.55	0.54	0.63	0.62	0.63	0.64	**0.65**
DS25	57.3	57.3	53.5	50.6	50.5	62.4	62.2	63.4	63	**65.2**	0.50	0.51	0.59	0.56	0.50	0.52	0.56	0.60	0.61	**0.62**
DS26	59.8	53.6	54.5	55.6	54.6	63.4	64.1	63.4	63.6	**64.9**	0.50	0.53	0.53	0.53	0.53	0.63	0.64	0.62	0.64	**0.65**
DS27	57.5	54.3	53.5	55.6	54.6	63.4	64.2	63.4	64.8	**65.2**	0.50	0.53	0.53	0.53	0.53	0.63	0.64	0.62	0.64	**0.65**
DS28	53.0	50.3	59.5	59.6	59.5	62.4	63	62.4	63	**65.3**	0.51	0.50	0.51	0.50	0.51	0.60	0.59	0.61	0.62	**0.63**
DS29	58.3	56.3	56.9	59.6	50.5	63.4	62.9	62.4	61	**63.9**	0.50	0.51	0.59	0.56	0.50	0.52	0.56	0.60	0.61	**0.62**
DS30	55.3	54.3	53.5	55.6	54.6	64.4	64	63.4	64.6	**65.6**	0.50	0.53	0.53	0.53	0.53	0.63	0.64	0.62	0.64	**0.65**

Significant values are in [bold].

**Table 38:** Accuracy and F-measure using FR6 with optimal number of labeled dataset (i.e., 60%).

Accuracy											F-measure									
ID	J48	RF	NB	K-star	AIRS	Y-J48	Y-RF	Y-NB	Y-Kstar	Y-AIRS	J48	RF	NB	K-star	AIRS	Y-J48	Y-RF	Y-NB	Y-Kstar	Y-AIRS
DS1	89.9	89.6	89.9	89.8	89.2	**98.4**	93.9	**98.4**	95.2	**98.4**	0.82	0.84	0.89	0.89	0.89	**0.93**	0.92	**0.93**	0.92	**0.93**
DS2	89.2	89.3	85.8	90.9	92.8	94.4	93.9	96.4	96.9	**98.9**	0.89	0.89	0.85	0.89	0.91	0.92	0.93	0.93	0.93	**0.98**
DS3	89.6	82.8	82.8	82.9	85.8	92.9	94.9	96.4	95.8	**97.9**	0.82	0.82	0.83	0.82	0.80	0.92	0.91	0.92	0.93	**0.98**
DS4	85.3	86.3	86.9	83.9	82.8	92.4	93.3	93.4	92.9	**98.2**	0.82	0.83	0.84	0.84	0.85	0.93	0.90	0.91	0.91	**0.98**
DS5	89.9	84.3	85.9	85.9	86.8	91.4	92.6	93.4	95.9	**97.1**	0.80	0.83	0.84	0.83	0.83	0.93	0.92	0.92	0.93	**0.96**
DS6	89.3	85.8	89.8	85.9	88.9	93.8	94.1	93.4	94.9	**96.8**	0.80	0.83	0.83	0.83	0.83	0.92	0.93	0.92	0.94	**0.96**
DS7	89.9	85.6	89.8	82.9	82.8	91.4	93	92.4	93.9	**95.5**	0.82	0.83	0.84	0.84	0.85	0.92	0.91	0.92	0.92	**0.94**
DS8	84.3	83.3	82.8	81.9	84.8	92.4	93.3	92.4	93	**95.8**	0.82	0.83	0.83	0.84	0.83	0.94	0.90	0.90	0.92	**0.95**
DS9	82.3	89.3	84.6	82.9	84.8	93.4	93.3	93.4	93.9	**96.9**	0.82	0.85	0.84	0.84	0.85	0.93	0.92	0.92	0.92	**0.94**
DS10	86.0	85.3	83.9	84.9	84.6	93.9	96	98.4	94.9	**98.9**	0.83	0.85	0.86	0.88	0.85	0.92	0.92	0.94	0.94	**0.96**
DS11	85.0	83.9	86.8	86.9	89.8	92.4	93.8	93.4	95.9	**98.9**	0.82	0.88	0.88	0.86	0.88	0.93	0.92	0.93	0.92	**0.95**
DS12	88.9	86.3	86.8	84.9	88.8	95.4	98.2	93.4	93	**97.9**	0.86	0.85	0.84	0.83	0.83	0.91	0.90	0.92	0.93	**0.96**
DS13	90.3	89.3	86.8	85.9	86.8	92.4	94.6	94.9	93.5	**97.8**	0.80	0.83	0.89	0.89	0.80	0.90	0.91	0.90	0.91	**0.96**
DS14	85.9	88.3	86.8	88.9	86.8	92.4	93	93.4	96.9	**98.9**	0.82	0.84	0.85	0.85	0.89	0.93	0.92	0.91	0.92	**0.96**
DS15	86.0	86.3	86.8	88.9	89.8	90.4	92	96.4	96.9	**98.9**	0.89	0.82	0.81	0.80	0.82	0.90	0.91	0.91	0.92	**0.95**
DS16	89.3	88.3	89.8	89.9	89.8	92.4	94.9	98.4	98.9	**99.9**	0.80	0.81	0.80	0.82	0.80	0.91	0.89	0.90	0.91	**0.94**
DS17	86.0	84.3	89.8	86.9	89.8	90.4	94.3	96.4	95.9	**98.1**	0.89	0.82	0.81	0.80	0.82	0.90	0.91	0.91	0.92	**0.95**
DS18	89.3	85.3	86.8	84.9	88.9	91.4	93	93.4	93	**93.9**	0.80	0.81	0.85	0.86	0.80	0.90	0.92	0.90	0.91	**0.92**
DS19	88.9	88.3	89.9	89.9	89.0	93.4	93	95.4	94	**97.6**	0.80	0.81	0.89	0.89	0.80	0.90	0.92	0.90	0.91	**0.92**
DS20	89.8	88.3	89.8	86.9	89.8	92.9	96.9	96.4	98.9	**99.9**	0.80	0.81	0.80	0.82	0.86	0.91	0.92	0.90	0.91	**0.94**
DS21	89.9	85.9	88.9	89.9	83.9	92.4	94.9	98.4	98.9	**99.2**	0.80	0.83	0.83	0.83	0.83	0.93	0.94	0.92	0.94	**0.96**
DS22	89.9	84.3	88.6	89.9	89.8	95.4	93	92.4	93.9	**95.9**	0.82	0.83	0.83	0.84	0.82	0.92	0.93	0.92	0.92	**0.95**
DS23	89.0	85.3	84.8	85.9	84.9	92.4	94.3	95.4	94.9	**95.9**	0.80	0.83	0.88	0.89	0.88	0.93	0.94	0.92	0.94	**0.98**
DS24	89.9	89.3	89.8	89.9	89.8	91.4	93	92.4	93.9	**95.2**	0.83	0.84	0.85	0.88	0.84	0.93	0.92	0.93	0.94	**0.95**
DS25	88.3	88.9	88.8	89.9	89.8	93.4	92	92.4	93	**96.5**	0.80	0.81	0.89	0.89	0.80	0.92	0.91	0.92	0.91	**0.93**
DS26	85.0	86.3	83.8	85.9	88.9	94.4	94	93.4	94.9	**95.2**	0.80	0.83	0.83	0.83	0.83	0.93	0.94	0.92	0.94	**0.95**
DS27	88.0	88.3	83.8	85.9	84.9	93.4	94	96.4	94.9	**97.2**	0.80	0.83	0.83	0.83	0.83	0.93	0.94	0.92	0.94	**0.95**
DS28	88.0	80.9	89.88	89.9	81.88	90.4	93	91.4	92	**94.9**	0.81	0.80	0.81	0.80	0.81	0.90	0.89	0.91	0.92	**0.93**
DS29	89.3	82.9	89.88	89.9	80.88	94.4	93	92.4	91	**95.9**	0.80	0.81	0.89	0.89	0.80	0.82	0.89	0.90	0.91	**0.92**
DS30	86.0	88.9	89.8	85.9	84.9	93.4	94	93.4	94.9	**97.9**	0.80	0.83	0.83	0.83	0.83	0.93	0.94	0.92	0.94	**0.95**

Significant values are in [bold].

**Table 39:** Accuracy and F-measure using FR6 with 100% labeled dataset.

Accuracy											F-measure									
ID	J48	RF	NB	K-star	AIRS	Y-J48	Y-RF	Y-NB	Y-Kstar	Y-AIRS	J48	RF	NB	K-star	AIRS	Y-J48	Y-RF	Y-NB	Y-Kstar	Y-AIRS
DS1	89.9	89.6	89.9	89.8	89.2	**98.4**	93.9	**98.4**	95.2	**98.4**	0.82	0.84	0.89	0.89	0.89	**0.93**	0.92	**0.93**	0.92	**0.93**
DS2	89.2	89.3	85.8	90.9	92.8	94.4	93.9	96.4	96.9	**98.9**	0.89	0.89	0.85	0.89	0.91	0.92	0.93	0.93	0.93	**0.98**
DS3	89.6	82.8	82.8	82.9	85.8	92.9	94.9	96.4	95.8	**97.9**	0.82	0.82	0.83	0.82	0.80	0.92	0.91	0.92	0.93	**0.98**
DS4	85.3	86.3	86.9	83.9	82.8	92.4	93.3	93.4	92.9	**98.2**	0.82	0.83	0.84	0.84	0.85	0.93	0.90	0.91	0.91	**0.98**
DS5	89.9	84.3	85.9	85.9	86.8	91.4	92.6	93.4	95.9	**97.1**	0.80	0.83	0.84	0.83	0.83	0.93	0.92	0.92	0.93	**0.96**
DS6	89.3	85.8	89.8	85.9	88.9	93.8	94.1	93.4	94.9	**96.8**	0.80	0.83	0.83	0.83	0.83	0.92	0.93	0.92	0.94	**0.96**
DS7	89.9	85.6	89.8	82.9	82.8	91.4	93	92.4	93.9	**95.5**	0.82	0.83	0.84	0.84	0.85	0.92	0.91	0.92	0.92	**0.94**
DS8	84.3	83.3	82.8	81.9	84.8	92.4	93.3	92.4	93	**95.8**	0.82	0.83	0.83	0.84	0.83	0.94	0.90	0.90	0.92	**0.95**
DS9	82.3	89.3	84.6	82.9	84.8	93.4	93.3	93.4	93.9	**96.9**	0.82	0.85	0.84	0.84	0.85	0.93	0.92	0.92	0.92	**0.94**
DS10	86.0	85.3	83.9	84.9	84.6	93.9	96	98.4	94.9	**98.9**	0.83	0.85	0.86	0.88	0.85	0.92	0.92	0.94	0.94	**0.96**
DS11	85.0	83.9	86.8	86.9	89.8	92.4	93.8	93.4	95.9	**98.9**	0.82	0.88	0.88	0.86	0.88	0.93	0.92	0.93	0.92	**0.95**
DS12	88.9	86.3	86.8	84.9	88.8	95.4	98.2	93.4	93	**97.9**	0.86	0.85	0.84	0.83	0.83	0.91	0.90	0.92	0.93	**0.96**
DS13	90.3	89.3	86.8	85.9	86.8	92.4	94.6	94.9	93.5	**97.8**	0.80	0.83	0.89	0.89	0.80	0.90	0.91	0.90	0.91	**0.96**
DS14	85.9	88.3	86.8	88.9	86.8	92.4	93	93.4	96.9	**98.9**	0.82	0.84	0.85	0.85	0.89	0.93	0.92	0.91	0.92	**0.96**
DS15	86.0	86.3	86.8	88.9	89.8	90.4	92	96.4	96.9	**98.9**	0.89	0.82	0.81	0.80	0.82	0.90	0.91	0.91	0.92	**0.95**
DS16	89.3	88.3	89.8	89.9	89.8	92.4	94.9	98.4	98.9	**99.9**	0.80	0.81	0.80	0.82	0.80	0.91	0.89	0.90	0.91	**0.94**
DS17	86.0	84.3	89.8	86.9	89.8	90.4	94.3	96.4	95.9	**98.1**	0.89	0.82	0.81	0.80	0.82	0.90	0.91	0.91	0.92	**0.95**
DS18	89.3	85.3	86.8	84.9	88.9	91.4	93	93.4	93	**93.9**	0.80	0.81	0.85	0.86	0.80	0.90	0.92	0.90	0.91	**0.92**
DS19	88.9	88.3	89.9	89.9	89.0	93.4	93	95.4	94	**97.6**	0.80	0.81	0.89	0.89	0.80	0.90	0.92	0.90	0.91	**0.92**
DS20	89.8	88.3	89.8	86.9	89.8	92.9	96.9	96.4	98.9	**99.9**	0.80	0.81	0.80	0.82	0.86	0.91	0.92	0.90	0.91	**0.94**
DS21	89.9	85.9	88.9	89.9	83.9	92.4	94.9	98.4	98.9	**99.2**	0.80	0.83	0.83	0.83	0.83	0.93	0.94	0.92	0.94	**0.96**
DS22	89.9	84.3	88.6	89.9	89.8	95.4	93	92.4	93.9	**95.9**	0.82	0.83	0.83	0.84	0.82	0.92	0.93	0.92	0.92	**0.95**
DS23	89.0	85.3	84.8	85.9	84.9	92.4	94.3	95.4	94.9	**95.9**	0.80	0.83	0.88	0.89	0.88	0.93	0.94	0.92	0.94	**0.98**
DS24	89.9	89.3	89.8	89.9	89.8	91.4	93	92.4	93.9	**95.2**	0.83	0.84	0.85	0.88	0.84	0.93	0.92	0.93	0.94	**0.95**
DS25	88.3	88.9	88.8	89.9	89.8	93.4	92	92.4	93	**96.5**	0.80	0.81	0.89	0.89	0.80	0.92	0.91	0.92	0.91	**0.93**
DS26	85.0	86.3	83.8	85.9	88.9	94.4	94	93.4	94.9	**95.2**	0.80	0.83	0.83	0.83	0.83	0.93	0.94	0.92	0.94	**0.95**
DS27	88.0	88.3	83.8	85.9	84.9	93.4	94	96.4	94.9	**97.2**	0.80	0.83	0.83	0.83	0.83	0.93	0.94	0.92	0.94	**0.95**
DS28	88.0	80.9	89.88	89.9	81.88	90.4	93	91.4	92	**94.9**	0.81	0.80	0.81	0.80	0.81	0.90	0.89	0.91	0.92	**0.93**
DS29	89.3	82.9	89.88	89.9	80.88	94.4	93	92.4	91	**95.9**	0.80	0.81	0.89	0.89	0.80	0.82	0.89	0.90	0.91	**0.92**
DS30	86.0	88.9	89.8	85.9	84.9	93.4	94	93.4	94.9	**97.9**	0.80	0.83	0.83	0.83	0.83	0.93	0.94	0.92	0.94	**0.95**

Significant values are in [bold].

## Appendix B

**Table 40:** Top malware families used in our data set

ID	Family	# of samples	ID	Family	# of samples	ID	Family	# of samples
A1	Airpush	150	A2	AndroRAT	140	A3	Andup	300
A4	Aples	120	A5	BankBot	100	A6	Bankun	133
A7	Boqx	130	A8	Boxer	122	A9	Cova	100
A10	Dowgin	100	A11	DroidKungFu	100	A12	Erop	120
A13	FakeAngry	110	A14	FakeAV	120	A15	FakeDoc	120
A16	FakeInst	110	A17	FakePlayer	120	A18	FakeTimer	120
A19	FakeUpdates	120	A20	Finspy	111	A21	Fjcon	123
A22	Fobus	102	A23	Fusob	181	A24	GingerMaster	192
A25	GoldDream	20	A26	Gorpo	120	A27	Gumen	20
A28	Jisut	62	A29	Kemoge	720	A30	Koler	200
A31	Ksapp	290	A32	Kuguo	100	A33	Kyview	500
A34	Leech	300	A35	Lnk	100	A36	Lotoor	20
A37	Mecor	29	A38	Minimob	330	A39	Mmarketpay	200
A40	MobileTX	500	A41	Mseg	230	A42	Mtk	200
A43	Nandrobox	100	A44	Obad	100	A45	Opfake	120
A46	Penetho	120	A47	Ramnit	120	A48	Roop	120
A49	RuMMS	100	A50	SimpleLocker	110	A51	SlemBunk	120
A52	SmsKey	120	A53	SMsZombie	110	A54	Spambot	115
A55	SpyBubble	120	A56	Stealer	300	A57	Steek	230
A58	Svpeng	20	A59	Tesbo	21	A60	Triada	200
A61	Univert	210	A62	UpdtKiller	100	A63	Utchi	300
A64	Vidro	92	A65	VikingHorde	230	A66	Vmvol	533
A67	Winge	190	A68	Youmi	689	A69	Zitmo	230
A70	Ztorg	1000	A71	Imlog	50	A72	SMSreg	50
A73	Gappusin	50	A74	Adrd	50	A75	Geinimi	100
A76	Kmin	157	A77	Plankton	125	A78	GingerMaster	100
A79	Iconosys	100	A80	SendPay	18	A81	GoldDream	200

**Table 41:** TPR and FPR for optimal number of labeled dataset.

ID	TPR	FPR
DS1	0.93	0.014
DS2	0.98	0.002
DS3	0.98	0.022
DS4	0.98	0.016
DS5	0.96	0.018
DS6	0.96	0.024
DS7	0.94	0.034
DS8	0.95	0.034
DS9	0.94	0.002
DS10	0.96	0.018
DS11	0.95	0.028
DS12	0.96	0.022
DS13	0.96	0.024
DS14	0.96	0.018
DS15	0.95	0.028
DS16	0.94	0.000
DS17	0.95	0.012
DS18	0.92	0.042
DS19	0.92	0.032
DS20	0.94	0.000
DS21	0.96	0.008
DS22	0.95	0.032
DS23	0.98	0.022
DS24	0.95	0.046
DS25	0.93	0.000
DS26	0.95	0.046
DS27	0.95	0.006
DS28	0.93	0.032
DS29	0.92	0.038
DS30	0.95	0.008

## References

[1] Faruki, P. et al. Android security: a survey of issues, malware penetration, and defenses. *IEEE Commun. Surv. Tutor.* **17**(2), 998–1022 (2014).

[2] Mahindru, A. et al. Permdroid a framework developed using proposed feature selection approach and machine learning techniques for android malware detection. *Sci. Rep.* **14**(1), 10724 (2024). 38730228 10.1038/s41598-024-60982-y PMC11636933

[3] Mahindru, A. & Singh, P. Dynamic permissions based android malware detection using machine learning techniques. In *Proceedings of the 10th innovations in Software Engineering Conference* 202–210 (2017).

[4] Huda, S. et al. Defending unknown attacks on cyber-physical systems by semi-supervised approach and available unlabeled data. *Inf. Sci.* **379**, 211–228 (2017).

[5] Zhang, K., Li, C., Wang, Y., Zhu, X. & Wang, H. Collaborative support vector machine for malware detection. In *International Conference on Computational Science* 1682–1691 (2017)

[6] Chen, L., Zhang, M., Yang, C. Y. & Sahita, R. Semi-supervised classification for dynamic android malware detection (2017) arXiv:1704.05948 arXiv preprint.

[7] Driessens, K., Reutemann, P., Pfahringer, B. & Leschi, C. Using weighted nearest neighbor to benefit from unlabeled data. In *Pacific-Asia Conference on Knowledge Discovery and Data Mining* 60–69 (Springer, 2006).

[8] Burguera, I., Zurutuza, U. & Nadjm-Tehrani, S. Crowdroid: behavior-based malware detection system for android. In *Proceedings of the 1st ACM Workshop on Security and Privacy in Smartphones and Mobile Devices* 15–26 (2011).

[9] Shabtai, A., Kanonov, U., Elovici, Y., Glezer, C. & Weiss, Y. “andromaly’’: a behavioral malware detection framework for android devices. *J. Intell. Inf. Syst.* **38**(1), 161–190 (2012).

[10] Aafer, Y., Du, W. & Yin, H. Droidapiminer: mining api-level features for robust malware detection in android. In *International Conference on Security and Privacy in Communication Systems* 86–103 (Springer, 2013).

[11] Aung, W. Z. Z. Permission-based android malware detection. *Int. J. Sci. Technol. Res.* **2**(3), 228–234 (2013).

[12] Yuan, Z., Lu, Y., Wang, Z. & Xue, Y. Droid-sec: deep learning in android malware detection. In *Proceedings of the 2014 ACM conference on SIGCOMM* 371–372 (2014).

[13] Yerima, S. Y., Sezer, S. & McWilliams, G. Analysis of bayesian classification-based approaches for android malware detection. *IET Inf. Secur.* **8**(1), 25–36 (2014).

[14] Liang, S. & Du, X. Permission-combination-based scheme for android mobile malware detection. In *IEEE International Conference on Communications (ICC)* 2301–2306 (IEEE, 2014).

[15] Talha, K. A., Alper, D. I. & Aydin, C. Apk auditor: permission-based android malware detection system. *Digital Investig.* **13**, 1–14 (2015).

[16] Canfora, G., Medvet, E., Mercaldo, F. & Visaggio, C. A. Detecting android malware using sequences of system calls. In *Proceedings of the 3rd International Workshop on Software Development Lifecycle for Mobile* 13–20 (2015).

[17] Yerima, S. Y., Sezer, S. & Muttik, I. High accuracy android malware detection using ensemble learning. *IET Inf. Secur.* **9**(6), 313–320 (2015).

[18] Shrivastava, G. & Kumar, P. Sensdroid: analysis for malicious activity risk of android application. *Multimedia Tools Appl.* **78**(24), 35713–35731 (2019).

[19] Sharma, K. & Gupta, B. B. Mitigation and risk factor analysis of android applications. *Comput. Electr. Eng.* **71**, 416–430 (2018).

[20] Deepserish, B. et al. Pet-droid: android malware detection using static analysis. In *2022 4th International Conference on Advances in Computing, Communication Control and Networking (ICAC3N)* 2473–2480 (IEEE, 2022).

[21] Dahiya, A., Singh, S. & Shrivastava, G. Prazdroid: a novel approach to risk assessment and zoning of android applications based on permissions. *Scalable Comput.: Pract. Exp.* **26**(4), 1559–1576 (2025).

[22] Khari, M., Dalal, R., Misra, U. & Kumar, A. Androset: an automated tool to create datasets for android malware detection and functioning with wot. In *Smart Innovation of Web of Things* 187–206 (CRC Press, 2020).

[23] Sun, L., Li, Z., Yan, Q., Srisa-an, W., & Pan, Y. Sigpid: significant permission identification for android malware detection. In *2016 11th International Conference on Malicious and Unwanted Software (MALWARE)* 1–8 (IEEE, 2016)

[24] Chen, S., Xue, M., Tang, Z., Xu, L. & Zhu, H. Stormdroid: a streaminglized machine learning-based system for detecting android malware. In *Proceedings of the 11th ACM on Asia Conference on Computer and Communications Security* 377–388 (2016).

[25] Hou, S., Ye, Y., Song, Y. & Abdulhayoglu, M. Hindroid: an intelligent android malware detection system based on structured heterogeneous information network. In *Proceedings of the 23rd ACM SIGKDD International Conference on Knowledge Discovery and Data Mining* 1507–1515 (2017).

[26] McLaughlin, N. et al. Deep android malware detection. In *Proceedings of the Seventh ACM on Conference on Data and Application Security and Privacy* 301–308 (2017)

[27] Milosevic, N., Dehghantanha, A. & Choo, K. K. R. Machine learning aided android malware classification. *Comput. Electr. Eng.* **61**, 266–274 (2017).

[28] Kim, J., Ban, Y., Ko, E., Cho, H. & Yi, J. H. Mapas: a practical deep learning-based android malware detection system. *Int. J. Inf. Secur.* **21**(4), 725–738 (2022).

[29] Zhu, H. J. et al. Droiddet: effective and robust detection of android malware using static analysis along with rotation forest model. *Neurocomputing* **272**, 638–646 (2018).

[30] Shang, F., Li, Y., Deng, X. & He, D. Android malware detection method based on naive bayes and permission correlation algorithm. *Cluster Comput.* **21**(1), 955–966 (2018).

[31] Wang, W., Zhao, M. & Wang, J. Effective android malware detection with a hybrid model based on deep autoencoder and convolutional neural network. *J. Ambient Intell. Human. Comput.* **10**(8), 3035–3043 (2019).

[32] Zou, D. et al. Intdroid: Android malware detection based on api intimacy analysis. *ACM Trans. Softw. Eng. Methodol. (TOSEM)* **30**(3), 1–32 (2021).

[33] Gao, H., Cheng, S. & Zhang, W. Gdroid: android malware detection and classification with graph convolutional network. *Comput. Secur.* **106**, 102264 (2021).

[34] Jerbi, M., Dagdia, Z. C., Bechikh, S. & Said, L. B. Android malware detection as a bi-level problem. *Comput. Secur.* **121**, 102825 (2022).

[35] Golmaryami, M., Taheri, R., Pooranian, Z., Shojafar, M. & Xiao, P. Setti: as elf-supervised adv e rsarial malware de t ection archi t ecture in an i ot environment. *ACM Trans. Multimedia Comput.* **18**(2s), 1–21 (2022).

[36] Eslamnejad, M., Taheri, R., Shojafar, M. & Bader-El-Den, M. Federated learning-based robust android malware detection: label-flipping attacks and defenses. *Neural Comput. Appl.* **37**(32), 27057–27082 (2025).

[37] Wu, Y. et al. Droidrl: feature selection for android malware detection with reinforcement learning. *Comput. Secur.* **128**, 103126 (2023).

[38] Almin, S. B. & Chatterjee, M. A novel approach to detect android malware. *Procedia Comput. Sci.* **45**, 407–417 (2015).

[39] Sheen, S., Anitha, R. & Natarajan, V. Android based malware detection using a multifeature collaborative decision fusion approach. *Neurocomputing* **151**, 905–912 (2015).

[40] Andriatsimandefitra, R. & Tong, V. Detection and identification of android malware based on information flow monitoring. In *IEEE 2nd International Conference on Cyber Security and Cloud Computing* 200–203 (IEEE, 2015).

[41] Caviglione, L., Gaggero, M., Lalande, J. F., Mazurczyk, W. & Urbański, M. Seeing the unseen: revealing mobile malware hidden communications via energy consumption and artificial intelligence. *IEEE Trans. Inf. Forens. Secur.* **11**(4), 799–810 (2015).

[42] Holland, B., Deering, T., Kothari, S., Mathews, J. & Ranade, N. Security toolbox for detecting novel and sophisticated android malware. In *2015 IEEE/ACM 37th IEEE International Conference on Software Engineering* 733–736 (IEEE, 2015)

[43] Shen, T., Zhongyang, Y., Xin, Z., Mao, B. & Huang, H. Detect android malware variants using component based topology graph. In *IEEE 13th International Conference on Trust, Security and Privacy in Computing and Communications* 406–413 (IEEE, 2014).

[44] Rahman, M. Droidmln: a markov logic network approach to detect android malware. In *12th International Conference on Machine Learning and Applications, vol. 2* 166–169 (IEEE, 2013).

[45] Martinelli, F., Mercaldo, F. & Saracino, A. Bridemaid: an hybrid tool for accurate detection of android malware. In *Proceedings of the 2017 ACM on Asia Conference on Computer and Communications Security* 899–901 (2017).

[46] Ng, D. V. & Hwang, J. Android malware detection using the dendritic cell algorithm. In *2014 International Conference on Machine Learning and Cybernetics, Vol. 1* 257–262 (IEEE, 2014).

[47] Alam, M. S. & Vuong, S. T. Random forest classification for detecting android malware. In *2013 IEEE international conference on green computing and communications and IEEE Internet of Things and IEEE cyber, physical and social computing* 663–669 (IEEE, 2013).

[48] Amos, B., Turner, H. & White, J. Applying machine learning classifiers to dynamic android malware detection at scale. In *2013 9th International Wireless Communications and Mobile Computing Conference (IWCMC)* 1666–1671 (IEEE, 2013).

[49] Wei, T. E. et al. Android malware detection via a latent network behavior analysis. In *IEEE 11th International Conference on Trust, Security and Privacy in Computing and Communications* 1251–1258 (IEEE, 2012).

[50] Tong, F. & Yan, Z. A hybrid approach of mobile malware detection in android. *J. Parallel Distrib. Comput.* **103**, 22–31 (2017).

[51] Yang, L., Ganapathy, V. & Iftode, L. Enhancing mobile malware detection with social collaboration. In *IEEE Third International Conference on Privacy, Security, Risk and Trust and 2011 IEEE Third International Conference on Social Computing* 572–576 (IEEE, 2011).

[52] Kadir, A., Stakhanova, N. & Ghorbani, A. A. Android botnets: what urls are telling us. In *International Conference on Network and System Security* 78–91 (Springer, 2015).

[53] Zhou, Y. & Jiang, X. Dissecting android malware: characterization and evolution. In *2012 IEEE Symposium on Security and Privacy* 95–109 (IEEE, 2012).

[54] Wu, D. J. et al. Droidmat: android malware detection through manifest and api calls tracing. In *2012 Seventh Asia Joint Conference on Information Security* 62–69 (IEEE, 2012)

[55] Allix, K. et al. Empirical assessment of machine learning-based malware detectors for android. *Empirical Softw. Eng.* **21**(1), 183–211 (2016).

[56] Mas’ud, M. Z., Sahib, S., Abdollah, M. F., Selamat, S. R., & Yusof, R. Analysis of features selection and machine learning classifier in android malware detection. In *2014 International Conference on Information Science & Applications (ICISA)* 1–5 (IEEE, 2014)

[57] Narayanan, A., Chandramohan, M., Chen, L. & Liu, Y. A multi-view context-aware approach to android malware detection and malicious code localization. *Empirical Softw. Eng.* **23**(3), 1222–1274 (2018).

[58] Yerima, SY., Sezer, S., McWilliams, G., & Muttik, I. A new android malware detection approach using bayesian classification. In *2013 IEEE 27th international conference on advanced information networking and applications (AINA)* 121–128 (IEEE, 2013)

[59] Freijeiro-González, L., Febrero-Bande, M. & González-Manteiga, W. A critical review of lasso and its derivatives for variable selection under dependence among covariates. *Int. Stat. Rev.* **90**(1), 118–145 (2022).

[60] Muvunza, T., Kraev, E., Planell-Morell, P. & Shestopaloff, A. Y. Minerva: mutual information neural estimation for supervised feature selection (2025) arXiv:2510.02610 arXiv preprint.

[61] Vergara, J. R. & Estévez, P. A. A review of feature selection methods based on mutual information. *Neural Comput. Appl.* **24**(1), 175–186 (2014).

[62] Verleysen, M., Rossi, F. & François, D. Advances in feature selection with mutual information. In *Similarity-Based Clustering: Recent Developments and Biomedical Applications* 52–69 (Springer, 2009).

[63] Zhongxin, W., Gang, S., Jing, Z. & Jia, Z. Feature selection algorithm based on mutual information and lasso for microarray data. *Open Biotechnol. J.* **10**, 1 (2016).

[64] Novakovic, J. The impact of feature selection on the accuracy of naive bayes classifier. In *18th Telecommunications forum TELFOR, Vol. 2* 1113–1116 (2010).

[65] Plackett, R. L. Karl pearson and the chi-squared test. *Int. Stat. Rev.* **1983**, 59–72 (1983).

[66] Maćkiewicz, A. & Ratajczak, W. Principal components analysis (pca). *Comput. Geosci.* **19**(3), 303–342 (1993).

[67] Sperandei, S. Understanding logistic regression analysis. *Biochem. Med.* **24**(1), 12–18 (2014). 10.11613/BM.2014.003 PMC3936971 24627710

[68] Watkins, A. B. Airs: A resource limited artificial immune classifier (Mississippi State University Mississippi, 2001) (PhD thesis).

[69] Catal, C. & Diri, B. A fault prediction model with limited fault data to improve test process. In *International Conference on Product Focused Software Process Improvement* 244–257 (Springer, 2008).

[70] Catal, C. & Diri, B. Software fault prediction with object-oriented metrics based artificial immune recognition system. In *International Conference on Product Focused Software Process Improvement* 300–314 (Springer, 2007).

[71] Castro, L. N., Castro, L. N. D. & Timmis, J. *Artificial Immune Systems: A New Computational Intelligence Approach* (Springer Science and Business Media, 2002)

[72] Watkins, A., Timmis, J. & Boggess, L. Artificial immune recognition system (airs): an immune-inspired supervised learning algorithm. *Genetic Program. Evol. Mach.* **5**(3), 291–317 (2004).

[73] Kohavi, R. et al. Wrappers for feature subset selection. *Artif. Intell.* **97**(1–2), 273–324 (1997).

[74] Faruki, P., Ganmoor, V., Laxmi, V., Gaur, M. S. & Bharmal, A. Androsimilar: robust statistical feature signature for android malware detection. In *Proceedings of the 6th International Conference on Security of Information and Networks* 152–159 (2013).

[75] Lindorfer, M. et al. Andrubis–1,000,000 apps later: a view on current android malware behaviors. In *2014 Third International Workshop on Building Analysis Datasets and Gathering Experience Returns for Security (BADGERS)* pp 3–17 (IEEE, 2014)

[76] Xu, R., Saidi, H. & Anderson, R. Aurasium: practical policy enforcement for android applications. In *Presented as part of the 21st USENIX Security Symposium USENIX Security 12)* 539–552 (2012).

[77] Tam, K., Khan, S. J., Fattori, A. & Cavallaro, L. Copperdroid: automatic reconstruction of android malware behaviors (Ndss, 2015).

[78] Portokalidis, G., Homburg, P., Anagnostakis, K. & Bos, H. Paranoid android: versatile protection for smartphones. In *Proceedings of the 26th Annual Computer Security Applications Conference* 347–356 (2010).

[79] Enck, W. et al. Taintdroid: an information-flow tracking system for realtime privacy monitoring on smartphones. *ACM Trans. Comput. Syst. (TOCS)* **32**(2), 1–29 (2014).

[80] Karbab, E. B., Debbabi, M., Derhab, A. & Mouheb, D. Maldozer: automatic framework for android malware detection using deep learning. *Digital Investig.* **24**, S48–S59 (2018).

[81] Zhu, H. J. et al. Hemd: a highly efficient random forest-based malware detection framework for android. *Neural Comput. Appl.* **30**(11), 3353–3361 (2018).

[82] Han, W., Xue, J., Wang, Y., Liu, Z. & Kong, Z. Malinsight: a systematic profiling based malware detection framework. *J. Netw. Comput. Appl.* **125**, 236–250 (2019).

[83] Arp, D. et al. Drebin: effective and explainable detection of android malware in your pocket. *Ndss* **14**, 23–26 (2014).

